# Optimal Combinations of AT(N) Biomarkers to Determine Longitudinal Cognition in the Alzheimer's Disease

**DOI:** 10.3389/fnagi.2021.718959

**Published:** 2021-08-06

**Authors:** Rong-Rong Lin, Yan-Yan Xue, Xiao-Yan Li, Yi-He Chen, Qing-Qing Tao, Zhi-Ying Wu

**Affiliations:** Department of Neurology and Research Center of Neurology in Second Affiliated Hospital, and Key Laboratory of Medical Neurobiology of Zhejiang Province, Zhejiang University School of Medicine, Hangzhou, China

**Keywords:** Alzheimer's disease, biomarkers, AT(N) system, longitudinal cognition, linear mixed-effects model

## Abstract

**Background:** National Institute on Aging—Alzheimer's Association (NIA-AA) proposed the AT(N) system based on β-amyloid deposition, pathologic tau, and neurodegeneration, which considered the definition of Alzheimer's disease (AD) as a biological construct. However, the associations between different AT(N) combinations and cognitive progression have been poorly explored systematically. The aim of this study is to compare different AT(N) combinations using recognized biomarkers within the Alzheimer's Disease Neuroimaging Initiative (ADNI) cohort.

**Methods:** A total of 341 participants were classified into cognitively unimpaired (CU; *n* = 200) and cognitively impaired (CI; *n* = 141) groups according to the clinical manifestations and neuropsychological tests. Cerebrospinal fluid (CSF) Aβ42 and amyloid-PET ([18F]flutemetamol) were used as biomarkers for A; CSF phosphorylated tau (p-tau) and tau-PET ([18F]flortaucipir) were used as biomarkers for T; CSF total tau (t-tau), hippocampal volume, temporal cortical thickness, [18F]fluorodeoxyglucose (FDG) PET, and plasma neurofilament light (NfL) were used as biomarkers for (N). Binary biomarkers were obtained from the Youden index and publicly available cutoffs. Prevalence of AT(N) categories was compared between different biomarkers within the group using related independent sample non-parametric test. The relationship between AT(N) combinations and 12-year longitudinal cognition was assessed using linear mixed-effects modeling.

**Results:** Among the CU participants, A–T–(N)– was most common. More T+ were detected using p-tau than tau PET (*p* < 0.05), and more (N)+ were observed using fluid biomarkers (*p* < 0.001). A+T+(N)+ was more common in the CI group. Tau PET combined with cortical thickness best predicted cognitive changes in the CI group and MRI predicted changes in the CU group.

**Conclusions:** These findings suggest that optimal AT(N) combinations to determine longitudinal cognition differ by cognitive status. Different biomarkers within a specific component for defining AT(N) cannot be used identically. Furthermore, different strategies for discontinuous biomarkers will be an important area for future studies.

## Introduction

Alzheimer's disease (AD) is the most common cause of dementia, and one of the main causes of complications and death in the aging population. A series of complex pathobiological processes is involved in the pathogenesis of AD, including the deposition of extracellular amyloid plaques, tau-related intracellular neurofibrillary tangles (NFTs), neuronal loss and atrophy (Long and Holtzman, 2019). Recently, the National Institute on Aging—Alzheimer's Association (NIA-AA) proposed a research framework based on the pathological characteristics mentioned above (Jack et al., 2018). The framework establishes a classification system consisting of biomarkers of Aβ (A), tau (T), and neurodegeneration (N), and lists a classic AD biomarker grouping including cerebrospinal fluid (CSF), MRI, and PET. However, it is not perfectly concordant among biomarkers within a specific component (A, T, or N) (Jack et al., 2018; Knopman et al., 2018), and all examinations are usually difficult to perform on patients, which may limit its clinical application. Many studies have compared different biomarkers in a certain component (Mattsson et al., 2015; Hansson et al., 2018; La Joie et al., 2018), and have manifested that these biomarkers partially play different roles in the diagnosis, staging, and the progression of Alzheimer's pathology. For example, CSF is suitable for early diagnosis (Mattsson et al., 2015), and tau PET is related to cross-sectional cognition of AD patients (La Joie et al., 2018). This means that different biomarkers need to be selected according to different clinical needs. But only one study assessed different combinations of AT(N) biomarkers using BioFINDER participants (Mattsson-Carlgren et al., 2020). Here, we used a more comprehensive biomarker group and focused on the relationship between different AT(N) combinations and longitudinal cognition decline. We postulated that the prevalence of AT(N) categories and prediction of longitudinal cognition would vary by different combinations of biomarkers in cognitively unimpaired (CU) and cognitively impaired (CI) participants.

## Materials and Methods

### Participants

All participants in this study were from the Alzheimer's Disease Neuroimaging Initiative (ADNI), a longitudinal multicenter study designed to develop clinical, imaging, genetic, and biospecimen biomarkers for tracking the progression of AD. Regional ethics committees of all institutions approved the ADNI study. Written informed consent was obtained from all participants. The key eligibility criteria were based on the ADNI protocol. Cognitively unimpaired (CU) participants must be free of memory complaints and cognitively normal, with MMSE scores between 24 and 30 (inclusive) and a CDR of 0. Cognitively impaired (CI) participants must have a subjective memory concern and were divided into two subgroups, namely: mild cognitively impaired (MCI) participants and AD dementia participants. The MCI participants reported MMSE scores between 24 and 30 (inclusive), a memory complaint, have objective memory loss measured by education-adjusted scores on Wechsler memory scale logical memory II, a CDR of 0.5, absence of significant levels of impairment in other cognitive domains, essentially preserved activities of daily living, and an absence of dementia. The AD dementia participants met NINCDS/ADRDA criteria for probable AD (McKhann et al., 1984), with MMSE scores between 20 and 26 (inclusive) and a CDR of 0.5 or 1.0. Demographic and clinical information, neuroimaging, and biomarker data were downloaded from the ADNI data repository (adni.loni.usc.edu).

### CSF and Plasma Biomarker Analysis

Cerebrospinal fluid β-amyloid (1-42), phospho-tau (181P), and total tau were analyzed using the electrochemiluminescence immunoassays (ECLIA) Elecsys following a Roche Study Protocol (Hansson et al., 2018). Plasma neurofilament light (NfL) was obtained using the single molecule array (Simoa) technique.

### Neuroimaging Acquisition and Processing

The 3T MRI scans were processed before being downloaded as previously described (Jack et al., 2008, 2010). FreeSurfer (ADNI phase 1, grand opportunity, and phase 2 data were run with FreeSurfer version 5.1, while phase 3 with version 6.0) was used for further analysis. Two MRI measures were used, including hippocampal volume and cortical thickness. The volume of bilateral hippocampal was extracted as the regions of interest (ROI), and was adjusted for the intracranial volume (ICV) by calculating the residual term (ε) from a linear regression of hippocampal volume (*y*) vs. ICV (*x*) within 128 ApoE-negative CU participants (Jack et al., 2014). The adjusted hippocampal volume can be interpreted as a deviation from the expected hippocampal volume calculated from the given ICV. An AD signature cortical thickness was composed of mean thickness in the entorhinal, inferior temporal, middle temporal, and fusiform cortices (Dickerson et al., 2009).

Amyloid, tau, and metabolic imaging were performed using [18F]florbetapir, [18F]flortaucipir and [18F]fluorodeoxyglucose (FDG) PET, respectively. The [18F]florbetapir standardized uptake value ratios (SUVRs) were calculated by averaging the four cortical regions, frontal, which are anterior/posterior cingulate, lateral parietal, and lateral temporal cortices (Klunk et al., 2004; Xue et al., 2020), and dividing the ROIs by the whole cerebellum reference region. For tau PET, the inferior temporal cortex (ITC) and the Braak V/VI region (specific regions were shown in Supplementary Table 1) were selected as target ROIs. ITC and Braak V/VI indicated early and late stages of tangle pathology, respectively (Braak et al., 2006; Johnson et al., 2016). The [18F]flortaucipir data were corrected for partial volume effects using the geometric transfer matrix approach and divided by the inferior cerebellar GM reference region (Baker et al., 2017). The predefined meta-ROIs in FDG PET of AD were composed of the angular gyrus, posterior cingulate, and ITC normalized to the pons and vermis (Herholz et al., 2002).

### Cognition Assessment

Cognition was assessed using the longitudinal Mini-Mental State Examination (MMSE) and Clinical Dementia Rating Sum of Boxes (CDRSB). According to the interquartile range (IQR; 6–8 years), we selected seven-time points from baseline to 12 years (baseline, 2, 4, 6, 8, 10, and 12 years, respectively) for the longitudinal cognitive assessment.

### AT(N) Definitions

AT(N) biomarkers included CSF Aβ42 (A1), amyloid PET ([18F]florbetapir) (A2), CSF p-tau (T1), tau PET ([18F]flortaucipir) SUVR in the ITC (T2) and Braak V/VI region (T3), hippocampal volume ([N]1), temporal meta-ROI cortical thickness ([N]2), CSF t-tau ([N]3), AD-characteristic FDG PET SUVR ([N]4), and plasma NfL ([N]5). For CSF Aβ positivity, we used a published cutoff (CSF Aβ42 level, <880 ng/L; Hansson et al., 2018). For amyloid PET, we selected a cutoff of 1.11, which is the upper 95% confidence interval above the mean of a young normal control group (Joshi et al., 2012). Binarization of other biomarkers (T and [N]) was performed using cutoffs established by the Youden index (Aβ-positive MCI vs. Aβ-negative CU, with the Aβ status defined by the CSF Aβ42). Furthermore, the mean ± 2 SD from Aβ-negative CU controls (+2 SD for amyloid PET, tau PET, CSF tau, and plasma NfL; −2 SD for CSF Aβ42, hippocampal volume, temporal cortical thickness, and FDG PET), along with 90% sensitivity for AD, were used as a sensitivity analysis.

### Statistical Analyses

Demographics and continuous biomarkers were compared between different groups using Mann–Whitney *U*-test or Student's *t*-test according to normality of the population sample using Kolmogorov-Smirnov *Z*-test, and binary biomarkers using Fisher's exact test. Associations between biomarkers were analyzed using Spearman's rank correlation (*ρ*), Cohen's kappa coefficient (κ), and percentage agreement (concordance). Prevalence estimates for AT(N) categories were calculated with 95% confidence intervals generated using bootstrap resampling (*n* = 1,000). Prevalence of AT(N) categories was compared between different biomarkers within the group using related independent sample non-parametric test (McNemar test for A and Cochran's *Q*-test for T or [N]). The relationships between AT(N) combinations and cognitive trajectories (12-year longitudinal MMSE and CDRSB scores) were examined using a linear mixed-effects (LME) model (including age, sex, and education as covariates, and time as a categorical variable) with subject-specific intercepts and slopes. The goodness of LME models with different AT(N) combinations was assessed by marginal R^2^, which represented the fixed effect of LME models. All analyses were performed using IBM SPSS Statistics 20, with significance of the two-tailed test set to *p* < 0.05.

## Results

### Study Participants

Demographics are presented in Table 1, and more detailed information is shown in Supplementary Table 2. A significant difference in age was not observed between CU and CI participants, while more females, higher education level, and a lower prevalence of APOE e4 were observed in the CU group. No significant differences were observed in age, sex, education, or APOE e4 between participants with MCI and AD (subgroups of CI). MMSE scores, Aβ42, hippocampal volume, temporal cortical thickness, and FDG PET decreased sequentially, while CDRSB scores, amyloid and tau PET, and CSF tau and NfL increased sequentially among the CU, MCI, CI, and AD groups. As plasma NfL levels were reported to be positively associated with age (ρ = 0.471, *p* < 0.01) (Mattsson et al., 2017a, 2019), we divided participants into younger and older groups based on the median value (age = 72.25 y) and identified a significant difference in NfL levels between these groups (*p* < 0.001). Therefore, the prevalence of (N)+ using NfL was likely to vary by age in the present cohort, so we calculated the cutoff based on age stratification.

**Table 1:** Characteristics of ADNI participants.

	**CU**	**CI**	**P**	**MCI**	**AD**	**P**
No.	200	141	CU vs. CI	101	40	MCI vs. AD
Age at baseline, y^a^	70.95 ± 6.27	72.25 ± 7.08	0.074417	72.47 ± 6.69	71.72 ± 8.06	0.57102
Female	115 (57.5%)	60 (42.6%)	<0.05	41 (40.6%)	19 (47.5%)	0.454664
Education, y	16.77 ± 2.39	15.98 ± 2.82	<0.05	16.21 ± 2.98	15.40 ± 2.31	0.077458
ApoE e4 positive	71 (35.7%)	61 (43.3%)	<0.01	38 (37.6%)	23 (57.5%)	0.097857
MMSE at baseline	29.11 ± 1.10	27.61 ± 2.32	<0.0001	28.15 ± 1.74	26.28 ± 2.99	<0.0001
CDRSB at baseline	0.13 ± 0.47	1.32 ± 1.11	<0.0001	0.98 ± 0.65	2.19 ± 1.51	<0.0001
CSF Aβ42, ng/L	1332.84 ± 643.16	1029.24 ± 660.36	<0.0001	1159.51 ± 703.67	700.33 ± 375.32	<0.0001
CSF Aβ42 positive	61 (30.5%)	82 (58.2%)	<0.0001	48 (47.5%)	34 (85.0%)	<0.0001
Amyloid PET SUVR	1.11 ± 0.18	1.26 ± 0.26	<0.0001	1.20 ± 0.24	1.42 ± 0.22	<0.0001
Amyloid PET positive	63 (31.5%)	92 (65.2%)	<0.0001	56 (55.4%)	36 (90.0%)	<0.0001
CSF p-tau, ng/L	22.21 ± 10.85	29.65 ± 17.95	<0.0001	26.67 ± 15.16	37.15 ± 22.06	<0.001
CSF p-tau positive	83 (41.5%)	95 (67.4%)	<0.0001	62 (61.4%)	33 (82.5%)	<0.05
Tau PET in ITC SUVR	1.99 ± 0.28	2.65 ± 1.20	<0.0001	2.28 ± 0.74	3.60 ± 1.57	<0.0001
Tau PET in ITC positive	41 (20.5%)	80 (56.7%)	<0.0001	46 (45.5%)	34 (85.0%)	<0.0001
Tau PET in Braak5/6 SUVR	1.81 ± 0.19	2.17 ± 0.70	<0.0001	1.95 ± 0.33	2.72 ± 1.02	<0.0001
Tau PET in Braak5/6 positive	40 (20.0%)	72 (51.1%)	<0.0001	40 (39.6%)	32 (80.0%)	<0.0001
Hippocampal volume, cm^3^	−0.059 ± 0.808	−1.09 ± 1.11	<0.0001	−0.80 ± 1.03	−1.83 ± 0.95	<0.0001
Hippocampal volume positive	51 (25.5%)	95 (67.4%)	<0.0001	60 (59.4%)	35 (87.5%)	<0.001
Temporal meta-ROI thickness, mm	3.01 ± 0.15	2.80 ± 0.28	<0.0001	2.86 ± 0.25	2.65 ± 0.28	<0.0001
Temporal meta-ROI thickness positive	41 (20.6%)	90 (63.8%)	<0.0001	56 (55.4%)	34 (85.0%)	<0.01
CSF t-tau, ng/L	244.13 ± 98.70	306.61 ± 152.66	<0.0001	281.77 ± 130.47	369.34 ± 185.41	<0.01
CSF t-tau positive	90 (45.0%)	95 (67.4%)	<0.0001	62 (61.4%)	33 (82.5%)	<0.01
FDG-PET meta-ROI SUVR^a^	1.33 ± 0.11	1.22 ± 0.14	<0.0001	1.26 ± 0.13	1.11 ± 0.12	<0.0001
FDG-PET meta-ROI SUVR positive	34 (24.1%)	85 (62.0%)	<0.0001	52 (52.5%)	33 (86.8%)	<0.0001
plasma NfL, ng/L	35.92 ± 15.72	43.66 ± 20.62	<0.01	41.82 ± 20.90	48.28 ± 19.44	<0.05
plasma NfL positive	66 (47.8%)	81 (69.8%)	<0.01	52 (62.7%)	29 (87.9%)	<0.01

*Aβ, β-amyloid; amyloid PET, [18F]florbetapir PET; CDRSB, Clinical Dementia Rating Sum of Boxes; CI, cognitively impaired; CU, cognitively unimpaired; FDG-PET, [18F]fluorodeoxyglucose PET; ITC, inferior temporal cortex; MMSE, Mini-Mental State Examination; NfL, neurofilament light; p-tau, phosphorylated at Thr181; ROI, region of interest; tau PET, [18F]flortaucipir PET; SUVR, standardized uptake value ratio. Data are presented as mean (SD) or n (%)*.

a *The population sample was normally distributed using Kolmogorov-Smirnov Z-test, continuous biomarkers were compared between different groups using Student's t-test*.

### Biomarker Relationships

Cutoffs were defined as CSF Aβ42 <880 ng/L (A1), amyloid PET >1.1 SUVR (A2), p-tau > 21.11 ng/L (T1), ITC tau PET >2.122 SUVR (T2), Braak V/VI tau PET >1.938 SUVR (T3), adjusted hippocampal volume < -0.4477 cm^3^ (N1), temporal meta-ROI thickness <2.9214 mm (N2), CSF t-tau > 233.6 ng/L (N3), FDG PET meta-ROIs <1.2599 SUVR (N4), and plasma NfL levels >30.35 ng/L in younger participants and >36.45 ng/L in older participants. Similar cutoffs were obtained using 90% sensitivity for AD, while mean ± 2 SD from Aβ-negative CU controls resulted in more conservative cutoffs (Supplementary Table 3).

**Figure 1:**
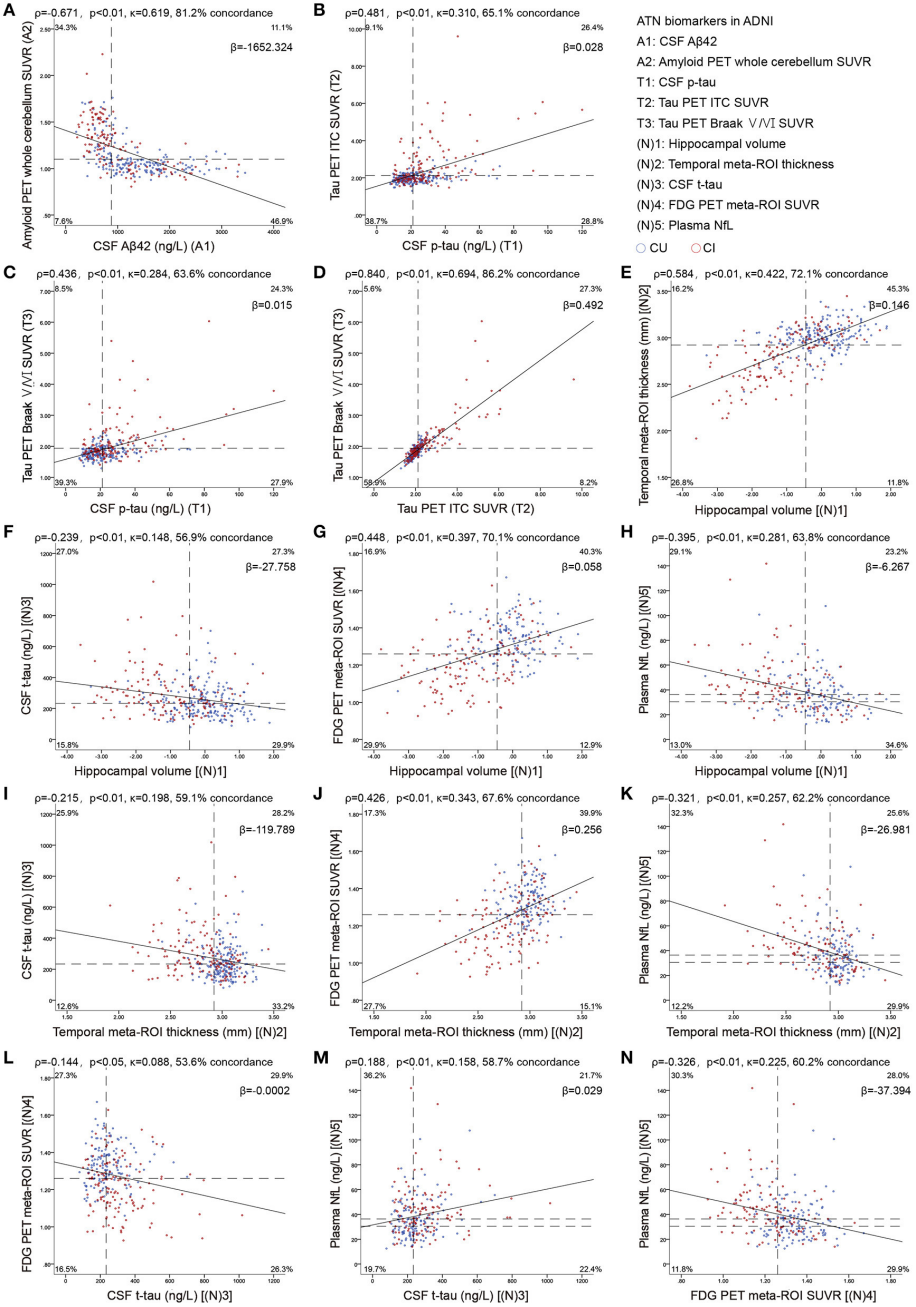
Scatterplots show the association between continuous measures for amyloid **(A)**, tau **(B–D)**, and neurodegeneration **(E–N)** biomarkers. Dashed lines indicate cutoff points. Spearman's correlations (ρ) with *p*-values, Cohen's kappa statistic (κ), concordance (percentage showing both biomarkers positive or negative), and the slope (β) of regression curve are shown at the top of each panel. For A comparisons, the upper left and the lower right quadrants indicate concordance positive (+/+) and negative (–/–). For T comparisons, lower left and upper right quadrants indicate concordance positive and negative, respectively. For the comparisons of (N)3 with (N)5, the upper right and lower left quadrants indicate concordance positive and negative, respectively. For the four remaining (N) comparisons, concordant positives are shown in the upper left quadrant, whereas concordant negatives are shown in the lower right quadrant. Percentage figures across quadrants indicate distribution (percentagewise) of participants. Aβ, β-amyloid; AT(N), β-amyloid, tau, and neurodegeneration classification system; CI, cognitively impaired; CU, cognitively unimpaired; ITC, inferior temporal cortex; NfL, neurofilament light; p-tau, tau phosphorylated at Thr181; ROI, region of interest; SUVR, standardized uptake value ratio; t-tau, total tau.

Continuous biomarkers within each component were correlated: CSF Aβ42 vs. amyloid PET (ρ = −0.671; Figure 1A), p-tau vs. ITC tau PET (ρ = 0.379) and Braak V/VI (ρ = 0.380), as well as between the 2 tau PET measures (ρ = 0.851; Figures 1B–D); hippocampal volume vs. temporal cortical thickness (ρ = 0.584), vs. FDG PET (ρ = 0.448), and vs. NfL (ρ = −0.395); temporal cortical thickness vs. FDG PET (ρ = 0.426), and vs. NfL (ρ = −0.321); and FDG PET vs. NfL (ρ = −0.326). Weak correlations were observed between CSF t-tau and other neurodegeneration biomarkers: CSF t-tau vs. hippocampal volume (ρ = −0.239), vs. temporal cortical thickness (ρ = −0.215), vs. FDG PET (ρ = −0.145, *p* < 0.05) and vs. NfL (ρ = 0.188; all *p* < 0.001, except as specifically indicated; Figures 1E–N).

Using binary data, there was a substantial agreement between amyloid biomarkers (Figure 1A), between the two tau PET measures (Figures 1B–D), and a moderate agreement between the two MRI imaging measures (Figure 1E). Fair agreement was identified between p-tau and tau PET (Figures 1B,C), between MRI imaging measures, FDG PET, and NfL (Figures 1G,H,J,K,N), whereas slight agreement between CSF t-tau and other neurodegeneration biomarkers (Figures 1F,I,L,M).

### Prevalence Measures in CU Participants

**Figure 2:**
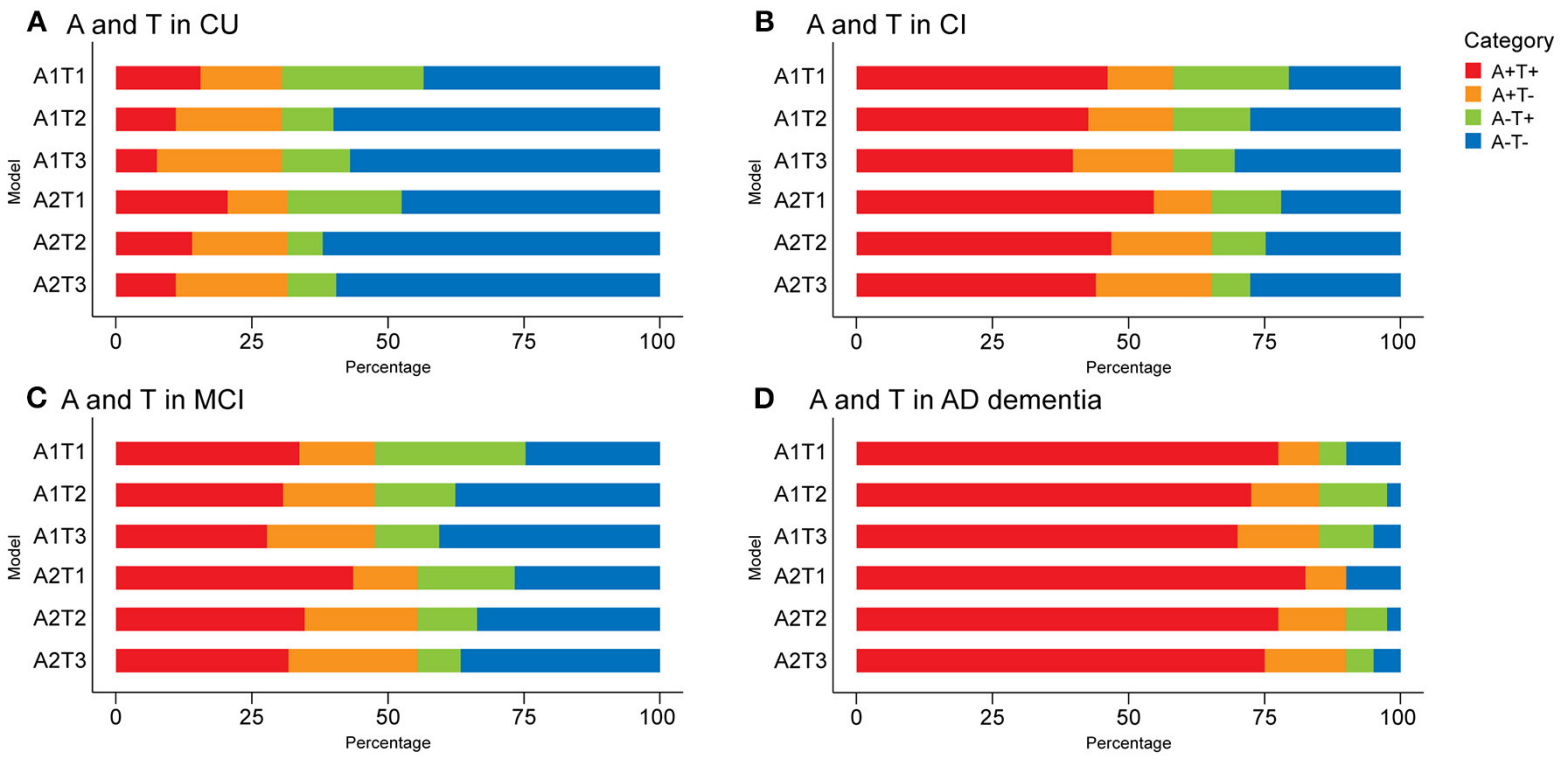
Prevalence of different AT(N) categories in different AT variants among cognitively unimpaired (CU) **(A)** and cognitively impaired (CI) **(B)** participants in the ADNI. Mild cognitively impaired (MCI) **(C)** and Alzheimer's disease (AD dementia) **(D)** are two subgroups of the CI group. CSF Aβ42 (A1); amyloid PET whole cerebellum standardized uptake value ratio (SUVR) (A2); CSF tau phosphorylated at Thr181 (T1); tau PET inferior temporal cortex SUVR (T2); tau PET Braak V/VI SUVR (T3). AT(N), β-amyloid, tau, and neurodegeneration classification system.

**Figure 3:**
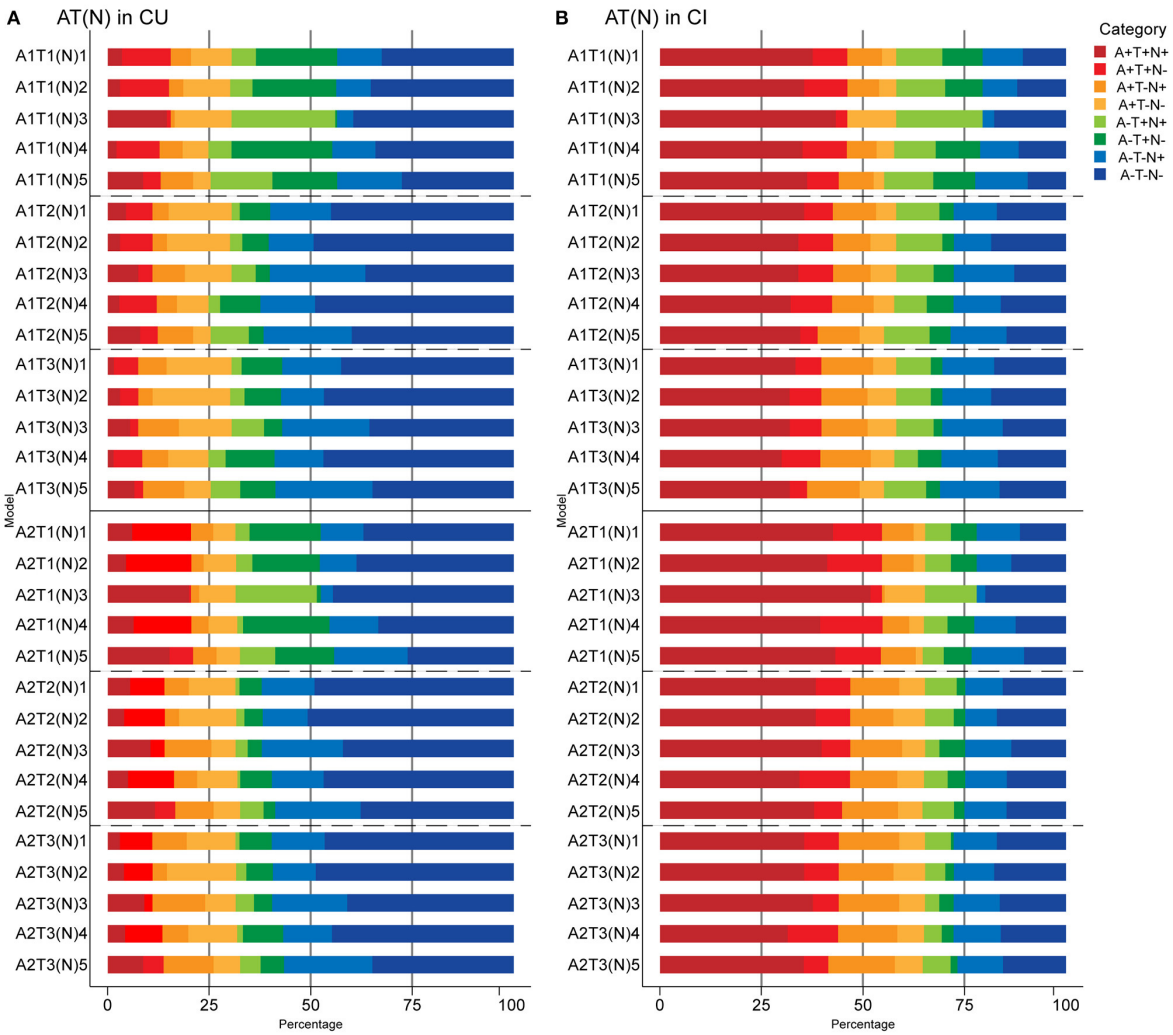
Prevalence of different AT(N) categories in different AT(N) variants among cognitively unimpaired (CU) **(A)** and cognitively impaired (CI) **(B)** participants in the ADNI. CSF Aβ42 (A1); amyloid PET whole cerebellum standardized uptake value ratio (SUVR) (A2); CSF tau phosphorylated at Thr181 (T1); tau PET inferior temporal cortex SUVR (T2); tau PET Braak V/VI SUVR (T3); hippocampal volume [(N)1]; temporal meta-ROI thickness [(N)2]; CSF total tau [(N)3]; FDG PET meta-ROI SUVR [(N)4]; plasma neurofilament light [(N)5]. AT(N), β-amyloid, tau, and neurodegeneration classification system.

The prevalence of AT(N) categories in CU and CI participants is summarized in Figures 2, 3 and Supplementary Tables 4, 5. When only considering A and T in the CU group, A-T- was the most common category (range 43.5% [A1T1; 95% confidence interval, 36.6–50.5%] to 62.0% [A2T2; 95% confidence interval, 55.0–68.8%]). When comparing A biomarkers, slightly more were negative when using CSF Aβ42 instead of amyloid PET (*p* > 0.05). The highest positivity for T was observed when CSF p-tau were used for both A+ or A– (*p* < 0.001) (Figure 2A). Based on these results, the use of CSF p-tau may substantially increase the positive rate of the T component compared to tau PET in CU participants.

When adding (*N*) biomarkers, the most prevalent category was A-T-(N)- (range 26.1% [A2T1(N)5; 95% confidence interval, 18.7–33.3%] to 50.8% [A2T2(N)2; 95% confidence interval, 44.1–58.0%]). Although eight possible categories were identified for each AT(N) variants, A+T+(N)+, A+T-(N)+, and A-T+(N)+ had very low frequencies when using MRI imaging and FDG PET. A+T+(N)-, A+T-(N)+, and A-T+(N)- were almost lacking in the combination of CSF p-tau and t-tau since a strong correlation (ρ = 0.980, *p* < 0.001) and almost perfect agreement (κ=0.876; concordance = 93.8%) was observed between them, as previous study reported (Blennow et al., 1995). Among the different biomarkers for (N), CSF t-tau and plasma NfL were the most prevalent biomarkers resulting in (N)+ cases (*p* < 0.001) (Figure 3A).

### Prevalence Measures in CI Participants

A+T+ was the main category when only A and T biomarkers were used for CI participants (range 39.7% [A1T3; 95% confidence interval, 31.8–48.4%] to 54.6% [A2T1; 95% confidence interval, 45.7–63.5%]). A and T categories of different AT(N) variants in the CI group showed similar trends to the CU [i.e., higher prevalence of A+ using amyloid PET (*p* < 0.05) and lower prevalence of T+ using tau PET (*p* < 0.005)] (Figure 2B). There were significant differences in A and T categories between the two subgroups of CI (Fisher exact test, all *p* < 0.001). In participants with MCI, A-T- were the most common categories when using tau PET in Braak V/VI (Figure 2C). In the AD group, A+T+ accounted for approximately 75% (range 70% [A1T3; 95% confidence interval, 55.8–85.3%] to 82.5% [A2T1; 95% confidence interval, 69.8–93.3%]); the difference from other groups was the lower prevalence of T+ obtained using CSF p-tau than tau PET in the case of A- (*p* > 0.05) (Figure 2D).

When adding (N) biomarkers, the most prevalent category was A+T+(N)+ (range 29.9% [A1T3(N)4; 95% confidence interval, 23.3–38.6%] to 51.8% [A2T1(N)3; 95% confidence interval, 43.2–60.3%]), and the frequencies of T+(N)- and T-(N)+ in the combination of CSF p-tau and t-tau were relatively low (Figure 3B). As mentioned above, A-T-N- was the main category when using tau PET in Braak V/VI combined with some N biomarkers in the MCI group (Supplementary Figure 1A). The AD group had the most A+T+(N)+ (range 60.6% [A1T3(N)5; 95% confidence interval, 44.1–76%] to 80% [A2T1(N)3; 95% confidence interval, 67.6–92.1%]) among the three groups. Again, several categories were lacking or had low frequencies (A-T+N- and A-T-N+ when using tau PET) (Supplementary Figure 1B). The prevalence of all the (N) biomarkers resulting in (N)+ cases was approximative, except it was relatively low when using FDG PET in CI individuals (*p* > 0.05) (Figure 3B).

### Longitudinal Cognition

**Figure 4:**
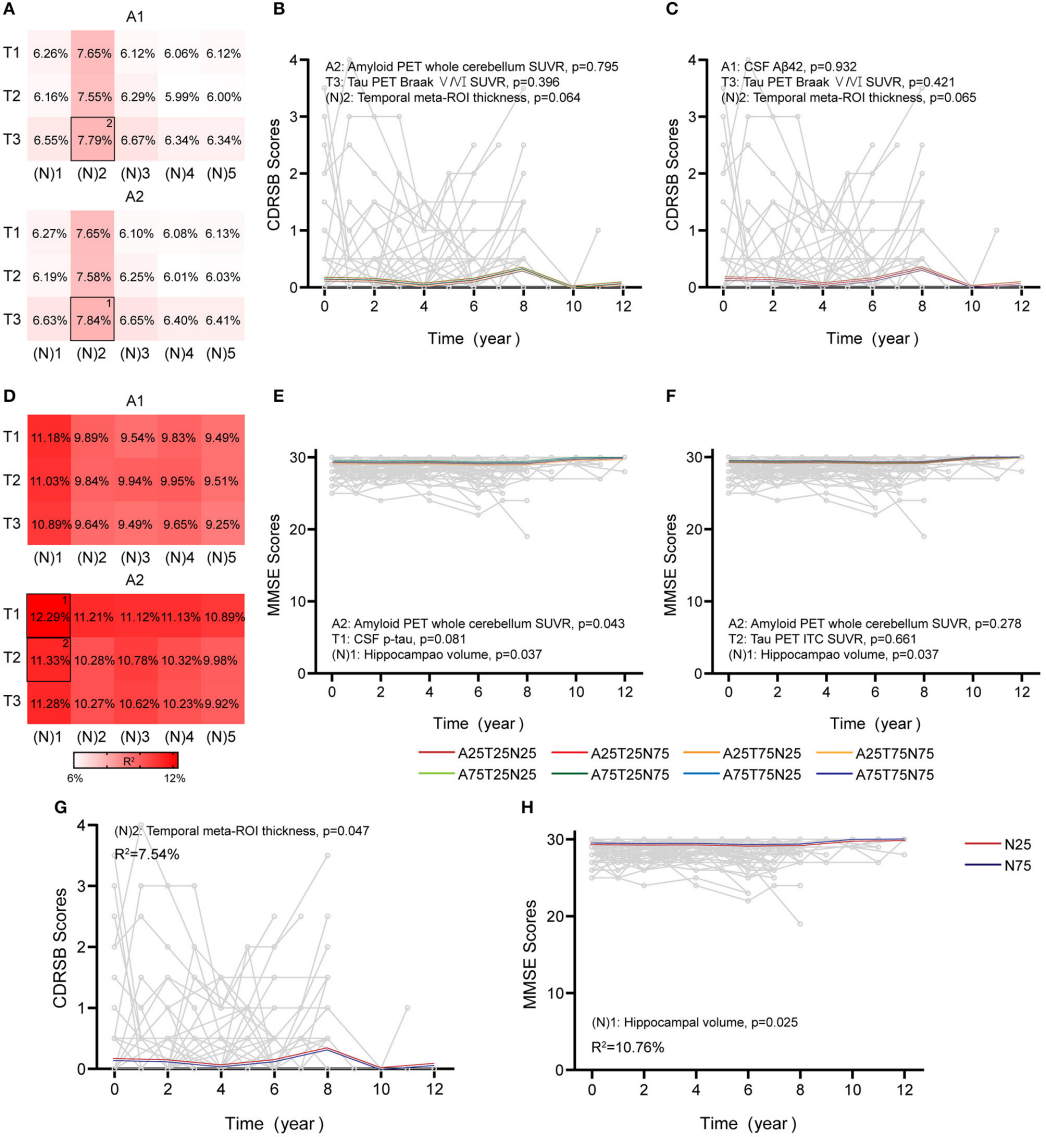
Associations between different AT(N) combinations and longitudinal cognition in the CU group. Marginal R^2^ for different AT(N) variants to predict longitudinal clinical dementia rating sum of boxes (CDRSB) and mini-mental state examination (MMSE) for cognitively unimpaired (CU), respectively (divided by A biomarkers) **(A,D)**. The selected models in **(B,C)** and **(E,F)** are the top two best models for different cognitive scales. The LME models with significant AT(N) biomarkers to predict longitudinal are CDRSB and MMSE, respectively **(G,H)**; AT(N) variants chosen in the model, *p*-values, and marginal R^2^ are shown at the top (CDRSB) or bottom (MMSE) of each panel; 25 and 75 refer to 25th and 75th quartiles, where a lower value indicates a more abnormal biomarker. Aβ, β-amyloid; AT(N), β-amyloid, tau, and neurodegeneration classification system; ITC, inferior temporal cortex; p-tau, tau phosphorylated at Thr181; ROI, region of interest; SUVR, standardized uptake value ratio.

**Figure 5:**
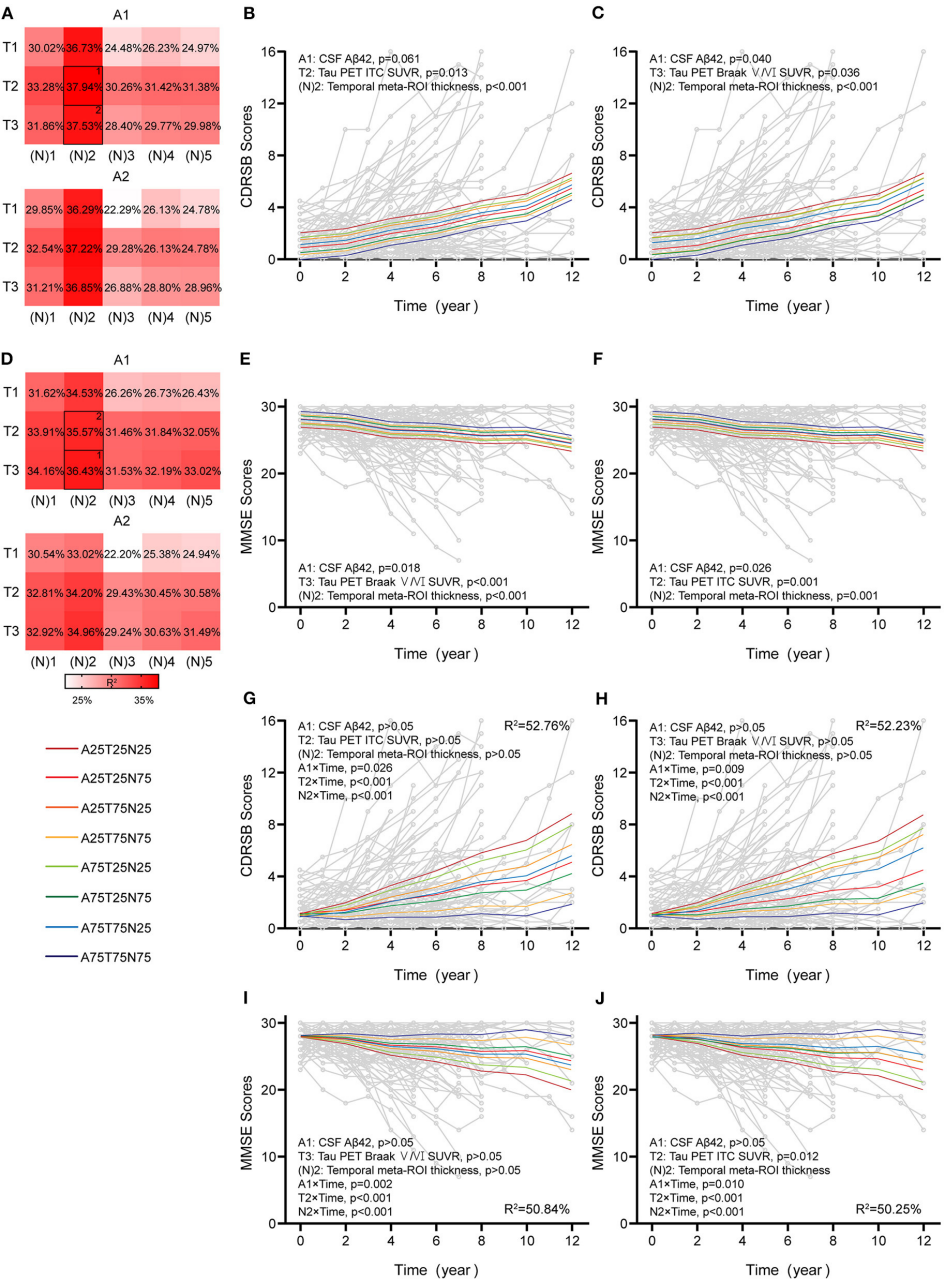
Associations between different AT(N) combinations and longitudinal cognition in the CI group. Marginal R^2^ for different AT(N) variants to predict longitudinal clinical dementia rating sum of boxes (CDRSB) and mini-mental state examination (MMSE) for cognitively impaired (CI), respectively (divided by **A** biomarkers) **(A,D)**. The selected models in **(B,C)** and **(E,F)** are the top two best models for the different cognitive scales. The top two best models with an interaction between time and AT(N) variants to predict longitudinal CDRSB and MMSE, respectively **(G–J)**; AT(N) variants chosen in the model and *p*-values, and marginal R^2^ are shown at the top (CDRSB) or bottom (MMSE) of each panel; 25 and 75 refer to 25th and 75th quartiles, where a lower value indicates a more abnormal biomarker. Aβ, β-amyloid; AT(N), β-amyloid, tau, and neurodegeneration classification system; ITC, inferior temporal cortex; ROI, region of interest; SUVR, standardized uptake value ratio.

The overall findings for longitudinal cognition using continuous predictors are summarized in Figures 4, 5 and Supplementary Tables 6–9. In CU participants, age and education significantly affected cognition (age, *p* = 0.027 and education, *p* = 0.048 in CDRSB; age, *p* = 0.025 and education, *p* < 0.001 in MMSE), consistent with previous findings (Compton et al., 2000; Ardila and Moreno, 2001). When using a single AT(N) biomarker to predict cognitive changes, just the MRI imaging contributed significantly (temporal cortical thickness, [N]2, *p* = 0.047, R^2^ = 7.54% in CDRSB; hippocampal volume, [N]1, *p* = 0.025, R^2^ = 10.76% in MMSE) (Figures 4G,H). The best AT(N) variants capturing changes in cognition in CDRSB and MMSE were A2T3(N)2 (amyloid PET, tau PET in Braak V/VI regions, and temporal cortical thickness) and A2T1(N)1 (amyloid PET, CSF p-tau, and hippocampal volume), respectively, but not all included biomarkers contributed significantly (A2, *p* = 0.795, T3, *p* = 0.396, and [N]2, *p* = 0.064, R^2^ = 7.84% in CDRSB; A2, *p* = 0.043, T1, *p* = 0.081, and [N]1, *p* = 0.037, R^2^ = 12.29% in MMSE) (Figures 4B,E). We considered whether random effects accounted for a greater proportion of the variance because the marginal R^2^ for CU participants was relatively low. Then, we calculated conditional R^2^ using MRI imaging biomarkers (temporal cortical thickness for CDRSB and hippocampal volume for MMSE). After considering the random effect, the conditional R^2^ increased to 19.32% and 33.55% for the CDRSB and MMSE scores, respectively. These results indicated that longitudinal cognition in CU participants was mainly associated with individual characteristics, and MRI measurements were the best biomarkers to predict cognitive changes.

In CI participants, individual characteristics were not significantly associated with cognitive decline. Almost all single AT(N) biomarkers could predict longitudinal cognition, except CSF p-tau (*p* = 0.061) and t-tau (*p* = 0.051) in CDRSB, and the marginal R^2^ using MRI imaging and tau PET was relatively higher than others (Supplementary Table 6). The AT(N) variants combining CSF Aβ42, tau PET, and temporal cortical thickness were the best predictors in both CDRSB and MMSE, and almost all included variables contributed significantly (CDRSB: A1T2[N]2, A1, *p* = 0.061, T2, *p* = 0.013, [N]2, *p* < 0.001, R^2^ = 37.94%; A1T3[N]2, A1, *p* = 0.040, T3, *p* = 0.036, [N]2, *p* < 0.001, R^2^ = 37.53%; MMSE: A1T3[N]2, A1, *p* = 0.018, T3, *p* < 0.001, [N]2, *p* < 0.001, R^2^ = 36.43%; A1T2[N]2, A1, *p* = 0.026, T2, *p* = 0.001, [N]2, *p* = 0.001, R^2^ = 35.57%) (Figures 5B,C,E,F). Then, we found that the interaction between time and AT(N) variants significantly improved the goodness of fit (AIC and BIC) using a paired *t*-test (*p* < 0.001 for CDRSB and MMSE), and interactions dominated the main effects. Again, CSF Aβ42, tau PET, and temporal cortical thickness were the best combinations in both scales, and all interactions were significant (CDRSB: A1T2[N]2, A1, *p* > 0.05, T2, *p* > 0.05, [N]2, *p* > 0.05, A1×Time, *p* = 0.026, T2×Time, *p* < 0.001, [N]2×Time, *p* < 0.001, R^2^ = 52.76%; A1T3[N]2, A1, *p* > 0.05, T3, *p* > 0.05, [N]2, *p* > 0.05, A1×Time, *p* = 0.009, T3×Time, *p* < 0.001, [N]2×Time, *p* < 0.001, R^2^ = 52.23%; MMSE: A1T3[N]2, A1, *p* > 0.05, T3, *p* > 0.05, [N]2, *p* > 0.05, A1×Time, *p* = 0.002, T3×Time, *p* < 0.001, [N]2×Time, *p* < 0.001, R^2^ = 50.84%; A1T2[N]2, A1, *p* > 0.05, T2, *p* > 0.05, [N]2, *p* > 0.05, A1×Time, *p* = 0.010, T2×Time, *p* < 0.001, [N]2×Time, *p* < 0.001, R^2^ = 50.25%) (Figures 5G–J).

Finally, similar findings were observed when using the LME model with time as a covariate to verify the results using continuous predictors (Supplementary Table 9).

### Sensitivity Analyses

We repeated the AT(N) prevalence analyses using alternative cutoffs (Supplementary Table 10). Using cutoffs from 90% sensitivity for AD, except for more amyloid positivity using CSF Aβ42 in CU participants, other results were consistent with the data obtained from the main cutoffs. However, cutoffs defined by the mean ± 2 SD from Aβ-negative CU controls were more conservative. The lowest prevalence of T+ was obtained when using CSF rather than PET, and temporal cortical thickness in all the participants was negative.

## Discussion

In this study, we found that different combinations of AT(N) biomarkers exerted different effects on the category prevalence and predictions of cognitive decline. First, the difference in the composition of AT(N) categories between CU and CI individuals is not surprising. Categories representing the AD continuum were the most common in CI participants, while more subjects with non-AD pathological changes were observed in the CU group (Rami et al., 2011; Jack et al., 2018; Knopman et al., 2018; Carandini et al., 2019). Moreover, different AT(N) variants resulted in considerable differences in prevalence, such as a lower prevalence of T+ when using tau PET in all groups and a higher prevalence of N+ when using fluid biomarkers in the CU group. Finally, different AT(N) combinations have different associations with cognitive changes, with differences observed between CU and CI groups (MRI was more influential in CU participants and tau PET in CI participants). Taken together, these results indicate that different combinations lead to different AT(N) classifications of individuals and different predictions of longitudinal cognition. Our results have important implications for choosing AT(N) combinations according to different needs of research or clinical applications. For instance, we tend to use dynamic fluid examinations for early screening and prevention, and cognition may be predicted by non-invasive MRI imaging in the CU group. Imaging measures that represent the magnitude of the neuropathological load or damage accumulated over time, especially tau PET, may greatly assist with the accurate clinical staging and determination of the prognosis of patients with cognitive impairment.

Biomarkers of AD mainly include fluids and imaging. Here, we chose seven classic biomarkers mentioned in the NIA-AA Research Framework 2018 (Jack et al., 2016, 2018) and plasma NfL, a candidate neurodegeneration marker identified recently (Mattsson et al., 2017a, 2019). However, different biomarkers in the specific AT(N) component may be discordant (Vos et al., 2016; Jack et al., 2018). In our study, the continuous relationship between CSF Ab42 and amyloid PET was “L-shaped” rather than linear (Figure 1A) (Landau et al., 2013; Palmqvist et al., 2015). This may be due to a temporal offset between them (Mattsson et al., 2015; Palmqvist et al., 2016; Vlassenko et al., 2016). In addition, the correlation between CSF p-tau and tau PET was imperfect because p-tau seems to plateau later in the disease (Fagan et al., 2014) whereas the tau PET signal continues to increase (Mattsson et al., 2017b). Among biomarkers in the (N) component, MRI imaging tends to reflect cumulative neuronal loss and shrinkage of the neuropil (Bobinski et al., 2000; Zarow et al., 2005; Barkhof et al., 2007), CSF t-tau, and plasma NfL manifest the intensity of neuronal injury dynamically (van Rossum et al., 2012; Zetterberg, 2016), and FDG PET likely indicates both processes (Alexopoulos et al., 2014). These differences may explain the discordance among (N) biomarkers.

Regarding the AT(N) prevalence, we noted that both AT(N) categories and variants differed between CU and CI participants. Normal AD biomarkers (A-T-[N]-) and non-AD pathological change (A-T+[N]-, A-T+[N]+, and A-T-[N]+) account for most CU individuals, whereas the Alzheimer's continuum (A+T+[N]-, A+T+[N]+, A+T-[N]-, and A+T-[N]+) accounts for CI individuals, especially AD (A+T+[N]-, A+T+[N]+) (Jack et al., 2018). Nevertheless, approximately 1/4 of CU individuals are classified as AD continuum without cognitive symptoms. Cognition is also a continuum and the definition of CU is independent of biomarker findings according to the NIA-AA research framework (Jack et al., 2018). In our study, the overall prevalence of A+ in CU participants was similar, consistent with a metaanalysis (Jansen et al., 2015). However, greater increases in amyloid positivity were observed between the two groups when using amyloid PET. This may be because the CSF analysis detects cerebral Aβ accumulation earlier than PET (Mattsson et al., 2015; Palmqvist et al., 2016; Vlassenko et al., 2016). The same findings were obtained for tau positivity when comparing CSF and PET due to a temporal lag (Mattsson et al., 2017b; McDade and Bateman, 2018). Among the neurodegeneration biomarkers, CSF t-tau and plasma NfL were more common in CU participants, whereas no evident differences were observed in CI participants. These results are consistent with several studies showing that CSF t-tau and blood NfL levels are increased before symptom onset (Mattsson et al., 2017a; Preische et al., 2019).

We repeated prevalence calculations using different cutoffs to verify the prevalence across AT(N) categories and found that the results were not completely consistent. This finding highlights that the optimization of categorization strategies is important for future studies.

Here, we analyzed the predictive effect of different AT(N) variants on longitudinal cognition evaluated using both the CDRSB and MMSE. CDRSB may enable a more detailed analysis of subtle changes with different stages of dementia severity (O'Bryant et al., 2008). First, optimal variants differ by clinical stage. Only MRI measures were significantly associated with cognitive changes in CU participants, whereas the best model for predicting cognition in CI participants included CSF Aβ42, tau PET, and cortical thickness. When using a single AT(N) biomarker for the prediction, no obvious difference was identified between CSF and PET amyloid plaques. This finding may indicate that CSF Aβ42 and amyloid PET can be used interchangeably as several studies have reported (Blennow and Zetterberg, 2018; Hansson et al., 2018). When considering the AT(N) combinations, we found that the amyloid pathology contributed the least to longitudinal cognition in the CI group. This implies that cognitive impairment is weakly related to extracellular Aβ burden and is presumably driven by other factors (Villemagne et al., 2011, 2013; de Wilde et al., 2019), consistent with the characteristics of “A” as state biomarkers (Knopman et al., 2018). However, CSF p-tau is increased earlier in the disease course than tau PET (Blennow and Zetterberg, 2018; La Joie et al., 2018; Mattsson-Carlgren et al., 2020). Therefore, between the two subgroups of CI, the difference in tau PET was more significant than that in CSF p-tau. These results might explain why tau PET far exceeded CSF p-tau levels in the longitudinal prediction of cognition in the CI group. The early tangle pathology identified using tau PET was a better predictor of CDRSB than MMSE, consistent with the characteristics of the scales. Compared to other N biomarkers, MRI measures, especially cortical thickness, were the best. Since hippocampal volume is strongly related to ICV (Jack et al., 2015), different methods for adjusting the volume by ICV associated with sex, age, and study populations may affect study power (Schwarz et al., 2016). A study proposed using thickness measurements, rather than volumes, to assess neurodegeneration in AD cohorts with a large age range (Schwarz et al., 2016). Our results also suggested that cortical thickness may predict cognition more precisely. Among all N biomarkers, the lowest marginal *R*^2^ was obtained when using CSF t-tau to predict longitudinal cognition in CI participants. Firstly, CSF t-tau was reported to be related to multiple variables (age, sex, or education), which may attenuate the association with cognition under adjustment for such covariables (Mielke et al., 2019). Furthermore, recent findings showed that t-tau may be less specific to AD pathology (Buckley et al., 2019; Mielke et al., 2021), and its longitudinal trajectory along the AD continuum is still controversial (Vemuri et al., 2010; Kester et al., 2012; Toledo et al., 2013; Lleo et al., 2019). Similar findings were obtained when considering interactions in CI participants, but the interactions dominated the main effects. Although AT(N) variants were able to predict cognitive changes, their marginal effects relied on the time level. Overall, we obtained relatively robust results for this cohort (MRI for CU participants and the combination of tau PET and cortical thickness for CI participants). Compared to a recent study recruiting participants from Swedish BioFINDER (Mattsson-Carlgren et al., 2020), we confirmed the importance of tau PET in the AD diagnosis and staging, and highlighted that cortical thickness may have a highly significant contribution to cognitive decline.

This study has several limitations. First, the sample size in our study was moderate, which may affect the study power. Especially in the prediction of longitudinal cognition, the sample size of the AD group was too small, which may lead to deviations. So, it limited more refined analysis of subgroups. Secondly, we did not consider the Aβ42/Aβ40 ratio because the Aβ40 of many participants was missing in the database (detailed information was shown in Supplementary Table 2). Additionally, though our research has obtained relatively robust results, it still warrants independent validation in other larger cohorts covering all biomarkers in this study. Furthermore, the greater individual heterogeneity of CU participants may explain the low marginal *R*^2^. Then, differences were observed among different cutoff strategies, and the cutoffs using in the study were sample specific, which may be biased to the sample. Therefore, more approaches for selecting cutoffs or alternatives to binarization (semicontinuous scale; Jack et al., 2016) must be tested. Finally, we only analyzed typical AD biomarkers in this study. With the emergence of an increasing number of biomarkers, they may also need to be included.

Collectively, the proposed AT(N) framework provides a more precise division of the Alzheimer's continuum based on the pathology (Jack et al., 2018), but different biomarkers for defining AT(N) cannot be used interchangeably. Each component of biomarkers included in the AT(N) system classification plays different roles in the stating and staging of AD, and the optimal combinations for predicting cognition may differ by cognitive status. Furthermore, different strategies for discontinuous biomarkers will be an important area for future studies.

## Data Availability Statement

The original contributions presented in the study are included in the article/Supplementary Material, further inquiries can be directed to the corresponding author/s.

## Ethics Statement

The studies involving human participants were reviewed and approved by the Alzheimer's Disease Neuroimaging Initiative (ADNI) study. The patients/participants provided their written informed consent to participate in this study.

## Author Contributions

R-RL: analysis and interpretation of the data and drafting the manuscript. Y-YX, X-YL, and Y-HC: data acquisition, analysis, and interpretation of the data. Q-QT: funding, design of the study, and critical revision of the manuscript. Z-YW: funding, conceptualization and design of the study and critical revision of the manuscript. All authors reviewed the manuscript, contributed to the manuscript revising and editing critically for important intellectual content, given final approval of the version, agreed to be accountable for all aspects of the work presented here, and read and approved the final manuscript.

## Conflict of Interest

The authors declare that the research was conducted in the absence of any commercial or financial relationships that could be construed as a potential conflict of interest.

## Publisher's Note

All claims expressed in this article are solely those of the authors and do not necessarily represent those of their affiliated organizations, or those of the publisher, the editors and the reviewers. Any product that may be evaluated in this article, or claim that may be made by its manufacturer, is not guaranteed or endorsed by the publisher.
