# Supplementary material for: Optimal Combinations of AT(N) Biomarkers to Determine Longitudinal Cognition in the Alzheimer's Disease
Source: Front Aging Neurosci. 2021 Aug 6;13:718959. doi: 10.3389/fnagi.2021.718959 (PMC8377373; doi:10.3389/fnagi.2021.718959)
Supplement: Supplementary file 1 [file Data_Sheet_1.docx]

Supplementary Material

# Supplementary Tables

## Supplementary Table 1. Freesurfer-defined region codes for Braak Ⅴ/Ⅵ ROIs

| Braak Ⅴ | Braak Ⅵ |
| --- | --- |
| 1028 L_superior_frontal  1012 L_lateral_orbitofrontal  1014 L_medial_orbitofrontal  1032 L_frontal_pole  1003 L_caudal_middle_frontal  1027 L_rostral_middle_frontal  1018 L_pars_opercularis  1019 L_pars_orbitalis  1020 L_pars_triangularis  1011 L_lateraloccipital  1031 L_parietalsupramarginal  1008 L_parietalinferior  1030 L_superiortemporal  1029 L_parietalsuperior  1025 L_precuneus  1001 L_bankSuperiorTemporalSulcus  1034 L_tranvtemp  2028 R_superior_frontal  2012 R_lateral_orbitofrontal  2014 R_medial_orbitofrontal  2032 R_frontal_pole  2003 R_caudal_middle_frontal  2027 R_rostral_middle_frontal  2018 R_pars_opercularis  2019 R_pars_orbitalis  2020 R_pars_triangularis  2011 R_lateraloccipital  2031 R_parietalsupramarginal  2008 R_parietalinferior  2030 R_superiortemporal  2029 R_parietalsuperior  2025 R_precuneus  2001 R_bankSuperiorTemporalSulcus  2034 R_tranvtemp | 1021 L_pericalcarine  1022 L_postcentral  1005 L_cuneus  1024 L_precentral  1017 L_paracentral  2021 R_pericalcarine  2022 R_postcentral  2005 R_cuneus  2024 R_precentral  2017 R_paracentra |

## Supplementary Table 2. Characteristic, cognition and AT(N) biomarkers of participants

| RID | ORIGPROT | AGE | GENDER | EDUCATION | APOE4 | DX | CDRSB | MMSE | A1 | A2 | T1 | T2 | T3 | Hip. V | ICV | N1 | N2 | N3 | N4 | N5 | Ab40 |
| --- | --- | --- | --- | --- | --- | --- | --- | --- | --- | --- | --- | --- | --- | --- | --- | --- | --- | --- | --- | --- | --- |
| 31 | ADNI1 | 77.7 | Female | 18 | 0 | CN | 0 | 30 | 784.3 | 1.4584 | 37.33 | 1.9717 | 1.9207 | 7175 | 1311960 | 0.049138 | 2.65225 | 372.7 | 1.16584 | 64 |  |
| 210 | ADNI1 | 72.4 | Female | 13 | 0 | MCI | 0 | 29 | 1271 | 1.2885 | 21.2 | 2.3884 | 2.036 | 6999.7 | 1297855 | -0.09206 | 3.132375 | 267.9 | 1.45604 | 24.7 |  |
| 337 | ADNI1 | 75.8 | Male | 20 | 1 | CN | 0 | 30 | 1422 | 0.9086 | 15.35 | 1.97515 | 1.7771 | 6500 | 1677350 | -1.50933 | 2.806875 | 185.2 | 1.139294 | 70.2 | 15750 |
| 413 | ADNI1 | 76.3 | Female | 16 | 0 | CN | 0 | 29 | 934 | 0.9227 | 10.6 | 1.48585 | 1.3626 | 6671 | 1508702 | -0.93056 | 3.10875 | 116.6 | 1.44821 | 51 |  |
| 416 | ADNI1 | 75.6 | Female | 14 | 0 | CN | 0 | 29 | 1383 | 1.17341 | 21.03 | 2.27985 | 2.0319 | 5690 | 1447820 | -1.76435 | 2.669 | 272.7 | 1.52894 | 100.7 |  |
| 467 | ADNI1 | 81.1 | Female | 12 | 0 | AD | 0 | 30 | 444.1 | 1.416 | 23.38 | 3.36325 | 2.558 | 4415 | 1519700 | -3.21315 | 2.6865 | 241.2 | 1.13324 | 76 |  |
| 498 | ADNI1 | 70.3 | Male | 12 | 1 | CN | 0 | 25 | 1898 | 0.9615 | 28.48 | 2.03955 | 1.9284 | 6866.3 | 1520821 | -0.76456 | 3.061625 | 305.3 | 1.46795 | 27.5 | 23840 |
| 555 | ADNI1 | 77 | Male | 16 | 1 | AD | 0 | 28 | 530.9 | 1.5229 | 21.31 | 2.49205 | 1.8283 | 7491 | 1746500 | -0.68553 | 2.697625 | 217.8 | 1.00169 | 45.3 |  |
| 618 | ADNI1 | 74.8 | Male | 16 | 1 | CN | 0 | 29 | 737.8 | 1.1871 | 28.01 | 2.21015 | 1.9 | 8029 | 1482420 | 0.490987 | 2.9375 | 285.3 | 1.264282 | 23.8 | 18370 |
| 626 | ADNI1 | 83.2 | Male | 16 | 0 | AD | 1 | 25 | 650.3 | 1.4855 | 37.04 | 3.732 | 2.25 | 5094 | 1546580 | -2.59914 | 2.2995 | 373.5 | 1.337 | 128.9 |  |
| 668 | ADNI1 | 73.5 | Male | 12 | 0 | CN | 1.5 | 27 | 1558 | 0.9809 | 16.58 | 2.0278 | 1.8942 | 6978 | 1474920 | -0.54188 | 2.944125 | 194.5 | 1.24766 | 30.8 |  |
| 677 | ADNI1 | 70.8 | Male | 18 | 0 | CN | 0 | 28 | 1622 | 1.03 | 16.45 | 2.2408 | 2.2523 | 7329.3 | 1472090 | -0.18374 | 2.9245 | 180.4 | 1.671182 | 24.9 |  |
| 680 | ADNI1 | 77.8 | Male | 8 | 0 | MCI | 0 | 28 | 2284 | 0.908 | 21.25 | 2.06615 | 1.7646 | 5617 | 1260345 | -1.38406 | 2.766125 | 248.8 | 1.20458 | 32.7 |  |
| 731 | ADNI1 | 71.5 | Male | 18 | 0 | CN | 0 | 30 | 534.8 | 1.6367 | 70.25 | 1.963 | 1.9057 | 7561.6 | 1435999 | 0.135826 | 3.116 | 556.9 | 1.430652 | 107.6 | 27030 |
| 751 | ADNI1 | 70.9 | Male | 16 | 0 | CN | 0 | 27 | 853 | 1.168 | 10.27 | 2.1616 | 1.9282 | 6883 | 1660920 | -1.08661 | 2.92225 | 113.9 | 1.221076 | 62.4 |  |
| 800 | ADNI1 | 74 | Male | 12 | 2 | MCI | 1 | 29 | 521.1 | 1.5505 | 21.19 | 2.84375 | 2.2992 | 5226 | 1466040 | -2.27241 | 2.969875 | 213.9 | 1.250404 | 32.5 |  |
| 896 | ADNI1 | 76.8 | Male | 16 | 0 | MCI | 0 | 30 | 667.6 | 1.1064 | 24.92 | 2.3169 | 2.0845 | 4917 | 1692190 | -3.12821 | 2.288125 | 284.3 | 1.144546 | 44.3 | 18720 |
| 925 | ADNI1 | 74.4 | Male | 12 | 0 | MCI | 2 | 29 | 498.4 | 1.25655 | 15.18 | 1.8645 | 1.8337 | 5805 | 1497770 | -1.77013 | 2.482125 | 159.6 | 1.0829 | 51.3 |  |
| 1016 | ADNI1 | 78.3 | Female | 13 | 0 | CN | 0 | 29 | 658.8 | 1.2069 | 33.08 | 1.98125 | 1.8198 | 6703.3 | 1365614 | -0.55229 | 3.086875 | 314.1 | 1.121146 | 52.2 | 16760 |
| 1052 | ADNI1 | 69 | Female | 8 | 1 | MCI | 1.5 | 26 | 2309 | 0.8801 | 25.56 | 2.51495 | 2.0941 | 5484.9 | 1287006 | -1.58063 | 2.909375 | 287.6 | 1.09862 | 45.3 |  |
| 1169 | ADNI1 | 72.2 | Female | 18 | 0 | MCI | 0 | 30 | 3305 | 0.9133 | 33.26 | 2.4845 | 2.1838 | 5586 | 1551870 | -2.11994 | 2.546 | 345 | 1.25391 | 45.9 | 29820 |
| 1190 | ADNI1 | 76.5 | Female | 13 | 0 | MCI | 0 | 30 | 1045 | 1.21017 | 38.64 | 2.01535 | 1.8898 | 7161 | 1412760 | -0.20858 | 2.899125 | 395.5 | 1.11414 | 57.7 |  |
| 1414 | ADNI1 | 74.3 | Male | 18 | 1 | MCI | 1.5 | 27 | 1106 | 1.2328 | 19.73 | 1.9458 | 1.8643 | 5608 | 1533260 | -2.05294 | 2.5975 | 234.5 | 1.044376 | 25.3 |  |
| 1418 | ADNI1 | 81.5 | Male | 12 | 0 | MCI | 1 | 29 | 1297 | 0.949 | 16.36 | 1.60105 | 1.5519 | 6149.3 | 1526054 | -1.49422 | 2.81625 | 189.4 | 1.220926 | 46.9 |  |
| 2002 | ADNIGO | 64.8 | Male | 16 | 0 | MCI | 2.5 | 28 | 1883 | 1.001 | 15.78 | 1.66905 | 1.7533 | 7422 | 1475290 | -0.09877 | 3.029625 | 140.7 | 1.12038 | 16.4 |  |
| 2018 | ADNIGO | 76.4 | Female | 18 | 0 | CN | 1.5 | 29 | 1925 | 1.0385 | 17.99 | 2.26615 | 2.0815 | 7080.1 | 1442972 | -0.36253 | 2.85975 | 223.2 | 1.39081 | 35.7 |  |
| 2068 | ADNIGO | 83.1 | Male | 20 | 1 | MCI | 1 | 28 | 712.5 | 1.6456 | 35.8 | 2.12215 | 1.8403 | 6728 | 1626040 | -1.15727 | 2.6225 | 348.1 | 1.03575 | 41.5 |  |
| 2119 | ADNIGO | 71.7 | Male | 12 | 0 | MCI | 1 | 29 | 3081 | 1.0065 | 27.02 | 2.07835 | 1.9803 | 6088.4 | 1563685 | -1.6461 | 2.852625 | 354.2 | 1.276088 | 29.4 | 27250 |
| 2121 | ADNIGO | 67.8 | Female | 14 | 1 | MCI | 1 | 23 | 956.3 | 1.3822 | 26.31 | 1.8745 | 1.8012 | 6296.7 | 1487825 | -1.25438 | 3.1 | 291 | 1.34687 | 44.7 |  |
| 2130 | ADNIGO | 76.4 | Male | 16 | 0 | MCI | 0.5 | 28 | 476.6 | 1.6292 | 30.39 | 2.08565 | 1.8255 | 5793.6 | 1707321 | -2.2882 | 2.466375 | 282.6 | 1.052436 | 48.8 |  |
| 2133 | ADNIGO | 62.5 | Female | 18 | 2 | AD | 1.5 | 30 | 470.4 | 1.7113 | 31.22 | 4.518 | 3.24 | 6631 | 1572030 | -1.12368 | 2.81875 | 332.8 | 0.993518 | 25.8 | 17430 |
| 2155 | ADNIGO | 68.9 | Female | 14 | 2 | MCI | 0.5 | 27 | 633 | 1.2575 | 91.4 | 2.37765 | 2.0418 | 6742 | 1422140 | -0.65026 | 3.127 | 795.9 | 1.250122 | 52.8 |  |
| 2183 | ADNIGO | 83 | Male | 18 | 0 | MCI | 1.5 | 28 | 1365 | 0.968489 | 16.72 | 1.8009 | 1.9055 | 7144 | 1499270 | -0.43475 | 3.0085 | 186.5 | 1.227338 | 56 |  |
| 2200 | ADNIGO | 76 | Female | 14 | 0 | MCI | 2 | 26 | 1257 | 0.9428 | 13.35 | 1.54965 | 1.758 | 7743 | 1330550 | 0.57219 | 3.04125 | 151.6 | 1.43683 | 19.9 |  |
| 2219 | ADNIGO | 74.3 | Male | 18 | 0 | MCI | 1 | 29 | 2306 | 1.2685 | 29.38 | 2.9815 | 2.615 | 6311.5 | 1416865 | -1.06801 | 2.581 | 313.8 | 1.26159 | 37.4 |  |
| 2220 | ADNIGO | 76 | Female | 14 | 0 | MCI | 1.5 | 29 | 1257 | 0.894 | 13.35 | 2.00105 | 1.9412 | 7743 | 1330550 | 0.57219 | 3.04125 | 151.6 | 1.43683 | 58.8 |  |
| 2234 | ADNIGO | 63.6 | Female | 16 | 0 | CN | 2.5 | 29 | 2318 | 0.9581 | 20.13 | 1.96445 | 1.942 | 7030.5 | 1220750 | 0.125173 | 3.115125 | 245.3 | 1.452978 | 32.2 | 23730 |
| 2238 | ADNIGO | 68.5 | Male | 18 | 0 | MCI | 1 | 29 | 1779 | 1.0945 | 16.13 | 1.9201 | 2.0331 | 7109.3 | 1552049 | -0.59707 | 3.086875 | 245.3 | 1.627858 | 33.2 |  |
| 2239 | ADNIGO | 73.5 | Female | 15 | 1 | MCI | 1.5 | 28 | 1379 | 1.2289 | 30.88 | 2.3062 | 2.2009 | 7038.6 | 1429154 | -0.37062 | 2.8845 | 352 | 1.324112 | 26.8 |  |
| 2245 | ADNIGO | 57 | Female | 20 | 2 | MCI | 1 | 28 | 1064 | 1.202 | 26.07 | 1.92865 | 1.8364 | 8011.3 | 1253595 | 1.026559 | 2.993125 | 282.3 | 1.361276 | 26.8 | 25090 |
| 2247 | ADNIGO | 65.1 | Male | 19 | 0 | MCI | 1 | 28 | 1403 | 1.0232 | 14.72 | 1.76505 | 1.8581 | 7293 | 1686490 | -0.73843 | 3.064875 | 173.9 | 1.5003 | 36.4 |  |
| 2263 | ADNIGO | 62 | Male | 16 | 1 | CN | 1 | 29 | 892.9 | 1.047 | 15.96 | 1.67715 | 1.6325 | 7288.8 | 1518775 | -0.33712 | 3.032125 | 190.6 | 1.37444 | 43.4 | 15190 |
| 2332 | ADNIGO | 70.5 | Female | 16 | 0 | CN | 1 | 28 | 1767 | 1.0464 | 26.3 | 1.8948 | 1.7284 | 7297 | 1258540 | 0.300302 | 2.8215 | 302.8 | 1.35712 | 33 |  |
| 2333 | ADNIGO | 67 | Male | 12 | 1 | MCI | 0.5 | 28 | 248.1 | 1.4724 | 26.15 | 2.7731 | 2.0157 | 5993.1 | 1403150 | -1.35325 | 2.7855 | 238.9 | 1.254408 | 34.8 |  |
| 2336 | ADNIGO | 72.9 | Female | 12 | 1 | MCI | 1 | 30 | 787.9 | 1.53555 | 35.78 | 3.9374 | 2.7287 | 6470 | 1208220 | -0.40503 | 2.502875 | 350.4 | 1.129314 | 45.6 |  |
| 2367 | ADNIGO | 74.7 | Male | 20 | 1 | MCI | 1.5 | 25 | 1086 | 1.10959 | 18.85 | 1.95125 | 1.8785 | 6658 | 1764780 | -1.56273 | 2.474625 | 220.7 | 1.138744 | 141.8 |  |
| 2373 | ADNIGO | 79 | Female | 13 | 1 | AD | 1 | 29 | 652.6 | 1.2428 | 51.74 | 3.15345 | 2.4736 | 3655.3 | 1197339 | -3.19342 | 2.731625 | 505.9 | 1.196638 | 43.8 | 24530 |
| 2374 | ADNIGO | 81.3 | Female | 18 | 0 | MCI | 0.5 | 26 | 1654 | 0.9322 | 21.78 | 2.2016 | 1.8929 | 5339.8 | 1501824 | -2.24513 | 2.4425 | 281.5 | 1.181892 | 76.5 | 20400 |
| 2380 | ADNIGO | 67.8 | Male | 16 | 2 | MCI | 1.5 | 28 | 696.5 | 1.392 | 21.81 | 3.3056 | 2.4946 | 6249.3 | 1608176 | -1.59278 | 2.546 | 227 | 1.33745 | 42.8 |  |
| 2392 | ADNIGO | 64.4 | Female | 18 | 0 | MCI | 1 | 27 | 728.6 | 1.0361 | 13.04 | 1.84255 | 1.587 | 7219.9 | 1540581 | -0.45874 | 3.214 | 148.1 | 1.259188 | 76.9 |  |
| 2394 | ADNIGO | 67.4 | Male | 20 | 2 | MCI | 0.5 | 30 | 454.7 | 1.18422 | 21.91 | 2.01885 | 1.807 | 7055 | 1569020 | -0.6924 | 3.052625 | 237.6 | 1.438498 | 36.6 | 15070 |
| 2403 | ADNIGO | 79.1 | Female | 15 | 0 | AD | 3.5 | 29 | 508.7 | 1.6019 | 23.44 | 3.18435 | 2.5096 | 4700.1 | 1492955 | -2.86338 | 2.582875 | 255.7 | 1.295128 | 36.6 | 16890 |
| 4003 | ADNI2 | 72.3 | Female | 16 | 1 | CN | 0 | 30 | 886.6 | 1.3277 | 26.49 | 2.0812 | 1.8478 | 7158.3 | 1319625 | 0.013906 | 2.98925 | 270.9 | 1.20178 | 39.7 | 20990 |
| 4021 | ADNI2 | 66.5 | Male | 20 | 0 | CN | 0 | 29 | 418.8 | 0.9183 | 8 | 1.7138 | 1.6236 | 7307 | 1561088 | -0.42122 | 3.012125 | 80.08 | 1.29347 | 12.5 | 5440 |
| 4028 | ADNI2 | 63.5 | Female | 18 | 0 | CN | 0 | 30 | 2250 | 1.05444 | 29.91 | 1.9702 | 1.7966 | 5748 | 1268910 | -1.27377 | 2.678 | 371.5 | 1.32451 | 32.6 | 37480 |
| 4036 | ADNI2 | 74 | Male | 20 | 0 | MCI | 0.5 | 29 | 1842 | 0.9517 | 18.58 | 2.2063 | 1.8963 | 7597.3 | 1407377 | 0.240731 | 3.116125 | 226.7 | 1.46886 | 52.2 |  |
| 4037 | ADNI2 | 75.6 | Male | 16 | 0 | CN | 0 | 27 | 1800 | 0.9935 | 21.09 | 2.0815 | 1.9026 | 7536.5 | 1674120 | -0.46502 | 2.768875 | 232.3 | 1.26315 | 32.5 |  |
| 4043 | ADNI2 | 82.1 | Male | 20 | 0 | CN | 0 | 29 | 1939 | 0.9145 | 21.72 | 2.1019 | 1.8461 | 7605.7 | 1510142 | 0.000659 | 3.067 | 252.4 | 1.503792 | 35.3 |  |
| 4060 | ADNI2 | 84.4 | Male | 18 | 0 | CN | 0 | 30 | 737.5 | 1.3384 | 14.75 | 1.91675 | 1.6038 | 7313 | 1607120 | -0.52652 | 2.887125 | 164.8 | 1.208908 | 77.1 |  |
| 4067 | ADNI2 | 65 | Male | 16 | 0 | MCI | 2 | 29 | 289.5 | 1.5698 | 16.83 | 2.04495 | 1.822 | 7616 | 1477820 | 0.089109 | 2.71075 | 172.8 | 1.226424 | 34.5 | 9480 |
| 4071 | ADNI2 | 84.5 | Male | 13 | 0 | MCI | 0 | 24 | 833.5 | 1.2648 | 43 | 1.9544 | 1.8534 | 6703 | 1653570 | -1.24883 | 2.820625 | 481.5 | 1.23378 | 82.6 |  |
| 4076 | ADNI2 | 72.6 | Female | 20 | 0 | CN | 0 | 30 | 1820 | 1.0396 | 13.75 | 2.2849 | 1.9293 | 6973.5 | 1376490 | -0.30839 | 2.8595 | 169.8 | 1.417724 | 48.5 |  |
| 4084 | ADNI2 | 68.4 | Female | 18 | 0 | CN | 0 | 30 | 2280 | 0.9818 | 20.37 | 2.11185 | 1.8888 | 8118.9 | 1463500 | 0.626633 | 3.2405 | 233.5 | 1.283254 | 19 | 21550 |
| 4090 | ADNI2 | 71.4 | Male | 20 | 0 | CN | 0 | 30 | 1784 | 0.9942 | 19 | 1.65695 | 1.4652 | 7715 | 1503280 | 0.12655 | 2.934125 | 214 | 1.280852 | 25.5 |  |
| 4094 | ADNI2 | 60 | Female | 12 | 0 | AD | 3.5 | 26 | 1210 | 0.9909 | 12.42 | 1.86055 | 1.6571 | 6511.7 | 1151193 | -0.22545 | 3.165125 | 152.6 | 1.26548 | 25.4 |  |
| 4100 | ADNI2 | 78.5 | Female | 12 | 0 | MCI | 0 | 28 | 889.9 | 1.3654 | 28.58 | 1.82035 | 1.8442 | 6135.7 | 1527026 | -1.51017 | 2.95725 | 300.2 | 1.18073 | 57.3 |  |
| 4105 | ADNI2 | 70.8 | Female | 18 | 0 | CN | 0 | 30 | 1033 | 1.0916 | 12.57 | 1.80635 | 1.6381 | 7002.2 | 1403646 | -0.34535 | 3.105375 | 150.4 | 1.31872 | 39.1 |  |
| 4114 | ADNI2 | 55 | Female | 14 | 1 | MCI | 1 | 28 | 916.1 | 1.4257 | 47.82 | 2.25685 | 1.9985 | 7783 | 1456080 | 0.308673 | 2.920875 | 525.1 | 1.444826 | 19.9 |  |
| 4115 | ADNI2 | 67.4 | Male | 18 | 0 | MCI | 1 | 29 | 1887 | 0.9909 | 25.84 | 1.8518 | 1.762 | 8555.8 | 1573316 | 0.798011 | 3.177375 | 294 | 1.327916 | 32.3 | 23450 |
| 4119 | ADNI2 | 79.3 | Male | 20 | 0 | CN | 0 | 30 | 1217 | 1.0569 | 40.95 | 1.9063 | 1.7679 | 7301 | 1558820 | -0.42174 | 3.13375 | 406.5 | 1.308072 | 43.2 | 30870 |
| 4143 | ADNI2 | 63.6 | Female | 19 | 0 | AD | 0.5 | 29 | 2244 | 0.9907 | 20.22 | 2.66485 | 2.166 | 4386.9 | 1287298 | -2.67933 | 2.186125 | 307 | 1.103018 | 43.7 | 22270 |
| 4148 | ADNI2 | 73 | Male | 18 | 0 | CN | 0 | 30 | 1421 | 0.9927 | 13.96 | 1.98 | 1.6631 | 7902.3 | 1478843 | 0.372935 | 2.967625 | 180 | 1.31031 | 43 | 18220 |
| 4149 | ADNI2 | 73.3 | Male | 14 | 1 | AD | 2 | 24 | 732.2 | 1.27303 | 20.3 | 2.07805 | 2.0137 | 6820.1 | 1674594 | -1.18257 | 2.962125 | 221.6 | 1.27504 | 41.6 |  |
| 4160 | ADNI2 | 74.1 | Male | 12 | 0 | MCI | 1 |  | 1219 | 0.9752 | 11.4 | 2.1707 | 1.8248 | 6252.1 | 1553623 | -1.45807 | 2.569 | 131.8 | 1.287028 | 30.4 |  |
| 4162 | ADNI2 | 71.3 | Female | 16 | 2 | AD | 1 | 26 | 647.1 | 1.53249 | 39.25 | 3.0987 | 2.3471 | 5454 | 1422750 | -1.93974 | 2.700375 | 372.6 | 1.16005 | 46.3 |  |
| 4164 | ADNI2 | 72.6 | Male | 12 | 0 | CN | 0 | 30 | 2315 | 0.9975 | 20.96 | 1.6707 | 1.5588 | 6793.8 | 1667955 | -1.19281 | 2.98075 | 239.1 | 1.38728 | 19.8 | 23070 |
| 4169 | ADNI2 | 62 | Female | 13 | 0 | MCI | 0.5 | 28 | 1963 | 1.0721 | 24.91 | 1.91775 | 1.9341 | 7568.8 | 1531456 | -0.08778 | 3.14925 | 290.7 | 1.26958 | 22.8 | 23130 |
| 4175 | ADNI2 | 75.5 | Male | 20 | 1 | MCI | 2.5 | 27 | 857.7 | 1.2959 | 29.2 | 1.72525 | 1.6699 | 7901 | 1478470 | 0.372537 | 2.877625 | 304.3 | 1.249834 | 45.7 |  |
| 4176 | ADNI2 | 84 | Male | 20 | 1 | CN | 0 | 30 | 1099 | 1.375 | 42.84 | 2.0691 | 1.8248 | 6662.4 | 1668533 | -1.32561 | 2.6825 | 417.7 | 1.239846 | 52.1 |  |
| 4177 | ADNI2 | 84.9 | Male | 20 | 0 | CN | 0 | 30 | 869.9 | 1.06834 | 18.96 | 2.51255 | 2.2689 | 8434.3 | 1627887 | 0.544565 | 2.889375 | 214.5 | 1.526912 | 58.6 |  |
| 4179 | ADNI2 | 83 | Male | 20 | 2 | CN | 0 | 30 | 375.4 | 1.6954 | 27.36 | 2.1503 | 1.708 | 6115.1 | 1635695 | -1.79352 | 3.05625 | 300.6 | 1.30489 | 33.9 | 11450 |
| 4187 | ADNI2 | 62 | Male | 16 | 0 | CN | 0.5 | 29 | 1056 | 1.003 | 19.23 | 1.707 | 1.7156 | 8382.5 | 1622852 | 0.504938 | 3.098375 | 221.6 | 1.55516 | 29.1 |  |
| 4188 | ADNI2 | 77.2 | Male | 14 | 1 | AD | 1 | 28 | 567.5 | 1.3742 | 32.42 | 4.12105 | 2.0722 | 4980.5 | 1604043 | -2.85158 | 2.133625 | 341.9 | 1.22594 | 55.7 |  |
| 4197 | ADNI2 | 79.3 | Male | 20 | 1 | MCI | 0.5 | 30 | 600.1 | 1.5883 | 17.78 | 2.4713 | 1.9049 | 3958 | 1571620 | -3.79569 | 2.4795 | 207.3 | 1.28526 | 62.6 |  |
| 4198 | ADNI2 | 78.4 | Female | 16 | 1 | CN | 0 | 30 | 736.3 | 1.63968 | 20.1 | 2.276 | 2.3266 | 6369 | 1365840 | -0.88714 | 2.782375 | 205.5 | 1.241966 | 36.4 |  |
| 4199 | ADNI2 | 59 | Male | 16 | 0 | CN | 2 | 29 | 1559 | 0.9583 | 13.5 | 1.86715 | 1.5924 | 8007.2 | 1453042 | 0.540218 | 2.922375 | 153.6 | 1.37684 | 29.3 |  |
| 4200 | ADNI2 | 70.2 | Female | 18 | 0 | CN | 0 | 30 | 1341 | 1.027 | 39.5 | 2.22365 | 2.023 | 7125.9 | 1455191 | -0.34628 | 3.010625 | 375.7 | 1.44585 | 40.7 | 27990 |
| 4210 | ADNI2 | 63.9 | Male | 18 | 1 | MCI | 0.5 | 29 | 1564 | 1.0173 | 35.86 | 1.5551 | 1.6669 | 8867.9 | 1519121 | 1.241148 | 2.979875 | 360.7 | 1.248746 | 19.4 |  |
| 4213 | ADNI2 | 78 | Female | 14 | 0 | CN | 0 | 28 | 1800 | 0.9349 | 21.08 | 1.61395 | 1.5249 | 7126.9 | 1433039 | -0.29172 | 2.981625 | 239.5 | 1.30538 | 26.5 |  |
| 4224 | ADNI2 | 75.1 | Male | 20 | 0 | CN | 0 | 29 | 1044 | 1.0109 | 15.11 | 2.23045 | 1.9161 | 6310.1 | 1572279 | -1.44518 | 2.9485 | 165.9 | 1.280906 | 52.7 |  |
| 4229 | ADNI2 | 66.4 | Male | 15 | 0 | MCI | 0.5 | 29 | 1042 | 0.9642 | 11.2 | 1.9907 | 1.7604 | 6756.1 | 1568394 | -0.98979 | 2.901375 | 133.2 | 1.33318 | 25.5 |  |
| 4254 | ADNI2 | 85.9 | Female | 12 | 0 | CN | 0 | 26 | 1194 | 1.106 | 31.96 | 2.304 | 2.1141 | 4686 | 1225210 | -2.23011 | 2.42025 | 377.5 | 1.175916 | 54 |  |
| 4262 | ADNI2 | 72.8 | Female | 16 | 0 | MCI | 0 | 29 | 728.8 | 1.5005 | 38.42 | 5.06215 | 2.9549 | 6450 | 1582990 | -1.33118 | 2.655625 | 391 | 1.13103 | 84.3 |  |
| 4271 | ADNI2 | 61.8 | Male | 20 | 0 | MCI | 0.5 | 30 | 1243 | 0.9873 | 19.09 | 3.0518 | 1.834 | 4505.6 | 1355805 | -2.72627 | 2.13625 | 189.8 | 1.2789 | 29.5 |  |
| 4272 | ADNI2 | 70.9 | Male | 12 | 0 | MCI | 1 | 28 | 428.8 | 1.3274 | 22.94 | 1.66365 | 1.4897 | 5225 | 1318110 | -1.91573 | 2.760875 | 242.4 | 1.18802 | 17.3 |  |
| 4275 | ADNI2 | 72.8 | Male | 18 | 0 | CN | 0 | 29 | 1640 | 0.976 | 21.4 | 1.9798 | 1.7186 | 7815.2 | 1583048 | 0.033881 | 2.972375 | 230.1 | 1.26499 | 15.3 |  |
| 4277 | ADNI2 | 71.4 | Female | 18 | 0 | CN | 0 | 28 | 2896 | 1.183 | 20 | 1.96905 | 1.9688 | 5288.8 | 1246444 | -1.67865 | 2.753875 | 236.2 | 1.4879 | 24.8 | 26740 |
| 4278 | ADNI2 | 75 | Male | 14 | 1 | CN | 0 | 27 | 319.3 | 1.7085 | 23.67 | 2.288 | 1.6962 | 5275 | 1568370 | -2.47083 | 2.967875 | 243 | 1.17999 | 28.5 |  |
| 4281 | ADNI2 | 77.5 | Female | 18 | 0 | MCI | 0.5 | 27 | 2420 | 0.948 | 27.46 | 2.005 | 1.9274 | 7043 | 1424790 | -0.35567 | 2.894625 | 325.7 | 1.409634 | 39.6 |  |
| 4290 | ADNI2 | 73.5 | Male | 20 | 1 | MCI | 0 | 29 | 691.1 | 1.2138 | 37.92 | 2.51145 | 2.292 | 5900.1 | 1549077 | -1.79908 | 2.725875 | 389 | 1.086176 | 55 | 20240 |
| 4292 | ADNI2 | 70.9 | Male | 15 | 0 | CN | 0 | 28 | 1837 | 1.0894 | 21.27 | 2.15645 | 1.9797 | 7450 | 1467730 | -0.05249 | 3.024375 | 252.5 | 1.385254 | 26.7 |  |
| 4293 | ADNI2 | 69.7 | Male | 12 | 0 | AD | 2 | 26 | 1795 | 0.9151 | 18.96 | 2.14415 | 1.8338 | 7511.3 | 1483530 | -0.0294 | 3.068375 | 213.3 | 1.16065 | 46.1 |  |
| 4294 | ADNI2 | 75.2 | Female | 16 | 1 | AD | 0 | 28 | 419.1 | 1.3584 | 25.73 | 2.74715 | 2.5733 | 4055 | 1285128 | -3.00599 | 2.812875 | 273 | 1.161152 | 40.4 | 17030 |
| 4299 | ADNI2 | 76.8 | Female | 12 | 0 | MCI | 1 | 25 | 1329 | 0.859 | 19.87 | 1.8948 | 1.7618 | 6148 | 1433940 | -1.27279 | 2.8965 | 248.2 | 1.007796 | 89.4 | 16410 |
| 4301 | ADNI2 | 74.6 | Male | 18 | 0 | MCI | 0.5 | 28 | 777 | 1.0484 | 10.61 | 2.4233 | 2.0742 | 4887.2 | 1606090 | -2.94983 | 2.325875 | 137.5 | 1.25917 | 42.9 | 9780 |
| 4302 | ADNI2 | 76.1 | Male | 20 | 0 | MCI | 1 | 25 | 946.1 | 1.3793 | 46.18 | 3.07 | 2.0965 | 6229 | 1545680 | -1.46197 | 2.832625 | 417 | 1.098032 | 57.8 |  |
| 4313 | ADNI2 | 77 | Female | 18 | 1 | CN | 0 | 25 | 1338 | 1.0101 | 15.58 | 2.25945 | 2.1513 | 7902 | 1494110 | 0.335722 | 2.87075 | 161.5 | 1.257188 | 49.9 |  |
| 4324 | ADNI2 | 62.8 | Female | 14 | 1 | AD | 4.5 | 27 | 222.8 | 1.33493 | 13.68 | 3.93405 | 3.3545 | 4570 | 1342670 | -2.63011 | 2.37625 | 143.3 | 1.11241 | 42.3 |  |
| 4332 | ADNI2 | 69 | Male | 14 | 0 | CN | 0.5 | 28 | 1160 | 1.2657 | 25.86 | 1.76465 | 1.5145 | 7353.8 | 1896880 | -1.18633 | 3.1175 | 277.6 | 1.363206 | 31.4 |  |
| 4335 | ADNI2 | 71.7 | Female | 15 | 0 | CN | 0 | 30 | 203 | 1.3945 | 16.11 | 1.8392 | 1.4982 | 7499.8 | 1310519 | 0.377423 | 2.856125 | 153.9 | 1.356092 | 38 |  |
| 4343 | ADNI2 | 79.6 | Male | 16 | 0 | CN | 0.5 | 30 | 560.7 | 1.4344 | 51.68 | 2.50985 | 2.1949 | 7073.7 | 1474116 | -0.44423 | 2.970125 | 451.5 | 1.30664 | 56.1 | 18260 |
| 4351 | ADNI2 | 67.8 | Female | 16 | 1 | MCI | 0.5 | 29 | 703.7 | 1.35971 | 24.86 | 2.55395 | 2.0202 | 6607.6 | 1392292 | -0.71249 | 2.783625 | 233.7 | 1.43161 | 22.6 |  |
| 4352 | ADNI2 | 83.6 | Male | 16 | 0 | CN | 0 | 29 | 1676 | 1.0571 | 22.8 | 2.2286 | 2.0041 | 5900 | 1575215 | -1.86238 | 3.052 | 254.7 | 1.298392 | 55.7 |  |
| 4356 | ADNI2 | 68.1 | Male | 20 | 0 | MCI | 0.5 | 28 | 3331 | 1.0414 | 52.3 | 1.87735 | 1.8318 | 8538 | 1532630 | 0.878585 | 3.15875 | 553.5 | 1.269548 | 23.3 |  |
| 4365 | ADNI2 | 80.3 | Male | 20 | 0 | MCI | 0 | 29 | 659 | 1.0723 | 17.02 | 2.1116 | 1.9452 | 6840.7 | 1595151 | -0.96988 | 2.789 | 184.1 | 1.14378 | 34 | 16680 |
| 4367 | ADNI2 | 65.1 | Female | 18 | 1 | CN | 0 | 29 | 783.5 | 0.9975 | 21.78 | 1.71905 | 1.6171 | 7441.4 | 1414665 | 0.06721 | 2.91525 | 231.2 | 1.303486 | 27.8 |  |
| 4369 | ADNI2 | 68.3 | Male | 20 | 0 | CN | 0 | 29 | 1816 | 0.9842 | 26.5 | 1.7989 | 1.6975 | 7045.2 | 1329407 | -0.12285 | 3.08875 | 281.2 | 1.2528 | 13.7 |  |
| 4376 | ADNI2 | 76.5 | Female | 16 | 0 | CN | 0 | 29 | 2108 | 0.9604 | 23.97 | 2.1051 | 2.0311 | 6913.1 | 1351985 | -0.30954 | 2.834375 | 277 | 1.333214 | 19.2 |  |
| 4384 | ADNI2 | 62.3 | Male | 18 | 0 | CN | 0 | 30 | 867.5 | 1.0561 | 9.86 | 1.763 | 1.6988 | 7852.2 | 1404276 | 0.50313 | 2.83225 | 109.3 | 1.15025 | 40.1 |  |
| 4387 | ADNI2 | 76.1 | Female | 16 | 1 | CN | 0 | 29 | 1099 | 1.0453 | 21.77 | 2.1218 | 1.9313 | 6305.9 | 1439269 | -1.12778 | 3.17 | 253.2 | 1.288172 | 35.2 |  |
| 4393 | ADNI2 | 73.5 | Male | 20 | 0 | CN | 0 | 30 | 1562 | 1.0676 | 32.99 | 2.0371 | 1.9131 | 7308 | 1772740 | -0.93197 | 3.0055 | 367.6 | 1.274744 | 27.9 |  |
| 4396 | ADNI2 | 78.4 | Female | 12 | 0 | CN | 0 | 30 | 981.6 | 0.952125 | 13.69 | 2.0749 | 1.8988 | 6390.5 | 1176752 | -0.40845 | 2.71325 | 166.5 | 1.26056 | 38.5 |  |
| 4399 | ADNI2 | 77.9 | Female | 16 | 1 | CN | 0 | 28 | 1626 | 1.0633 | 24.54 | 1.94265 | 1.9904 | 7315 | 1412180 | -0.05318 | 3.089 | 277.9 | 1.360884 | 34.5 |  |
| 4401 | ADNI2 | 67.5 | Female | 20 | 1 | CN | 0 | 30 | 1867 | 1.06485 | 32.26 | 1.859 | 1.6709 | 7871 | 1456850 | 0.394812 | 3.077875 | 342.6 | 1.360152 | 40.2 |  |
| 4404 | ADNI2 | 82.5 | Female | 12 | 0 | AD | 1.5 | 29 | 629.8 | 1.5382 | 22.83 | 3.14935 | 2.442 | 5244 | 1211147 | -1.63811 | 2.56675 | 241.3 | 1.128226 | 59.1 |  |
| 4410 | ADNI2 | 69.1 | Female | 14 | 0 | CN | 0 | 29 | 1445 | 1.0486 | 35.06 | 1.96565 | 1.8224 | 5790.9 | 1287659 | -1.2762 | 2.80825 | 351.8 | 1.20749 | 24.3 | 26500 |
| 4414 | ADNI2 | 60.8 | Female | 18 | 1 | AD | 2.5 | 28 | 811.5 | 1.35981 | 47.34 | 9.6019 | 4.1544 | 5333 | 1408190 | -2.02553 | 2.41425 | 495.9 | 1.018382 | 77.2 |  |
| 4415 | ADNI2 | 75.1 | Female | 13 | 2 | AD | 4 | 28 | 406.5 | 2.0179 | 120 | 5.6551 | 3.7879 | 5507 | 1260840 | -1.49526 | 2.89575 | 1018 | 1.06225 | 49 | 18880 |
| 4417 | ADNI2 | 74.4 | Male | 16 | 0 | MCI | 1.5 | 30 | 735.1 | 1.1235 | 22.12 | 1.90395 | 1.7606 | 7203 | 1649285 | -0.73847 | 3.159875 | 254.2 | 1.111598 | 24.7 | 22280 |
| 4422 | ADNI2 | 70.8 | Female | 15 | 0 | MCI | 0.5 | 29 | 803.1 | 1.3327 | 25.07 | 2.90585 | 2.1545 | 6281 | 1320460 | -0.86541 | 2.382875 | 387.8 | 1.12493 | 91.7 |  |
| 4424 | ADNI2 | 66.2 | Female | 12 | 1 | CN | 0 | 26 | 752.9 | 1.08833 | 14.6 | 1.95585 | 1.8428 | 7054.8 | 1200227 | 0.199095 | 3.327875 | 149.6 | 1.330982 | 50.1 |  |
| 4427 | ADNI2 | 71.3 | Male | 12 | 0 | CN | 0 | 30 | 1813 | 0.9682 | 22.5 | 1.79685 | 1.6278 | 7725.5 | 1640521 | -0.19478 | 3.01 | 247.8 | 1.383432 | 41.6 |  |
| 4428 | ADNI2 | 72.6 | Male | 18 | 0 | CN | 0 | 29 | 1454 | 1.0916 | 16.67 | 2.00755 | 1.7207 | 6985.4 | 1615526 | -0.87445 | 2.938625 | 197.6 | 1.185048 | 51.8 |  |
| 4429 | ADNI2 | 77 | Male | 14 | 0 | CN | 0 | 30 | 2396 | 1.1242 | 30.13 | 1.8684 | 2.039 | 7650 | 1599460 | -0.171 | 2.965875 | 319.7 | 1.48003 | 26.3 | 22960 |
| 4430 | ADNI2 | 80 | Male | 15 | 0 | AD | 2.5 | 24 | 674.6 | 1.2742 | 58.64 | 3.9837 | 2.5844 | 3827 | 1440860 | -3.61053 | 1.91675 | 610.6 | 0.943186 | 76.4 |  |
| 4431 | ADNI2 | 74.1 | Male | 16 | 0 | MCI | 0.5 | 28 | 707.5 | 2.2289 | 15.78 | 3.33395 | 2.3023 | 6962.8 | 1715540 | -1.13887 | 2.876125 | 181.7 | 1.294456 | 45.2 | 13760 |
| 4448 | ADNI2 | 63.8 | Female | 16 | 0 | CN | 0 | 29 | 2426 | 1.0302 | 26.82 | 1.93725 | 1.8782 | 7961 | 1422150 | 0.568712 | 3.134875 | 318.8 | 1.283612 | 30 |  |
| 4453 | ADNI2 | 65.9 | Male | 16 | 0 | CN | 0 | 29 | 2040 | 0.9427 | 20.22 | 1.86065 | 1.6091 | 7271.5 | 1534696 | -0.39291 | 2.880875 | 248.7 | 1.276436 | 37.3 |  |
| 4464 | ADNI2 | 70.4 | Male | 16 | 1 | CN | 0 | 30 | 840.8 | 1.05562 | 17.47 | 1.8955 | 1.7141 | 8788 | 1550490 | 1.085401 | 3.069875 | 201.1 | 1.373648 | 35.9 |  |
| 4469 | ADNI2 | 66.1 | Male | 19 | 1 | CN | 0 | 29 | 2952 | 0.9961 | 34.27 | 2.0513 | 1.7798 | 7201.1 | 1417755 | -0.18056 | 3.10475 | 405.3 | 1.40016 | 21.9 | 34100 |
| 4482 | ADNI2 | 77.2 | Female | 16 | 0 | CN | 0 | 28 | 955.8 | 1.5397 | 71.83 | 2.6237 | 1.8957 | 7368 | 1375450 | 0.088627 | 2.742625 | 622.5 | 1.34628 | 50.1 |  |
| 4483 | ADNI2 | 69.5 | Female | 15 | 0 | CN | 0 | 30 | 1696 | 0.9467 | 15.26 | 2.2556 | 1.8242 | 7327.9 | 1337379 | 0.140578 | 2.976375 | 174.3 | 1.418896 | 26 |  |
| 4485 | ADNI2 | 73.3 | Male | 17 | 0 | CN | 0 | 26 | 1563 | 1.06876 | 25.67 | 1.81455 | 1.7033 | 8313 | 1671280 | 0.318346 | 2.807125 | 277.7 | 1.407356 | 35.1 |  |
| 4488 | ADNI2 | 72.6 | Male | 18 | 0 | CN | 0 | 30 | 1620 | 1.0295 | 17.91 | 1.70495 | 1.5474 | 8537.4 | 1632583 | 0.636311 | 3.04675 | 214.2 | 1.33084 | 31.7 |  |
| 4489 | ADNI2 | 74.2 | Male | 20 | 0 | MCI | 1 | 30 | 1146 | 1.19041 | 19.76 | 1.5441 | 1.4069 | 7875 | 1568740 | 0.128275 | 3.3335 | 229.6 | 1.2919 | 52.7 |  |
| 4491 | ADNI2 | 84.1 | Male | 19 | 0 | CN | 0 | 30 | 1640 | 0.8777 | 15.8 | 1.6007 | 1.4344 | 6630.6 | 1441265 | -0.80791 | 2.959625 | 190 | 1.276 | 44.7 |  |
| 4506 | ADNI2 | 71.5 | Male | 19 | 0 | CN | 0.5 | 30 | 964.7 | 0.9478 | 11.33 | 1.88225 | 1.8837 | 8112 | 1464480 | 0.617363 | 2.9415 | 125.9 | 1.15694 | 42.4 |  |
| 4507 | ADNI2 | 78.2 | Male | 16 | 1 | AD | 1.5 | 29 | 570.9 | 1.587 | 38.86 | 3.42405 | 2.0916 | 6387.9 | 1491720 | -1.1726 | 2.6465 | 374.5 | 1.215602 | 36.1 |  |
| 4510 | ADNI2 | 66.4 | Female | 12 | 1 | MCI | 1 | 30 | 802.6 | 1.5373 | 68.07 | 2.4428 | 2.2324 | 6722 | 1488540 | -0.83081 | 3.03625 | 672.4 | 1.17502 | 63.2 |  |
| 4513 | ADNI2 | 60.6 | Male | 19 | 1 | CN | 1 | 29 | 987.5 | 1.12 | 18.89 | 1.8933 | 1.7345 | 6692 | 1361040 | -0.55253 | 2.951 | 230.2 | 1.41505 | 29.5 |  |
| 4520 | ADNI2 | 67.8 | Male | 16 | 0 | CN | 0 | 30 | 1899 | 1.0712 | 43.64 | 1.81885 | 1.6506 | 9164 | 1579870 | 1.390364 | 3.386125 | 485.8 | 1.35018 | 24.6 |  |
| 4521 | ADNI2 | 70 | Male | 18 | 1 | AD | 3 | 27 | 915.2 | 1.6118 | 39.87 | 6.056 | 3.798 | 5586 | 1625880 | -2.29888 | 2.33325 | 444.6 | 1.11231 | 35.3 |  |
| 4536 | ADNI2 | 77.9 | Female | 17 | 0 | MCI | 1 | 28 | 1260 | 1.046 | 27.31 | 1.9572 | 1.77 | 5503.7 | 1428558 | -1.90408 | 2.448875 | 316 | 1.255192 | 29.5 |  |
| 4538 | ADNI2 | 78.8 | Female | 15 | 1 | MCI | 1 | 27 | 766.4 | 1.2469 | 41.59 | 2.56615 | 2.3702 | 5190.9 | 1258579 | -1.80589 | 2.582375 | 432.1 | 1.337284 | 36.6 | 24030 |
| 4547 | ADNI2 | 77.3 | Male | 18 | 0 | AD | 0.5 | 30 | 458.9 | 1.2592 | 23.48 | 1.97135 | 1.6771 | 6711.1 | 1573120 | -1.04621 | 2.92925 | 241.4 | 1.20759 | 45.9 | 15080 |
| 4548 | ADNI2 | 84.8 | Male | 19 | 0 | MCI | 0.5 | 26 | 1162 | 0.898672 | 15.77 | 2.0351 | 1.6925 | 6209 | 1595300 | -1.60194 | 2.542125 | 193 | 1.23478 | 66.2 |  |
| 4552 | ADNI2 | 63.2 | Male | 17 | 1 | CN | 0 | 29 | 1241 | 1.03894 | 19.97 | 1.9195 | 1.7313 | 6140 | 1652780 | -1.80992 | 2.924875 | 231.7 | 1.214604 | 23.3 |  |
| 4556 | ADNI2 | 72.9 | Male | 20 | 0 | CN | 0.5 | 29 | 1184 | 0.961024 | 13.52 | 2.10355 | 1.7586 | 7670.4 | 1497868 | 0.095036 | 3.04725 | 153.5 | 1.51261 | 34.3 |  |
| 4576 | ADNI2 | 70.5 | Female | 14 | 0 | CN | 0 | 30 | 1922 | 1.14817 | 65.53 | 2.10735 | 1.8979 | 7276.3 | 1306154 | 0.164477 | 3.1985 | 700.8 | 1.268798 | 55.6 |  |
| 4578 | ADNI2 | 69.2 | Female | 14 | 0 | CN | 0 | 27 | 1809 | 1.2023 | 15.94 | 2.14715 | 2.2128 | 8875 | 1647840 | 0.937021 | 3.016 | 195.6 | 1.257206 | 33.8 |  |
| 4580 | ADNI2 | 69.7 | Female | 16 | 2 | CN | 0 | 30 | 640.6 | 1.09827 | 25.95 | 2.34075 | 1.9458 | 7023.3 | 1316465 | -0.11345 | 3.051375 | 290.3 | 1.393418 | 55.6 |  |
| 4585 | ADNI2 | 65.3 | Male | 13 | 1 | CN | 0 | 29 | 1541 | 1.02913 | 20.2 | 1.7593 | 1.7486 | 7660.5 | 1440188 | 0.224599 | 2.888125 | 223.3 | 1.163142 | 14.2 |  |
| 4586 | ADNI2 | 75.8 | Male | 20 | 0 | CN | 0 | 30 | 1744 | 0.9949 | 19.26 | 2.06855 | 2.003 | 7423 | 1615440 | -0.43664 | 2.94475 | 211.4 | 1.195464 | 23 |  |
| 4587 | ADNI2 | 65.7 | Female | 16 | 1 | CN | 0 | 29 | 617.5 | 1.26285 | 32.8 | 2.949 | 1.9525 | 7592 | 1344380 | 0.387751 | 2.897625 | 319.2 | 1.323408 | 39.3 |  |
| 4598 | ADNI2 | 65.1 | Female | 20 | 0 | CN | 0 | 30 | 2696 | 1.0476 | 29.77 | 1.6847 | 1.76 | 7267 | 1546970 | -0.42709 | 3.147375 | 348.5 | 1.47799 | 23.2 |  |
| 4604 | ADNI2 | 65 | Male | 19 | 1 | CN | 0 | 29 | 1248 | 1.1118 | 15.8 | 1.63035 | 1.5818 | 7169.5 | 1739278 | -0.98957 | 3.046875 | 181 | 1.33569 | 26.5 | 15380 |
| 4607 | ADNI2 | 56.2 | Female | 20 | 1 | CN | 0 | 29 | 1070 | 0.9925 | 11.33 | 1.6358 | 1.7984 | 7832.6 | 1409645 | 0.470547 | 2.917375 | 128.7 | 1.364088 | 28.7 |  |
| 4630 | ADNI2 | 65.4 | Female | 16 | 0 | MCI | 1.5 | 30 | 1926 | 0.9941 | 17.96 | 1.78445 | 1.8202 | 6790 | 1178140 | -0.0123 | 3.170625 | 204.5 | 1.14745 | 26.9 |  |
| 4631 | ADNI2 | 69.5 | Male | 20 | 0 | AD | 3 | 28 | 629.2 | 1.546 | 34.85 | 2.06585 | 1.7428 | 7942 | 1697040 | -0.11494 | 2.914125 | 310.4 | 1.37242 | 26.2 |  |
| 4637 | ADNI2 | 70.8 | Female | 18 | 0 | CN | 0 | 29 | 357.1 | 1.1242 | 24.47 | 3.6306 | 2.2113 | 6321.8 | 1351201 | -0.89894 | 2.992375 | 238.5 | 1.32353 | 37.2 | 11370 |
| 4643 | ADNI2 | 65.1 | Female | 16 | 0 | CN | 0 | 29 | 2157 | 0.985 | 18.75 | 2.09055 | 1.8297 | 7272 | 1526520 | -0.37264 | 3.032625 | 220.2 | 1.37949 | 19.4 |  |
| 4644 | ADNI2 | 67.6 | Female | 14 | 1 | CN | 0 | 30 | 1778 | 1.0326 | 17.36 | 1.7839 | 1.6901 | 7461.1 | 1380540 | 0.16942 | 2.9885 | 185 | 1.54661 | 15 |  |
| 4654 | ADNI2 | 75.4 | Female | 18 | 0 | MCI | 0.5 | 29 | 1433 | 1.2697 | 40.45 | 2.5553 | 2.2136 | 5920.2 | 1556531 | -1.79701 | 2.577625 | 454.3 | 1.149232 | 38.2 | 29110 |
| 4659 | ADNI2 | 85.9 | Male | 17 | 2 | AD | 1.5 | 28 | 667.9 | 1.5487 | 27.55 | 2.1478 | 1.9371 | 5979.5 | 1582106 | -1.79954 | 2.68025 | 261.8 | 1.185918 | 44.6 |  |
| 4674 | ADNI2 | 77.9 | Male | 20 | 1 | MCI | 0.5 | 24 | 843.1 | 1.4587 | 31.89 | 1.59315 | 1.517 | 8408.8 | 1537860 | 0.736738 | 3.4475 | 329.1 | 1.38093 | 16.1 |  |
| 4706 | ADNI2 | 60.7 | Male | 14 | 1 | CN | 2 | 30 | 1829 | 0.9739 | 27.75 | 1.71375 | 1.6372 | 7641 | 1493450 | 0.076317 | 2.914 | 305.5 | 1.28876 | 21.1 |  |
| 4715 | ADNI2 | 56.7 | Female | 18 | 0 | AD | 1.5 | 25 | 920 | 1.2354 | 82.74 | 5.1816 | 6.0315 | 5080 | 1390810 | -2.23651 | 2.567375 | 772.7 | 1.067248 | 37.5 |  |
| 4721 | ADNI2 | 85.4 | Male | 15 | 0 | MCI | 2.5 | 28 | 746.4 | 1.3321 | 15.52 | 2.14025 | 2.0453 | 6532.1 | 1568100 | -1.21308 | 2.610625 | 157.3 | 0.976121 | 38.9 |  |
| 4722 | ADNI2 | 68 | Female | 18 | 0 | MCI | 1.5 | 28 | 2713 | 1.0787 | 18.55 | 1.96975 | 1.9162 | 7735.3 | 1314751 | 0.602689 | 3.01425 | 227.6 | 1.53268 | 14 |  |
| 4723 | ADNI2 | 69.4 | Female | 18 | 0 | MCI | 1 | 30 | 2499 | 1.0176 | 25.18 | 1.97515 | 1.8594 | 7251.1 | 1333084 | 0.074163 | 2.953625 | 252.8 | 1.48822 | 19.3 |  |
| 4742 | ADNI2 | 70.6 | Female | 14 | 0 | MCI | 1.5 | 30 | 886.3 | 1.06476 | 8.74 | 1.67615 | 1.5458 | 7378.6 | 1550510 | -0.32405 | 2.9855 | 100.2 | 1.31793 | 33.6 |  |
| 4744 | ADNI2 | 72.3 | Female | 12 | 2 | MCI | 0.5 | 24 | 617.8 | 1.2034 | 57.71 | 2.20455 | 1.9004 | 6117 | 1110710 | -0.52226 | 2.965625 | 551.6 | 1.25786 | 26.7 |  |
| 4765 | ADNI2 | 76 | Male | 16 | 1 | AD | 1.5 | 26 | 632.7 | 1.3264 | 46.39 | 2.0365 | 1.8041 | 6121.7 | 1438367 | -1.3098 | 2.338 | 486.6 | 0.996004 | 41.3 |  |
| 4767 | ADNI2 | 66.4 | Female | 18 | 1 | CN | 3 | 30 | 699.9 | 1.131 | 19.73 | 1.9271 | 1.6775 | 5886.1 | 1410744 | -1.47861 | 3.023625 | 204.1 | 1.474426 | 46 | 14260 |
| 4782 | ADNI2 | 71.9 | Male | 16 | 2 | MCI | 1 | 28 | 348.6 | 1.134 | 22.46 | 1.85075 | 1.603 | 5355 | 1490641 | -2.20289 | 2.908875 | 236.6 | 1.051568 | 30.9 | 13140 |
| 4785 | ADNI2 | 66.7 | Female | 13 | 0 | CN | 0 | 29 | 1661 | 0.9614 | 14.04 | 1.8806 | 1.6786 | 6449 | 1494140 | -1.11735 | 2.93225 | 168.1 | 1.362276 | 18.8 |  |
| 4799 | ADNI2 | 68 | Male | 14 | 0 | CN | 0.5 | 29 | 611.3 | 1.3572 | 22.94 | 1.987 | 1.7806 | 7915.6 | 1623552 | 0.036346 | 3.225125 | 232.5 | 1.32791 | 30.5 |  |
| 4809 | ADNI2 | 78.3 | Male | 12 | 1 | MCI | 1 | 24 | 782.6 | 1.31061 | 26.79 | 2.3545 | 1.9762 | 7176 | 1679190 | -0.83778 | 2.627125 | 274.7 | 1.268196 | 42.4 |  |
| 4815 | ADNI2 | 69.8 | Female | 12 | 2 | AD | 3 | 24 | 661.1 | 1.60609 | 55.44 | 2.4314 | 2.176 | 4951 | 1311880 | -2.17467 | 2.838375 | 506.5 | 1.126188 | 39.9 |  |
| 4817 | ADNI2 | 60.7 | Male | 20 | 2 | MCI | 1 | 30 | 600.2 | 1.144 | 11.94 | 1.90175 | 1.8516 | 6295.7 | 1520118 | -1.33346 | 3.008875 | 126.3 | 1.17335 | 15.6 |  |
| 4835 | ADNI2 | 79.3 | Male | 16 | 0 | CN | 0 | 26 | 733.9 | 1.2245 | 34.88 | 1.7098 | 1.7519 | 8808.6 | 1684686 | 0.781532 | 2.896375 | 325.9 | 1.28591 | 37.2 | 20990 |
| 4842 | ADNI2 | 73 | Female | 12 | 0 | CN | 3.5 | 28 | 1197 | 1.0533 | 10.02 | 2.0322 | 1.9011 | 7649 | 1342240 | 0.449925 | 2.971 | 119.5 | 1.197184 | 26.4 |  |
| 4856 | ADNI2 | 65 | Female | 18 | 0 | CN | 0 | 30 | 975.8 | 0.937777 | 10.01 | 2.02075 | 1.8038 | 7917.7 | 1459144 | 0.435965 | 3.2985 | 119.7 | 1.139188 | 25.2 |  |
| 4858 | ADNI2 | 55 | Male | 16 | 2 | MCI | 2 | 30 | 662.4 | 1.20539 | 42.25 | 1.4749 | 1.5232 | 9178 | 1463950 | 1.684645 | 3.08025 | 426.4 | 1.295772 | 37 |  |
| 4862 | ADNI2 | 77.3 | Male | 18 | 0 | AD | 0.5 | 29 | 711.3 | 1.5004 | 46.03 | 2.65155 | 2.1145 | 6306.9 | 1554147 | -1.40454 | 2.853875 | 430.6 | 1.21141 | 39.3 |  |
| 4868 | ADNI2 | 77.1 | Male | 12 | 2 | MCI | 1 | 27 | 519.7 | 1.4795 | 30.84 | 3.12505 | 2.5162 | 5320 | 1543310 | -2.36524 | 2.58825 | 304.8 | 1.08074 | 56 |  |
| 4869 | ADNI2 | 77.2 | Male | 19 | 0 | MCI | 1.5 | 29 | 1875 | 1.0515 | 17.17 | 1.9476 | 1.8725 | 7183.5 | 1476402 | -0.33996 | 2.891 | 225.1 | 1.25528 | 33.5 |  |
| 4874 | ADNI2 | 57.7 | Female | 18 | 1 | CN | 0.5 | 29 | 2314 | 1.0365 | 18.6 | 1.85755 | 1.7174 | 8169.3 | 1336420 | 0.984296 | 3.044875 | 229.9 | 1.37947 | 25.6 |  |
| 4889 | ADNI2 | 75.6 | Male | 18 | 0 | MCI | 2 | 28 | 1213 | 1.0304 | 10.15 | 2.19825 | 2.062 | 7495 | 1831170 | -0.88625 | 3.036375 | 126.3 | 1.363222 | 49.7 |  |
| 4891 | ADNI2 | 59.7 | Male | 15 | 2 | AD | 0.5 | 29 | 533.5 | 1.6515 | 25.35 | 4.1493 | 3.2435 | 7612.4 | 1508408 | 0.01155 | 2.99125 | 234 | 1.10146 | 37 |  |
| 4893 | ADNI2 | 68.1 | Female | 12 | 1 | AD | 4 | 28 | 744.7 | 1.3661 | 42.06 | 3.50765 | 2.2813 | 5942.9 | 1397775 | -1.39045 | 2.791875 | 438.1 | 1.10257 | 63.8 |  |
| 4896 | ADNI2 | 68.1 | Male | 12 | 1 | MCI | 1 | 30 | 2198 | 0.9248 | 21.23 | 1.9497 | 1.6991 | 7214.6 | 1601851 | -0.61218 | 3.038625 | 239.6 | 1.33918 | 33.6 |  |
| 4900 | ADNI2 | 59.8 | Female | 18 | 0 | CN | 0 | 30 | 1605 | 1.0694 | 14.92 | 2.02165 | 1.8332 | 7163.2 | 1251938 | 0.182463 | 3.24275 | 172.5 | 1.57945 | 24.2 |  |
| 4902 | ADNI2 | 75.3 | Female | 15 | 1 | AD | 1.5 | 25 | 818.6 | 1.243 | 29.74 | 6.0146 | 3.0388 | 4663 | 1423330 | -2.73214 | 2.42375 | 333.4 | 0.926544 | 52.1 |  |
| 4919 | ADNI2 | 74.8 | Female | 19 | 0 | MCI | 1.5 | 29 | 902.8 | 1.02522 | 17.27 | 2.1945 | 1.8958 | 6030.3 | 1363543 | -1.22028 | 2.71975 | 208.1 | 1.38173 | 28.5 |  |
| 4926 | ADNI2 | 62.5 | Male | 18 | 1 | MCI | 2 | 29 | 799.5 | 0.948915 | 10.46 | 1.87715 | 1.7712 | 8497 | 1555250 | 0.782892 | 3.2185 | 115.4 | 1.36077 | 26.3 |  |
| 4949 | ADNI2 | 78.1 | Female | 18 | 2 | AD | 4 | 25 | 506.1 | 1.33984 | 36.03 | 2.26685 | 2.0426 | 5721 | 1412440 | -1.64781 | 2.582375 | 382.6 | 1.14224 | 45.3 |  |
| 4951 | ADNI2 | 71.9 | Female | 14 | 1 | CN | 0 | 29 | 1024 | 1.041 | 12.12 | 1.9416 | 1.9128 | 7407.3 | 1364322 | 0.154834 | 3.103375 | 119.4 | 1.216254 | 16.3 |  |
| 4952 | ADNI2 | 69.3 | Female | 12 | 1 | CN | 0 | 28 | 2429 | 0.9854 | 17.18 | 2.04705 | 1.9116 | 7749.6 | 1262471 | 0.743397 | 2.870875 | 215.5 | 1.3522 | 23.5 |  |
| 4974 | ADNI2 | 73.4 | Male | 19 | 0 | AD | 2 | 29 | 710.1 | 1.3692 | 92.67 | 5.45835 | 3.0286 | 6207.8 | 1697872 | -1.85115 | 2.581125 | 788.3 | 0.938051 | 37.5 |  |
| 5004 | ADNI2 | 64.6 | Male | 20 | 0 | MCI | 1.5 | 29 | 1762 | 0.9865 | 21.92 | 2.0179 | 1.7374 | 8445.7 | 1658445 | 0.48208 | 3.151375 | 253.6 | 1.345834 | 23.8 |  |
| 5057 | ADNI2 | 75.2 | Male | 16 | 0 | AD | 1 | 26 | 1283 | 1.0608 | 10.77 | 2.68545 | 2.2162 | 5482.7 | 1532406 | -2.17617 | 2.28925 | 133.3 | 1.06077 | 52 |  |
| 5078 | ADNI2 | 67.4 | Female | 16 | 0 | CN | 0 | 30 | 1341 | 1.0074 | 12.76 | 1.7798 | 1.6335 | 6449.2 | 1439743 | -0.98562 | 3.0355 | 163.3 | 1.340374 | 38.8 | 27990 |
| 5079 | ADNI2 | 73.2 | Male | 18 | 0 | CN | 0.5 | 29 | 2667 | 0.9391 | 21.78 | 2.0015 | 2.0227 | 7578.6 | 1537397 | -0.09234 | 3.0345 | 259.7 | 1.491412 | 62.6 |  |
| 5083 | ADNI2 | 73.7 | Male | 16 | 2 | CN | 0 | 28 | 874.5 | 1.135 | 14.34 | 2.03625 | 1.9517 | 8435.7 | 1312203 | 1.30925 | 2.954625 | 183.3 | 1.4761 | 32.9 |  |
| 5093 | ADNI2 | 69.3 | Female | 19 | 0 | CN | 0.5 | 30 | 2357 | 1.0135 | 23.06 | 1.7747 | 1.8659 | 6206.8 | 1436684 | -1.22063 | 2.938875 | 259.8 | 1.48636 | 23.5 |  |
| 5100 | ADNI2 | 71.2 | Male | 16 | 0 | CN | 0 | 30 | 1536 | 1.03319 | 12.93 | 1.7249 | 1.5239 | 8188.1 | 1704225 | 0.113789 | 2.934 | 146.6 | 1.218504 | 35.8 |  |
| 5109 | ADNI2 | 78.4 | Male | 14 | 0 | CN | 0 | 27 | 775.7 | 1.5044 | 19.58 | 2.2284 | 2.0904 | 6645 | 1385150 | -0.65783 | 2.801625 | 232.8 | 1.25431 | 68.9 |  |
| 5113 | ADNI2 | 65.1 | Male | 16 | 0 | CN | 0 | 30 | 1775 | 0.9831 | 18.7 | 1.8788 | 1.8849 | 8643.6 | 1611712 | 0.792974 | 2.927375 | 220.8 | 1.54289 | 20.4 |  |
| 5118 | ADNI2 | 68.2 | Female | 20 | 0 | CN | 0.5 | 30 | 1210 | 0.988545 | 12.16 | 1.6289 | 1.6063 | 7912.1 | 1306959 | 0.79833 | 3.128 | 150.7 | 1.481522 | 27.4 |  |
| 5124 | ADNI2 | 76.7 | Female | 20 | 0 | CN | 0 | 26 | 2705 | 1.12992 | 30.81 | 2.00845 | 1.7789 | 4265 | 1498090 | -3.3109 | 2.810625 | 340.9 | 1.05979 | 51.2 |  |
| 5127 | ADNI2 | 76.3 | Female | 19 | 1 | CN | 0.5 | 29 | 678.1 | 1.66022 | 24.4 | 2.8458 | 2.1421 | 7418 | 1510080 | -0.18689 | 2.568625 | 249.5 | 1.39039 | 28.7 |  |
| 5140 | ADNI2 | 70.3 | Female | 18 | 0 | CN | 0 | 29 | 791.3 | 1.11454 | 9.41 | 1.86715 | 1.7367 | 8621.6 | 1336122 | 1.437318 | 3.02525 | 127.3 | 1.31427 | 21.4 | 10390 |
| 5154 | ADNI2 | 72.4 | Female | 18 | 1 | CN | 0 | 30 | 1159 | 1.0535 | 15.71 | 1.9122 | 1.7455 | 7405.8 | 1507675 | -0.19328 | 3.144875 | 190.6 | 1.410064 | 19.4 |  |
| 5169 | ADNI2 | 65.7 | Female | 18 | 1 | CN | 0 | 30 | 1490 | 1.09629 | 26.06 | 1.8196 | 1.5535 | 8155.3 | 1376549 | 0.873271 | 3.015625 | 292.9 | 1.42004 | 18.6 |  |
| 5175 | ADNI2 | 79.5 | Female | 20 | 0 | CN | 0 | 30 | 2443 | 0.9878 | 15.99 | 2.05195 | 1.8207 | 7304.3 | 1381202 | 0.011019 | 3.179625 | 211.7 | 1.34161 | 57.8 | 26540 |
| 5176 | ADNI2 | 65.7 | Female | 18 | 1 | CN | 0 | 29 | 867 | 1.0971 | 18.08 | 2.03655 | 1.881 | 6748.8 | 1387623 | -0.56 | 3.093 | 215.8 | 1.206636 | 22.2 |  |
| 5177 | ADNI2 | 72.5 | Female | 18 | 0 | CN | 0 | 29 | 1944 | 1.12459 | 33.07 | 2.09785 | 1.9453 | 7693.1 | 1284951 | 0.632544 | 2.97825 | 366.3 | 1.22724 | 27.6 |  |
| 5178 | ADNI2 | 68.6 | Male | 18 | 0 | CN | 0 | 27 | 1623 | 1.03276 | 23.9 | 2.15045 | 1.7307 | 7065.9 | 1462292 | -0.42345 | 3.1055 | 266.9 | 1.287 | 37.7 |  |
| 5185 | ADNI2 | 69.9 | Female | 20 | 1 | CN | 0 | 30 | 615.2 | 1.2401 | 57.69 | 2.9013 | 2.3168 | 7123 | 1411949 | -0.24462 | 2.657625 | 511.9 | 1.38927 | 35.6 | 24390 |
| 5193 | ADNI2 | 72.5 | Female | 16 | 0 | CN | 0 | 29 | 982.5 | 0.9857 | 12.43 | 2.0004 | 1.8128 | 6992.7 | 1398291 | -0.3419 | 3.008875 | 148.9 | 1.38033 | 39 |  |
| 5194 | ADNI2 | 65.2 | Female | 18 | 0 | CN | 0 | 28 | 1386 | 1.08734 | 11.81 | 2.0211 | 1.8017 | 7889.5 | 1302090 | 0.787503 | 2.990125 | 119.7 | 1.21932 | 27.4 |  |
| 5198 | ADNI2 | 69.2 | Female | 18 | 1 | CN | 0 | 30 | 787.2 | 1.316 | 16.3 | 2.18455 | 1.8658 | 8339.8 | 1514753 | 0.72361 | 2.982 | 193.1 | 1.424406 | 20.7 | 14990 |
| 5200 | ADNI2 | 76.4 | Female | 18 | 0 | CN | 0 | 30 | 1327 | 0.9117 | 9.8 | 1.87835 | 1.6732 | 8122.5 | 1488157 | 0.570616 | 2.716875 | 129.9 | 1.28994 | 43.4 |  |
| 5202 | ADNI2 | 65.7 | Female | 16 | 1 | CN | 0 | 30 | 526 | 1.46777 | 23.48 | 2.1646 | 2.349 | 8122 | 1610690 | 0.273845 | 3.068375 | 233.8 | 1.26962 | 48.2 |  |
| 5203 | ADNI2 | 66.5 | Female | 19 | 0 | CN | 0 | 30 | 1260 | 1.09708 | 18.18 | 1.8684 | 1.7719 | 8122 | 1610690 | 0.273845 | 3.068375 | 208.7 | 1.40783 | 43.4 |  |
| 5222 | ADNI2 | 67.5 | Female | 12 | 0 | CN | 0 | 29 | 1739 | 1.0593 | 21.08 | 1.9912 | 1.9741 | 7434 | 1376060 | 0.153152 | 2.975 | 228.1 | 1.30522 | 14.6 |  |
| 5230 | ADNI2 | 73.5 | Female | 19 | 1 | CN | 0 | 29 | 839.6 | 1.5683 | 21.6 | 2.5686 | 2.4242 | 7371.7 | 1491851 | -0.18912 | 3.04825 | 236.3 | 1.38601 | 24.2 |  |
| 5234 | ADNI2 | 78.6 | Female | 15 | 0 | MCI | 0 | 29 | 426.1 | 1.4749 | 11.39 | 2.3711 | 2.0355 | 7577.2 | 1647612 | -0.36023 | 2.989 | 126.1 | 0.973658 | 66.3 |  |
| 5242 | ADNI2 | 67.2 | Male | 20 | 0 | CN | 0 | 29 | 1306 | 0.917943 | 27.06 | 1.7107 | 1.6218 | 8257 | 1661040 | 0.287105 | 3.0765 | 302.5 | 1.35259 | 28.3 |  |
| 5243 | ADNI2 | 71 | Male | 12 | 1 | CN | 0 | 30 | 1528 | 0.9806 | 14.2 | 2.08345 | 1.7052 | 8628.9 | 1374898 | 1.350862 | 3.08925 | 162.2 | 1.33008 | 16.7 |  |
| 5253 | ADNI2 | 68.1 | Male | 18 | 1 | CN | 0 | 30 | 1514 | 1.04647 | 17.35 | 1.82275 | 1.8934 | 8172.9 | 1619478 | 0.303497 | 2.811875 | 184.5 | 1.36181 | 29.2 |  |
| 5258 | ADNI2 | 77.9 | Female | 12 | 0 | CN | 0.5 | 29 | 945.8 | 1.27727 | 29.42 | 2.3633 | 2.0803 | 6794 | 1213650 | -0.09416 | 2.985875 | 299.7 | 1.34255 | 72 |  |
| 5259 | ADNI2 | 78.8 | Female | 18 | 0 | CN | 0 | 28 | 1347 | 1.03849 | 17.56 | 1.9437 | 2.0429 | 6972.9 | 1376140 | -0.30814 | 2.69525 | 213.6 | 1.31582 | 24.5 |  |
| 5265 | ADNI2 | 75.1 | Female | 18 | 0 | CN | 0 | 30 | 450 | 1.7091 | 33.87 | 2.63785 | 1.8736 | 6610.4 | 1503782 | -0.97926 | 2.973875 | 312.2 | 1.28779 | 37 | 17290 |
| 5266 | ADNI2 | 65.9 | Male | 16 | 0 | CN | 0 | 28 | 3451 | 0.9536 | 25.97 | 2.08345 | 1.7098 | 8150.8 | 1521585 | 0.51809 | 3.093875 | 295.9 | 1.31821 | 34.9 | 30750 |
| 5269 | ADNI2 | 64.9 | Male | 16 | 1 | CN | 0 | 29 | 590.4 | 1.04955 | 10.55 | 1.83145 | 1.7046 | 9516 | 1524390 | 1.876508 | 3.100125 | 108.2 | 1.23539 | 32.5 |  |
| 5277 | ADNI2 | 71.8 | Female | 15 | 1 | MCI | 0.5 | 28 | 760.1 | 1.6335 | 36.21 | 2.1243 | 2.1307 | 6456 | 1305420 | -0.65405 | 2.760125 | 341.2 | 1.39657 | 32.9 |  |
| 5278 | ADNI2 | 80.2 | Male | 19 | 1 | CN | 0 | 29 | 1336 | 1.22623 | 31.48 | 2.3389 | 2.154 | 7480 | 1461910 | -0.00842 | 3.174125 | 347.8 | 1.22315 | 36.1 |  |
| 5282 | ADNI2 | 66.9 | Male | 17 | 1 | CN | 0 | 29 | 558.9 | 1.4172 | 15.63 | 1.8174 | 1.7438 | 7851 | 1498720 | 0.273575 | 3.023375 | 170.7 | 1.1358 | 25.7 |  |
| 5288 | ADNI2 | 81.9 | Female | 18 | 0 | CN | 0 | 30 | 2460 | 1.0943 | 26.59 | 2.00855 | 1.9039 | 7211.9 | 1379695 | -0.07774 | 2.94825 | 300.1 | 1.47256 | 45.2 |  |
| 5289 | ADNI2 | 59.7 | Female | 16 | 1 | CN | 0 | 29 | 1887 | 1.1207 | 15.27 | 1.83485 | 1.9082 | 7858 | 1542567 | 0.174558 | 3.090875 | 177.1 | 1.47587 | 25 | 23450 |
| 5290 | ADNI2 | 67 | Female | 12 | 1 | CN | 0 | 29 | 901.8 | 1.7612 | 54.81 | 2.3041 | 1.9072 | 7491.9 | 1385849 | 0.187385 | 3.134375 | 503.9 | 1.45727 | 39.7 | 33640 |
| 5292 | ADNI2 | 74.3 | Female | 13 | 0 | CN | 0 | 30 | 915.4 | 1.46934 | 40.95 | 2.95235 | 2.2812 | 6577.5 | 1258835 | -0.41991 | 2.980375 | 394.9 | 1.42463 | 48.1 |  |
| 6007 | ADNI3 | 76.8 | Female | 19 | 2 | CN | 0 | 29 | 473 | 1.1743 | 31.48 | 2.33805 | 2.2033 | 7192.4 | 1462469 | -0.29737 | 2.949 | 340.7 |  |  | 18510 |
| 6009 | ADNI3 | 67.5 | Male | 16 | 1 | CN | 0 | 29 | 637.8 | 1.0161 | 9.33 | 1.8135 | 1.5365 | 8887.9 | 1522843 | 1.252149 | 3.131 | 117.8 |  |  |  |
| 6014 | ADNI3 | 67.1 | Male | 18 | 0 | CN | 0 | 30 | 1163 | 1.065 | 10.87 | 1.9235 | 1.7516 | 7650.6 | 1911129 | -0.92398 | 3.051625 | 134.5 |  |  |  |
| 6016 | ADNI3 | 65.4 | Female | 12 | 0 | CN | 0 | 29 | 442 | 1.0603 | 8 | 1.65945 | 1.5915 | 8326.7 | 1487857 | 0.77554 | 3.07175 | 89.11 |  |  | 5220 |
| 6025 | ADNI3 | 79.7 | Female | 20 | 0 | CN | 0 | 29 | 993.8 | 1.0258 | 17.95 | 2.06315 | 1.8539 | 6668.3 | 1338090 | -0.52074 | 2.990125 | 209.1 |  |  | 16410 |
| 6030 | ADNI3 | 65.1 | Female | 18 | 1 | CN | 0 | 30 | 740.4 | 1.0893 | 28.89 | 1.7338 | 1.5992 | 7970.6 | 1382973 | 0.673037 | 3.10675 | 330.6 |  |  |  |
| 6031 | ADNI3 | 67.2 | Female | 20 | 2 | CN | 0 | 29 | 846.5 | 1.0253 | 21.99 | 1.784 | 1.6516 | 7340.4 | 1443382 | -0.10322 | 3.036875 | 248 |  |  | 22040 |
| 6038 | ADNI3 | 77.4 | Male | 16 | 0 | CN | 0 | 30 | 3308 | 0.9675 | 19.38 | 2.11115 | 1.8288 | 6190.1 | 1386269 | -1.11543 | 2.738625 | 266.4 |  |  | 28730 |
| 6039 | ADNI3 | 55.9 | Male | 13 | 0 | AD | 2 | 21 | 673.7 | 1.5255 | 31.27 | 4.45045 | 4.1456 | 7097.7 | 1437082 | -0.33069 | 2.712125 | 320.3 | 0.970712 |  |  |
| 6049 | ADNI3 | 72.9 | Female | 18 | 1 | CN | 0 | 28 | 630.7 | 1.2344 | 30.76 | 2.1723 | 1.7954 | 7075.1 | 1424265 | -0.3223 | 3.023875 | 334.6 |  |  | 19760 |
| 6051 | ADNI3 | 66.3 | Female | 13 |  | CN | 0 | 28 | 1099 | 1.0062 | 11.28 | 1.73645 | 1.5785 | 8260.7 | 1268918 | 1.238909 | 3.33275 | 134.6 | 1.2913 |  |  |
| 6053 | ADNI3 | 65.7 | Male | 16 | 0 | CN | 0 | 30 | 1840 | 0.9853 | 14.84 | 1.82935 | 1.6392 | 9257.8 | 1719999 | 1.145348 | 3.19025 | 175.2 |  |  |  |
| 6056 | ADNI3 | 65 | Male | 16 | 0 | MCI | 0.5 | 30 | 1262 | 0.8054 | 12.47 | 1.9242 | 1.7399 | 7496.1 | 1453156 | 0.028843 | 3.079625 | 153 | 1.259212 |  | 13810 |
| 6059 | ADNI3 | 64.9 | Female | 18 | 0 | CN | 0 | 28 | 1675 | 1.0082 | 19.06 | 1.9433 | 1.7368 | 8112.5 | 1528844 | 0.46224 | 3.024 | 220.7 | 1.235792 |  | 21310 |
| 6063 | ADNI3 | 74 | Female | 15 | 1 | CN | 0 | 30 | 865.1 | 1.1521 | 9.6 | 2.2277 | 1.9161 | 6445.6 | 1444734 | -1.00129 | 3.087125 | 120.2 |  |  | 10700 |
| 6067 | ADNI3 | 63.1 | Female | 18 | 0 | CN | 0 | 29 | 1173 | 1.0011 | 14.65 | 1.8485 | 1.5937 | 8322.6 | 1377105 | 1.039225 | 3.231 | 182.6 |  |  | 14180 |
| 6073 | ADNI3 | 62.9 | Male | 17 | 0 | MCI | 1 | 26 | 319 | 1.4954 | 21.13 | 3.89955 | 3.0849 | 7773 | 1777273 | -0.47793 | 2.948625 | 203 | 1.157598 |  | 8370 |
| 6085 | ADNI3 | 55.8 | Female | 16 | 0 | CN | 0 | 30 | 1703 | 1.0386 | 15.07 | 2.10555 | 1.9978 | 9537.5 | 1529580 | 1.88546 | 3.1215 | 201.4 | 1.366084 |  | 22330 |
| 6113 | ADNI3 | 76.2 | Male | 20 | 0 | CN | 0 | 30 | 1130 | 1.0465 | 12.75 | 2.0276 | 1.7251 | 7425 | 1457701 | -0.05325 | 3.125125 | 169.4 |  |  |  |
| 6134 | ADNI3 | 62.8 | Female | 16 | 2 | MCI | 1 | 30 | 502.8 | 1.3282 | 24.55 | 2.4537 | 2.1253 | 7332.8 | 1415083 | -0.0424 | 3.096625 | 233.9 |  |  | 16080 |
| 6146 | ADNI3 | 65.5 | Female | 16 | 0 | CN | 0 | 30 | 1545 | 0.9655 | 18.1 | 1.9521 | 1.789 | 8065.1 | 1240803 | 1.111288 | 3.085625 | 208.4 |  |  | 18510 |
| 6151 | ADNI3 | 65.1 | Male | 18 | 0 | CN | 0 | 28 | 1184 | 1.0882 | 19.17 | 1.9847 | 1.7842 | 8484 | 1616769 | 0.621146 | 3.2965 | 212 |  |  |  |
| 6163 | ADNI3 | 68.2 | Male | 15 | 0 | CN | 0 | 30 | 879.4 | 1.0967 | 20.34 | 1.5424 | 1.5344 | 8929.8 | 1407389 | 1.573203 | 3.146125 | 212.3 |  |  | 17260 |
| 6175 | ADNI3 | 69.3 | Female | 18 | 1 | CN | 0 | 28 | 740.4 | 1.5092 | 40.97 | 2.08185 | 1.7847 | 7340.4 | 1360179 | 0.097951 | 2.9395 | 426.5 |  |  |  |
| 6179 | ADNI3 | 79.4 | Male | 16 | 0 | AD | 4.5 | 21 | 448.9 | 1.1755 | 28.75 | 1.8484 | 1.7943 | 4156.3 | 1315703 | -2.97861 | 2.900625 | 334.9 |  |  | 16210 |
| 6180 | ADNI3 | 86.8 | Male | 16 | 0 | MCI | 1 | 29 | 2047 | 0.9389 | 25.85 | 2.1404 | 1.6505 | 5669 | 1495865 | -1.90152 | 2.761 | 287.7 | 1.18971 |  | 27070 |
| 6185 | ADNI3 | 82.7 | Male | 18 | 0 | CN | 0.5 | 27 | 1065 | 1.0921 | 29.69 | 1.81415 | 1.5993 | 7537.6 | 1634755 | -0.36874 | 3.05475 | 340.9 |  |  | 25180 |
| 6189 | ADNI3 | 69.5 | Female | 15 | 0 | CN | 0 | 30 | 2759 | 0.984 | 26.66 | 1.98605 | 1.8591 | 7758.4 | 1470141 | 0.250076 | 3.23675 | 307.3 |  |  | 28480 |
| 6197 | ADNI3 | 71.3 | Female | 19 | 1 | CN | 0 | 29 | 1133 | 1.0337 | 13.67 | 2.1169 | 1.8513 | 6848.2 | 1372317 | -0.4236 | 2.983125 | 154.3 |  |  |  |
| 6200 | ADNI3 | 72.3 | Male | 18 | 0 | CN | 0 | 30 | 1212 | 0.9825 | 16.4 | 1.7207 | 1.7262 | 7301.6 | 1720000 | -0.81085 | 3.041625 | 190.5 |  |  |  |
| 6212 | ADNI3 | 71.3 | Female | 18 | 0 | CN | 0 | 30 | 1725 | 1.0598 | 17.76 | 1.94145 | 1.7502 | 7015.7 | 1200383 | 0.159619 | 2.99225 | 209.9 |  |  | 19420 |
| 6226 | ADNI3 | 65.4 | Female | 18 | 1 | CN | 0 | 30 | 1335 | 1.0673 | 20.16 | 1.7612 | 1.6398 | 8348.4 | 1474482 | 0.829579 | 3.007625 | 256.9 |  |  |  |
| 6228 | ADNI3 | 75.1 | Female | 14 | 0 | CN | 0 | 30 | 1307 | 0.9406 | 15.17 | 1.7467 | 1.6206 | 6909.4 | 1311323 | -0.21492 | 3.07675 | 176.4 |  |  | 16970 |
| 6231 | ADNI3 | 69.1 | Female | 16 | 1 | AD | 2 | 22 | 259.1 | 1.4462 | 14.31 | 4.10225 | 2.5485 | 6744.7 | 1517249 | -0.87753 | 2.533125 | 159.2 |  |  | 6300 |
| 6251 | ADNI3 | 65.5 | Female | 16 | 2 | CN | 0 | 29 | 714 | 1.4098 | 30.13 | 2.05825 | 1.7742 | 6841.3 | 1202985 | -0.02107 | 3.386 | 322.4 |  |  | 19310 |
| 6256 | ADNI3 | 66.4 | Female | 16 | 1 | CN | 0 | 30 | 1251 | 1.073 | 17.97 | 2.07475 | 1.9808 | 6763.8 | 1400773 | -0.5768 | 3.013375 | 228.3 |  |  |  |
| 6258 | ADNI3 | 79.8 | Male | 20 | 1 | MCI | 0.5 | 29 | 514.1 | 1.5525 | 14.68 | 1.9847 | 1.8334 | 6869.4 | 1735426 | -1.28035 | 2.895875 | 158 | 1.38033 |  |  |
| 6259 | ADNI3 | 71.4 | Male | 14 | 1 | CN | 0 | 29 | 718.5 | 1.015 | 10.05 | 1.48025 | 1.4723 | 7312.5 | 1649308 | -0.62903 | 3.086 | 134.4 |  |  | 12390 |
| 6260 | ADNI3 | 69 | Male | 20 | 0 | CN | 0 | 29 | 1011 | 1.14 | 15.44 | 1.8887 | 1.588 | 7855.3 | 1490950 | 0.296662 | 3.035375 | 181.5 |  |  |  |
| 6264 | ADNI3 | 55.3 | Male | 12 | 2 | AD | 2.5 | 23 | 344 | 1.6081 | 38.78 | 5.4662 | 4.7458 | 5572.6 | 1594056 | -2.23534 | 3.07575 | 327.7 | 1.000082 |  |  |
| 6268 | ADNI3 | 69.6 | Male | 20 | 2 | MCI | 0.5 | 29 | 446.8 | 1.4173 | 24.53 | 2.6954 | 1.9389 | 8053.3 | 1759485 | -0.15462 | 2.91825 | 257.5 | 1.129008 |  |  |
| 6274 | ADNI3 | 76.7 | Male | 18 | 0 | MCI | 1.5 | 30 | 2026 | 0.9599 | 30.84 | 1.78795 | 1.5274 | 7678.4 | 1647480 | -0.25871 | 2.893625 | 322.7 | 1.150292 |  | 22050 |
| 6283 | ADNI3 | 66.7 | Female | 19 | 2 | CN | 0 | 30 | 782.1 | 1.0454 | 21.93 | 1.94995 | 1.6802 | 7661.3 | 1335198 | 0.479252 |  | 253.9 |  |  | 16080 |
| 6287 | ADNI3 | 69.3 | Female | 18 | 0 | CN | 0 | 29 | 1190 | 0.9622 | 13.58 | 1.81095 | 1.6063 | 8285.8 | 1481001 | 0.751217 | 3.224625 | 155.5 |  |  | 14200 |
| 6288 | ADNI3 | 72.6 | Male | 19 | 1 | CN | 0 | 30 | 616.3 | 1.5294 | 39.71 | 1.9405 | 1.8038 | 7501.9 | 1415320 | 0.126126 | 3.009375 | 400.4 |  |  | 19050 |
| 6289 | ADNI3 | 56.5 | Female | 16 | 2 | CN | 0 | 30 | 458.3 | 1.2602 | 20.66 | 1.29935 | 1.2884 | 8232.3 | 1297553 | 1.141272 | 3.167 | 246.9 |  |  |  |
| 6292 | ADNI3 | 76.6 | Male | 20 | 0 | CN | 0 | 27 | 1651 | 0.9494 | 16.47 | 1.83685 | 1.7154 | 6617.6 | 1462672 | -0.87267 | 3.09375 | 203.1 |  |  | 19170 |
| 6293 | ADNI3 | 86.3 | Female | 20 | 0 | CN | 0 | 30 | 3065 | 1.1567 | 40.94 | 1.9955 | 1.8952 | 6881.4 | 1547386 | -0.81369 | 2.9845 | 445.4 |  |  | 30750 |
| 6294 | ADNI3 | 82.4 | Male | 16 | 0 | CN | 0 | 28 | 735.8 | 1.7134 | 49.4 | 1.8887 | 1.8567 | 7217.9 | 1609652 | -0.62774 | 2.973375 | 421.3 |  |  | 21350 |
| 6297 | ADNI3 | 81.4 | Male | 20 | 0 | MCI | 1.5 | 30 | 671.3 | 0.9013 | 17.65 | 2.13075 | 1.8051 | 8253.5 | 1528540 | 0.603973 | 3.09 | 199.5 | 1.32689 |  | 15840 |
| 6303 | ADNI3 | 70.4 | Male | 16 | 0 | AD | 5 | 23 | 689.7 | 1.5954 | 32.74 | 2.51855 | 2.4462 | 5413.2 | 1654085 | -2.53988 | 2.58625 | 319.5 | 0.979487 |  | 19330 |
| 6304 | ADNI3 | 66.3 | Female | 18 | 1 | CN | 0 | 30 | 876.9 | 1.0914 | 12.91 | 1.87435 | 1.7161 | 7224.5 | 1146979 | 0.497542 | 3.010375 | 153.7 |  |  | 13170 |
| 6307 | ADNI3 | 76.3 | Male | 16 | 1 | CN | 0 | 27 | 557.1 | 1.1901 | 14.49 | 2.1363 | 1.8218 | 6970.4 | 1517942 | -0.6535 | 3.132 | 151.3 |  |  | 12170 |
| 6314 | ADNI3 | 73.6 | Male | 16 | 0 | CN | 0 | 30 | 843.6 | 0.9893 | 11.71 | 1.952 | 1.7338 | 6823.5 | 1569329 | -0.92465 | 2.8485 | 145.2 |  |  | 10530 |
| 6316 | ADNI3 | 77.5 | Female | 16 | 1 | CN | 0 | 30 | 572.1 | 1.2247 | 20.81 | 1.64515 | 1.6247 | 8270 | 1455727 | 0.796526 | 3.079875 | 231.4 |  |  | 15610 |
| 6318 | ADNI3 | 69.5 | Female | 14 | 0 | CN | 0 | 29 | 860.5 | 1.3697 | 47.85 | 2.0674 | 2.0039 | 7776.4 | 1461546 | 0.288857 | 3.052625 | 497.5 |  |  | 29920 |
| 6320 | ADNI3 | 65.4 | Female | 18 | 0 | CN | 0 | 30 | 2391 | 1.0107 | 23.49 | 1.6681 | 1.4847 | 7135.5 | 1113179 | 0.490267 | 3.224 | 271 |  |  | 23540 |
| 6334 | ADNI3 | 72.6 | Male | 16 | 0 | MCI | 0.5 | 29 | 1007 | 1.093 | 12.25 | 1.77325 | 1.6512 | 7616.9 | 1627385 | -0.27162 | 3.219375 | 153.4 | 1.30361 |  | 12370 |
| 6346 | ADNI3 | 67 | Male | 16 | 0 | CN | 0 | 27 | 995.5 | 1.0468 | 15.59 | 1.6758 | 1.4752 | 8882.2 | 1676459 | 0.875024 | 3.301 | 185 |  |  | 15610 |
| 6354 | ADNI3 | 70.8 | Female | 18 | 1 | CN | 0 | 30 | 867.8 | 1.0604 | 21.21 | 2.2332 | 2.2181 | 6654.9 | 1278665 | -0.39046 | 2.979125 | 236.2 |  |  | 20410 |
| 6356 | ADNI3 | 68.7 | Male | 19 | 2 | MCI | 1 | 29 | 379.3 | 1.2572 | 18.59 | 1.86035 | 1.6468 | 7825.8 | 1598425 | 0.0073 | 2.552 | 200.2 | 1.192448 |  | 11410 |
| 6367 | ADNI3 | 81.2 | Female | 20 | 0 | CN | 0 | 30 | 1173 | 1.4367 | 33.1 | 1.9644 | 1.937 | 8001.7 | 1507379 | 0.403339 | 3.085625 | 362.9 |  |  | 14180 |
| 6369 | ADNI3 | 76.9 | Male | 18 | 0 | MCI | 0.5 | 29 | 2389 | 0.949 | 23.44 | 1.7902 | 1.7385 | 7583.2 | 1799982 | -0.72264 | 3.171375 | 258.8 |  |  | 24060 |
| 6374 | ADNI3 | 64.2 | Female | 16 | 0 | CN | 0 | 30 | 1634 | 0.9611 | 14.53 | 1.96905 | 1.795 | 7661.2 | 1557489 | -0.05832 | 2.965875 | 166.8 |  |  |  |
| 6396 | ADNI3 | 75.3 | Female | 14 | 0 | CN | 0.5 | 29 | 2579 | 0.971 | 22.51 | 1.88275 | 1.8732 | 7747.8 | 1278222 | 0.703512 | 2.818875 | 263.6 |  |  | 24210 |
| 6399 | ADNI3 | 64 | Female | 12 | 1 | CN | 0 | 28 | 1460 | 1.0972 | 13.62 | 1.8378 | 1.8109 | 8884.3 | 1571633 | 1.13058 | 3.026875 | 158.9 |  |  |  |
| 6400 | ADNI3 | 64.3 | Female | 12 | 1 | CN | 0 | 30 | 2156 | 1.05 | 25.13 | 1.8226 | 1.7398 | 7289 | 1361900 | 0.042388 | 3.2015 | 295.8 |  |  | 23230 |
| 6401 | ADNI3 | 75.7 | Male | 18 | 1 | CN | 0 | 30 | 640.3 | 1.1563 | 32.51 | 1.8324 | 1.6677 | 8291.5 | 1573102 | 0.534227 | 3.033875 | 340.8 |  |  | 24460 |
| 6413 | ADNI3 | 78.2 | Female | 20 | 1 | CN | 0 | 26 | 902.5 | 1.356 | 22.96 | 2.3071 | 1.9752 | 7217 | 1341849 | 0.018871 | 3.008 | 257.2 |  |  | 18500 |
| 6426 | ADNI3 | 79.5 | Female | 14 | 0 | MCI | 0.5 | 27 | 579.7 | 1.5792 | 50.03 | 2.3658 | 2.0292 | 7392.2 | 1332511 | 0.216649 | 2.948 | 438.8 | 1.522102 |  |  |
| 6427 | ADNI3 | 65.3 | Female | 16 | 0 | MCI | 1.5 | 27 | 2085 | 1.0254 | 18.41 | 1.9201 | 1.6961 | 7227.8 | 1291633 | 0.151087 | 2.945375 | 216.4 | 1.286578 |  |  |
| 6429 | ADNI3 | 68.8 | Female | 17 | 0 | CN | 0 | 30 | 872.5 | 0.9034 | 16.04 | 2.0456 | 1.9223 | 7914.3 | 1358087 | 0.67691 | 3.0775 | 170.9 |  |  | 11120 |
| 6432 | ADNI3 | 67.3 | Female | 18 | 2 | MCI | 0.5 | 30 | 507.3 | 1.3362 | 26.87 | 2.19645 | 1.9709 | 6855.4 | 1245365 | -0.10944 | 3.07725 | 264.8 | 1.257478 |  |  |
| 6452 | ADNI3 | 62.3 | Female | 14 | 1 | CN | 0 | 28 | 734.6 | 1.2149 | 26.69 | 1.8305 | 1.6549 | 8179.2 | 1283086 | 1.123153 | 3.123 | 305.6 |  |  |  |
| 6456 | ADNI3 | 86.4 | Male | 20 | 0 | CN | 0 | 30 | 819.7 | 1.1579 | 19.65 | 1.8067 | 1.7203 | 7421.4 | 1595084 | -0.38902 | 3.062875 | 237.3 |  |  | 18490 |
| 6459 | ADNI3 | 66 | Female | 16 | 0 | CN | 0 | 29 | 2901 | 0.999 | 24.94 | 1.77015 | 1.6573 | 8291.7 | 1320971 | 1.14405 | 3.201875 | 304.7 |  |  | 28980 |
| 6467 | ADNI3 | 71.2 | Female | 13 | 0 | MCI | 1 | 27 | 669.8 | 1.3467 | 56.13 | 3.2899 | 2.0345 | 5274.8 | 1454361 | -2.19537 | 2.65375 | 562.6 | 1.443832 |  | 30190 |
| 6476 | ADNI3 | 75.2 | Male | 16 | 0 | CN | 0 | 30 | 917.2 | 0.9827 | 13.11 | 2.11955 | 1.6407 | 7446.8 | 1448512 | -0.00923 | 2.777 | 150.5 |  |  | 15230 |
| 6487 | ADNI3 | 68.3 | Male | 20 | 0 | CN | 0 | 29 | 1076 | 0.9955 | 13.36 | 1.92105 | 1.7782 | 7196.7 | 1487955 | -0.3547 | 3.075375 | 165.5 |  |  |  |
| 6524 | ADNI3 | 66.4 | Female | 12 | 0 | CN | 0 | 30 | 1111 | 0.9585 | 15.48 | 2.0126 | 1.7417 | 7666.6 | 1297328 | 0.576117 | 2.961375 | 171.2 |  |  |  |
| 6564 | ADNI3 | 71.3 | Female | 18 | 2 | CN | 0 | 29 | 1076 | 0.9305 | 16.2 | 2.0911 | 1.9096 | 6215.3 | 1409072 | -1.14537 | 3.118625 | 184.6 |  |  |  |
| 6600 | ADNI3 | 71.2 | Female | 14 | 1 | AD | 5.5 | 17 | 722 | 1.5843 | 61.99 | 3.27905 | 2.553 | 5346.4 | 1299866 | -1.75022 | 2.588375 | 582.9 | 0.954627 |  | 20050 |
| 6602 | ADNI3 | 63.4 | Male | 16 | 2 | AD | 5 | 22 | 495.4 | 1.7182 | 26.38 | 4.8739 | 5.3957 | 5859.7 | 1639822 | -2.05889 | 2.63625 | 272.3 | 0.993174 |  | 9780 |
| 6606 | ADNI3 | 73.6 | Female | 16 | 1 | MCI | 3 | 26 | 515.8 | 1.6459 | 54.53 | 5.384 | 2.938 | 5246.7 | 1280901 | -1.80406 | 2.707875 | 500.8 | 1.482838 |  | 18000 |
| 6629 | ADNI3 | 57 | Female | 14 | 0 | CN | 0 | 30 | 725.4 | 0.9917 | 11.12 | 1.7488 | 1.686 | 7417.2 | 1322861 | 0.26498 | 3.063875 | 125.9 |  |  |  |
| 6632 | ADNI3 | 74.5 | Female | 14 | 1 | MCI | 1.5 | 22 | 411.4 | 1.1894 | 96.93 | 6.0722 | 3.203 | 6810.2 | 1226221 | -0.10836 | 2.69225 | 718.3 | 1.281689 |  |  |
| 6652 | ADNI3 | 86.4 | Female | 20 | 0 | MCI | 2.5 | 29 | 2206 | 1.0647 | 21.98 | 1.80045 | 1.583 | 7115.3 | 1280095 | 0.066484 | 2.983 | 240.2 | 1.343646 |  | 22140 |

Age, CDRSB and MMSE scores shown in the table represent baseline data. DX: diagnosis; A1: CSF Ab42; A2: amyloid PET; T1: CSF p-tau; T2: tau PET in ITC; T3: tau PET in Braak V/VI; Hip. V: bilateral hippocampal volume; ICV: intracranial volume; N1: adjusted hippocampal volume; N2: temporal cortical thickness; N3: CSF t-tau; N4: FDG PET; N5: plasma NfL; Ab40: CSF Ab40.

## Supplementary Table 3 Cutoffs derived from Youden index and 2 alternative strategies

| AT(N) biomarker | Reference | Youden index | 90% sensitivity for AD | mean ± 2 SD from Aβ-negative CU controls |
| --- | --- | --- | --- | --- |
| CSF Aβ42, ng/L | <880 | NA | <920.0 | <492.6 |
| Amyloid PET SUVR | >1.1 | >1.132 | >1.176 | >1.305 |
| CSF p-tau, ng/L | NA | >21.11 | >18.96 | >42.19 |
| Tau PET in ITC SUVR | NA | >2.122 | >2.066 | >2.377 |
| Tau PET in Braak Ⅴ/Ⅵ SUVR | NA | >1.938 | >1.804 | >2.140 |
| Adjusted hippocampal volume, cm^3^ | NA | <-0.4477 | <-0.3307 | <-1.6646 |
| Temporal meta-ROI thickness, mm | NA | <2.9214 | <2.9621 | <1.1252 |
| CSF t-tau, ng/L | >300 | >233.6 | >213.3 | >438.9 |
| FDG-PET meta-ROI SUVR | NA | <1.260 | <1.267 | <1.124 |
| Plasma NfL in younger participants, ng/L | NA | >30.45 | >26.08 | >45.74 |
| Plasma NfL in older participants, ng/L | NA | >36.35 | >37.50 | >68.48 |

## Supplementary Table 4 Prevalence of different AT variants among 4 groups

| AT variants | category | CU | | CI | | MCI | | AD | |
| --- | --- | --- | --- | --- | --- | --- | --- | --- | --- |
|  |  | frequency, % | 95% CI, % | frequency, % | 95% CI, % | frequency, % | 95% CI, % | frequency, % | 95% CI, % |
| A1T1 | A-T- | 43.5 | 36.6-50.5 | 20.6 | 13.9-27.6 | 24.8 | 16.0-33.3 | 10.0 | 2.3-20.0 |
|  | A-T+ | 26.0 | 20.0-32.5 | 21.3 | 14.6-28.5 | 27.7 | 18.6-36.5 | 5.0 | 0.0-12.5 |
|  | A+T- | 15.0 | 10.5-20.0 | 12.1 | 6.9-18.1 | 13.9 | 7.7-20.9 | 7.5 | 0.0-17.1 |
|  | A+T+ | 15.5 | 10.7-20.6 | 46.1 | 36.8-54.5 | 33.7 | 24.8-43.5 | 77.5 | 63.5-90.5 |
| A1T2 | A-T- | 60.0 | 53.1-66.7 | 27.7 | 20.5-36.4 | 37.6 | 28.0-46.7 | 2.5 | 0.0-7.7 |
|  | A-T+ | 9.5 | 5.9-13.8 | 14.2 | 8.8-20.3 | 14.9 | 8.0-21.6 | 12.5 | 2.7-23.3 |
|  | A+T- | 19.5 | 14.1-25.4 | 15.6 | 10.1-21.7 | 16.8 | 9.9-25.2 | 12.5 | 2.6-23.5 |
|  | A+T+ | 11.0 | 6.5-15.9 | 42.6 | 34.3-51.3 | 30.7 | 22.2-40.4 | 72.5 | 57.9-86.5 |
| A1T3 | A-T- | 57.0 | 50.3-63.8 | 30.5 | 22.8-38.6 | 40.6 | 30.4-50.5 | 5.0 | 0.0-12.8 |
|  | A-T+ | 12.5 | 8.2-17.3 | 11.3 | 6.5-16.9 | 11.9 | 5.7-18.3 | 10.0 | 2.2-20.0 |
|  | A+T- | 23.0 | 17.1-29.3 | 18.4 | 12.3-25.0 | 19.8 | 12.0-27.9 | 15.0 | 4.7-27.0 |
|  | A+T+ | 7.5 | 3.9-11.6 | 39.7 | 31.8-48.4 | 27.7 | 19.4-37.4 | 70.0 | 55.8-85.3 |
| A2T1 | A-T- | 47.5 | 40.3-54.5 | 22.0 | 15.6-29.7 | 26.7 | 18.1-35.7 | 10.0 | 2.3-20.0 |
|  | A-T+ | 21.0 | 15.3-27.1 | 12.8 | 7.2-18.2 | 17.8 | 10.1-25.5 | 0.0 | 0.0-0.0 |
|  | A+T- | 11.0 | 7.1-15.5 | 10.6 | 5.9-16.4 | 11.9 | 5.9-18.2 | 7.5 | 0.0-17.1 |
|  | A+T+ | 20.5 | 14.8-26.0 | 54.6 | 45.7-63.5 | 43.6 | 34.0-53.6 | 82.5 | 69.8-93.3 |
| A2T2 | A-T- | 62.0 | 55.0-68.8 | 24.8 | 17.5-32.8 | 33.7 | 24.0-42.2 | 2.5 | 0.0-7.7 |
|  | A-T+ | 6.5 | 3.1-10.0 | 9.9 | 5.1-14.9 | 10.9 | 4.9-17.0 | 7.5 | 0.0-17.0 |
|  | A+T- | 17.5 | 12.4-22.8 | 18.4 | 12.3-25.0 | 20.8 | 12.9-28.3 | 12.5 | 2.6-23.5 |
|  | A+T+ | 14.0 | 9.1-19.2 | 46.8 | 38.4-55.6 | 34.7 | 25.5-44.9 | 77.5 | 63.2-90.2 |
| A2T3 | A-T- | 59.5 | 52.5-66.7 | 27.7 | 20.3-35.6 | 36.6 | 26.5-46.7 | 5.0 | 0.0-12.8 |
|  | A-T+ | 9.0 | 5.2-13.2 | 7.1 | 3.4-11.8 | 7.9 | 3.1-13.5 | 5.0 | 0.0-12.5 |
|  | A+T- | 20.5 | 15.1-26.3 | 21.3 | 14.7-28.0 | 23.8 | 15.8-32.0 | 15.0 | 4.7-27.0 |
|  | A+T+ | 11.0 | 6.7-15.6 | 44.0 | 35.6-52.9 | 31.7 | 22.7-41.9 | 75.0 | 60.5-88.0 |

95% CI: 95% confidence interval generated using bootstrap resampling (n=1,000).

## Supplementary Table 5 Prevalence of different AT(N) variants among 4 groups

| AT(N) variants | category | CU | | CI | | MCI | | AD | |
| --- | --- | --- | --- | --- | --- | --- | --- | --- | --- |
|  |  | frequency, % | 95% CI, % | frequency, % | 95% CI, % | frequency, % | 95% CI, % | frequency, % | 95% CI, % |
| A1T1N1 | A-T-N- | 32.5 | 26.1-38.5 | 10.6 | 5.8-16.3 | 12.9 | 6.6-19.5 | 5.0 | 0.0-12.8 |
|  | A-T-N+ | 11.0 | 6.8-15.7 | 9.9 | 5.2-15.0 | 11.9 | 6.4-18.4 | 5.0 | 0.0-12.5 |
|  | A-T+N- | 20.0 | 14.5-25.5 | 9.9 | 5.2-15.1 | 13.9 | 7.4-20.7 | 0.0 | 0.0-0.0 |
|  | A-T+N+ | 6.0 | 2.9-9.4 | 11.3 | 6.2-16.7 | 13.9 | 7.4-20.2 | 5.0 | 0.0-12.5 |
|  | A+T-N- | 10.0 | 6.1-14.6 | 3.5 | 0.7-6.7 | 5.0 | 1.0-9.6 | 0.0 | 0.0-0.0 |
|  | A+T-N+ | 5.0 | 2.3-8.1 | 8.5 | 4.4-13.9 | 8.9 | 3.8-15.3 | 7.5 | 0.0-17.1 |
|  | A+T+N- | 12.0 | 7.8-16.6 | 8.5 | 4.4-13.4 | 8.9 | 3.8-15.1 | 7.5 | 0.0-16.7 |
|  | A+T+N+ | 3.5 | 1.1-6.1 | 37.6 | 28.7-45.8 | 24.8 | 16.3-33.3 | 70.0 | 55.3-84.2 |
| A1T1N2 | A-T-N- | 35.2 | 28.5-41.8 | 12.1 | 6.8-18.1 | 14.9 | 7.9-21.9 | 5.0 | 0.0-12.8 |
|  | A-T-N+ | 8.5 | 4.8-12.4 | 8.5 | 4.2-13.3 | 9.9 | 4.3-16.0 | 5.0 | 0.0-12.5 |
|  | A-T+N- | 20.6 | 15.5-26.1 | 9.2 | 4.2-14.6 | 12.9 | 6.5-19.6 | 0.0 | 0.0-0.0 |
|  | A-T+N+ | 5.5 | 2.5-9.4 | 12.1 | 6.7-18.1 | 14.9 | 7.9-21.6 | 5.0 | 0.0-12.5 |
|  | A+T-N- | 11.6 | 7.3-15.9 | 4.3 | 1.4-8.2 | 5.0 | 1.1-9.8 | 2.5 | 0.0-8.2 |
|  | A+T-N+ | 3.5 | 1.4-6.6 | 7.8 | 3.7-12.9 | 8.9 | 3.7-15.0 | 5.0 | 0.0-12.5 |
|  | A+T+N- | 12.1 | 7.7-16.7 | 10.6 | 5.6-15.6 | 11.9 | 6.0-18.6 | 7.5 | 0.0-16.0 |
|  | A+T+N+ | 3.0 | 1.0-5.5 | 35.5 | 27.1-43.7 | 21.8 | 14.1-30.5 | 70.0 | 56.0-83.7 |
| A1T1N3 | A-T-N- | 39.5 | 32.8-46.4 | 17.7 | 11.4-24.3 | 21.8 | 13.7-30.1 | 7.5 | 0.0-17.0 |
|  | A-T-N+ | 4.0 | 1.5-6.8 | 2.8 | 0.7-6.0 | 3.0 | 0.0-6.9 | 2.5 | 0.0-8.3 |
|  | A-T+N- | .5 | 0.0-1.6 | 0.0 | 0.0-0.0 | 0.0 | 0.0-0.0 | 0.0 | 0.0-0.0 |
|  | A-T+N+ | 25.5 | 19.7-31.5 | 21.3 | 14.6-28.5 | 27.7 | 18.6-36.5 | 5.0 | 0.0-12.5 |
|  | A+T-N- | 14.0 | 9.5-18.8 | 12.1 | 6.9-18.1 | 13.9 | 7.7-20.9 | 7.5 | 0.0-17.1 |
|  | A+T-N+ | 1.0 | 0.0-2.7 | 0.0 | 0.0-0.0 | 0.0 | 0.0-0.0 | 0.0 | 0.0-0.0 |
|  | A+T+N- | 1.0 | 0.0-2.6 | 2.8 | 0.0-6.1 | 3.0 | 0.0-7.1 | 2.5 | 0.0-8.1 |
|  | A+T+N+ | 14.5 | 9.8-19.5 | 43.3 | 34.3-51.4 | 30.7 | 21.7-40.0 | 75.0 | 61.4-88.2 |
| A1T1N4 | A-T-N- | 34.0 | 26.6-41.9 | 11.7 | 6.5-17.3 | 15.2 | 8.5-22.8 | 2.6 | 0.0-9.4 |
|  | A-T-N+ | 10.6 | 6.0-16.3 | 9.5 | 4.6-14.9 | 10.1 | 4.5-16.2 | 7.9 | 0.0-17.5 |
|  | A-T+N- | 24.8 | 17.7-32.9 | 10.9 | 6.1-16.6 | 15.2 | 8.3-23.1 | 0.0 | 0.0-0.0 |
|  | A-T+N+ | 5.7 | 2.1-9.9 | 10.2 | 5.4-15.8 | 12.1 | 5.5-18.9 | 5.3 | 0.0-12.5 |
|  | A+T-N- | 6.4 | 2.9-10.7 | 4.4 | 1.4-7.8 | 5.1 | 1.0-9.9 | 2.6 | 0.0-9.1 |
|  | A+T-N+ | 5.7 | 2.1-9.9 | 7.3 | 3.2-11.6 | 9.1 | 3.9-15.2 | 2.6 | 0.0-8.8 |
|  | A+T+N- | 10.6 | 5.4-15.9 | 10.9 | 5.8-16.3 | 12.1 | 5.7-19.0 | 7.9 | 0.0-17.9 |
|  | A+T+N+ | 2.1 | 0.0-4.7 | 35.0 | 27.9-43.5 | 21.2 | 12.7-30.3 | 71.1 | 55.6-84.8 |
| A1T1N5 | A-T-N- | 27.5 | 19.9-35.4 | 9.5 | 4.7-15.4 | 12.0 | 5.6-19.2 | 3.0 | 0.0-10.2 |
|  | A-T-N+ | 15.9 | 10.1-22.0 | 12.9 | 6.9-19.5 | 14.5 | 7.4-22.4 | 9.1 | 0.0-20.7 |
|  | A-T+N- | 15.9 | 10.1-22.6 | 10.3 | 5.0-16.3 | 14.5 | 7.0-22.8 | 0.0 | 0.0-0.0 |
|  | A-T+N+ | 15.2 | 9.3-21.9 | 12.1 | 6.5-18.1 | 14.5 | 7.1-22.9 | 6.1 | 0.0-16.1 |
|  | A+T-N- | 4.3 | 1.4-8.0 | 2.6 | 0.0-6.0 | 3.6 | 0.0-8.0 | 0.0 | 0.0-0.0 |
|  | A+T-N+ | 8.0 | 3.6-12.7 | 8.6 | 3.6-13.9 | 9.6 | 4.3-17.1 | 6.1 | 0.0-16.1 |
|  | A+T+N- | 4.3 | 1.4-8.0 | 7.8 | 2.8-13.5 | 7.2 | 2.3-13.6 | 9.1 | 0.0-19.6 |
|  | A+T+N+ | 8.7 | 4.2-14.2 | 36.2 | 27.2-45.2 | 24.1 | 15.0-34.2 | 66.7 | 48.6-81.8 |
| A1T2N1 | A-T-N- | 45.0 | 38.5-51.5 | 17.0 | 11.2-23.9 | 22.8 | 14.4-31.3 | 2.5 | 0.0-7.7 |
|  | A-T-N+ | 15.0 | 10.3-19.9 | 10.6 | 6.0-16.3 | 14.9 | 8.5-22.2 | 0.0 | 0.0-0.0 |
|  | A-T+N- | 7.5 | 4.2-11.3 | 3.5 | 0.7-7.0 | 4.0 | 0.9-8.5 | 2.5 | 0.0-8.7 |
|  | A-T+N+ | 2.0 | 0.5-4.1 | 10.6 | 5.8-16.2 | 10.9 | 5.0-17.1 | 10.0 | 2.2-20.0 |
|  | A+T-N- | 15.5 | 10.4-21.1 | 5.0 | 1.6-8.5 | 5.9 | 1.9-11.2 | 2.5 | 0.0-8.3 |
|  | A+T-N+ | 4.0 | 1.5-6.9 | 10.6 | 5.8-16.2 | 10.9 | 5.2-17.3 | 10.0 | 2.0-19.6 |
|  | A+T+N- | 6.5 | 3.1-10.3 | 7.1 | 3.3-11.7 | 7.9 | 3.0-13.8 | 5.0 | 0.0-12.8 |
|  | A+T+N+ | 4.5 | 2.0-7.7 | 35.5 | 27.7-43.2 | 22.8 | 15.0-31.5 | 67.5 | 52.5-82.9 |
| A1T2N2 | A-T-N- | 49.2 | 42.6-56.2 | 18.4 | 11.9-25.4 | 24.8 | 15.3-33.3 | 2.5 | 0.0-7.7 |
|  | A-T-N+ | 11.1 | 6.7-15.3 | 9.2 | 4.7-14.6 | 12.9 | 6.9-19.6 | 0.0 | 0.0-0.0 |
|  | A-T+N- | 6.5 | 3.4-9.9 | 2.8 | 0.6-6.0 | 3.0 | 0.0-6.4 | 2.5 | 0.0-8.7 |
|  | A-T+N+ | 3.0 | 1.0-5.7 | 11.3 | 6.3-16.9 | 11.9 | 5.7-18.2 | 10.0 | 2.2-20.0 |
|  | A+T-N- | 15.6 | 10.7-21.2 | 6.4 | 2.7-10.8 | 6.9 | 2.1-12.5 | 5.0 | 0.0-12.2 |
|  | A+T-N+ | 3.5 | 1.1-6.7 | 9.2 | 4.7-14.3 | 9.9 | 4.0-16.2 | 7.5 | 0.0-16.7 |
|  | A+T+N- | 8.0 | 4.4-12.1 | 8.5 | 4.3-13.0 | 9.9 | 4.6-16.0 | 5.0 | 0.0-12.2 |
|  | A+T+N+ | 3.0 | 1.0-5.7 | 34.0 | 26.5-42.4 | 20.8 | 12.9-29.8 | 67.5 | 52.9-82.1 |
| A1T2N3 | A-T-N- | 36.5 | 29.7-42.9 | 12.8 | 7.5-19.0 | 16.8 | 9.3-24.3 | 2.5 | 0.0-7.7 |
|  | A-T-N+ | 23.5 | 17.8-29.3 | 14.9 | 9.0-20.6 | 20.8 | 12.5-28.4 | 0.0 | 0.0-0.0 |
|  | A-T+N- | 3.5 | 1.0-6.1 | 5.0 | 1.5-9.0 | 5.0 | 1.0-9.4 | 5.0 | 0.0-12.8 |
|  | A-T+N+ | 6.0 | 2.9-9.5 | 9.2 | 4.4-14.4 | 9.9 | 4.4-15.9 | 7.5 | 0.0-16.7 |
|  | A+T-N- | 11.5 | 7.3-16.1 | 6.4 | 2.9-10.8 | 7.9 | 2.9-13.6 | 2.5 | 0.0-8.2 |
|  | A+T-N+ | 8.0 | 4.4-12.2 | 9.2 | 4.7-14.3 | 8.9 | 3.8-14.9 | 10.0 | 0.0-20.5 |
|  | A+T+N- | 3.5 | 1.1-6.5 | 8.5 | 4.2-13.8 | 8.9 | 3.8-14.9 | 7.5 | 0.0-16.3 |
|  | A+T+N+ | 7.5 | 4.0-11.5 | 34.0 | 25.7-42.4 | 21.8 | 14.0-30.9 | 65.0 | 50.0-80.0 |
| A1T2N4 | A-T-N- | 48.9 | 40.7-57.6 | 16.1 | 10.1-22.4 | 21.2 | 13.0-29.3 | 2.6 | 0.0-9.4 |
|  | A-T-N+ | 13.5 | 8.2-19.3 | 11.7 | 6.3-17.6 | 16.2 | 9.2-23.8 | 0.0 | 0.0-0.0 |
|  | A-T+N- | 9.9 | 5.4-15.3 | 6.6 | 2.8-11.3 | 9.1 | 4.1-15.7 | 0.0 | 0.0-0.0 |
|  | A-T+N+ | 2.8 | 0.6-6.0 | 8.0 | 3.6-13.2 | 6.1 | 1.9-10.9 | 13.2 | 3.0-24.4 |
|  | A+T-N- | 7.8 | 3.7-12.4 | 5.1 | 1.5-9.2 | 5.1 | 1.0-9.7 | 5.3 | 0.0-13.6 |
|  | A+T-N+ | 5.0 | 1.9-8.8 | 10.2 | 5.7-15.9 | 12.1 | 6.3-19.6 | 5.3 | 0.0-13.8 |
|  | A+T+N- | 9.2 | 4.5-14.6 | 10.2 | 5.3-15.4 | 12.1 | 5.8-19.0 | 5.3 | 0.0-13.8 |
|  | A+T+N+ | 2.8 | 0.6-6.0 | 32.1 | 25.4-40.4 | 18.2 | 10.9-25.9 | 68.4 | 52.3-83.7 |
| A1T2N5 | A-T-N- | 39.9 | 31.6-48.4 | 14.7 | 8.3-21.1 | 19.3 | 11.3-28.0 | 3.0 | 0.0-10.2 |
|  | A-T-N+ | 21.7 | 14.9-28.6 | 13.8 | 8.0-20.5 | 19.3 | 10.7-27.9 | 0.0 | 0.0-0.0 |
|  | A-T+N- | 3.6 | 0.7-7.3 | 5.2 | 1.7-9.7 | 7.2 | 2.3-13.4 | 0.0 | 0.0-0.0 |
|  | A-T+N+ | 9.4 | 5.0-14.3 | 11.2 | 5.8-17.0 | 9.6 | 3.7-16.7 | 15.2 | 3.3-29.2 |
|  | A+T-N- | 4.3 | 1.4-8.1 | 6.0 | 1.9-11.2 | 7.2 | 1.4-13.4 | 3.0 | 0.0-9.7 |
|  | A+T-N+ | 8.7 | 4.3-13.9 | 10.3 | 4.8-16.4 | 10.8 | 4.9-18.6 | 9.1 | 0.0-21.6 |
|  | A+T+N- | 4.3 | 1.4-8.2 | 4.3 | 0.9-8.5 | 3.6 | 0.0-7.9 | 6.1 | 0.0-15.4 |
|  | A+T+N+ | 8.0 | 3.6-13.1 | 34.5 | 25.5-43.8 | 22.9 | 13.4-32.4 | 63.6 | 46.9-79.3 |
| A1T3N1 | A-T-N- | 42.5 | 35.8-49.0 | 17.7 | 11.7-24.4 | 22.8 | 14.4-31.1 | 5.0 | 0.0-12.8 |
|  | A-T-N+ | 14.5 | 10.0-19.6 | 12.8 | 7.5-18.7 | 17.8 | 10.5-25.0 | 0.0 | 0.0-0.0 |
|  | A-T+N- | 10.0 | 6.0-14.3 | 2.8 | 0.7-5.8 | 4.0 | 0.9-8.3 | 0.0 | 0.0-0.0 |
|  | A-T+N+ | 2.5 | 0.5-5.0 | 8.5 | 4.3-13.4 | 7.9 | 2.9-13.0 | 10.0 | 2.2-20.0 |
|  | A+T-N- | 16.0 | 10.6-21.6 | 5.7 | 2.2-9.5 | 6.9 | 2.7-12.8 | 2.5 | 0.0-8.3 |
|  | A+T-N+ | 7.0 | 3.8-10.6 | 12.8 | 7.4-18.4 | 12.9 | 6.6-19.6 | 12.5 | 2.6-23.7 |
|  | A+T+N- | 6.0 | 2.8-9.6 | 6.4 | 2.8-10.8 | 6.9 | 2.2-12.4 | 5.0 | 0.0-12.8 |
|  | A+T+N+ | 1.5 | 0.0-3.6 | 33.3 | 25.8-41.6 | 20.8 | 13.3-29.4 | 65.0 | 50.0-80.6 |
| A1T3N2 | A-T-N- | 46.7 | 40.1-53.8 | 18.4 | 12.0-25.0 | 23.8 | 15.2-32.0 | 5.0 | 0.0-12.8 |
|  | A-T-N+ | 10.6 | 6.1-15.3 | 12.1 | 6.9-18.0 | 16.8 | 9.9-24.4 | 0.0 | 0.0-0.0 |
|  | A-T+N- | 9.0 | 5.2-13.2 | 2.8 | 0.7-6.0 | 4.0 | 0.9-8.2 | 0.0 | 0.0-0.0 |
|  | A-T+N+ | 3.5 | 1.1-6.3 | 8.5 | 4.3-13.3 | 7.9 | 2.9-13.0 | 10.0 | 2.2-20.0 |
|  | A+T-N- | 19.1 | 13.8-24.5 | 7.1 | 3.2-11.7 | 8.9 | 3.4-15.5 | 2.5 | 0.0-8.3 |
|  | A+T-N+ | 3.5 | 1.1-6.7 | 11.3 | 6.4-16.5 | 10.9 | 5.0-16.8 | 12.5 | 2.6-23.3 |
|  | A+T+N- | 4.5 | 1.6-7.5 | 7.8 | 3.8-12.4 | 7.9 | 2.9-13.3 | 7.5 | 0.0-15.6 |
|  | A+T+N+ | 3.0 | 1.0-5.7 | 31.9 | 24.3-40.4 | 19.8 | 12.2-28.6 | 62.5 | 47.5-78.6 |
| A1T3N3 | A-T-N- | 35.5 | 28.1-42.4 | 15.6 | 9.6-22.1 | 19.8 | 11.8-28.0 | 5.0 | 0.0-12.8 |
|  | A-T-N+ | 21.5 | 16.0-27.4 | 14.9 | 9.0-21.0 | 20.8 | 12.8-28.9 | 0.0 | 0.0-0.0 |
|  | A-T+N- | 4.5 | 2.0-7.5 | 2.1 | 0.0-4.8 | 2.0 | 0.0-5.3 | 2.5 | 0.0-8.3 |
|  | A-T+N+ | 8.0 | 4.5-11.7 | 9.2 | 4.7-14.4 | 9.9 | 4.0-16.0 | 7.5 | 0.0-16.7 |
|  | A+T-N- | 13.0 | 8.5-17.8 | 7.1 | 3.3-11.5 | 8.9 | 3.8-14.9 | 2.5 | 0.0-8.1 |
|  | A+T-N+ | 10.0 | 6.2-14.4 | 11.3 | 6.3-16.4 | 10.9 | 5.3-17.1 | 12.5 | 2.6-23.3 |
|  | A+T+N- | 2.0 | 0.5-4.3 | 7.8 | 3.6-12.9 | 7.9 | 3.1-13.3 | 7.5 | 0.0-17.1 |
|  | A+T+N+ | 5.5 | 2.5-9.0 | 31.9 | 24.0-40.3 | 19.8 | 12.4-29.1 | 62.5 | 47.4-77.8 |
| A1T3N4 | A-T-N- | 46.8 | 38.5-54.5 | 16.8 | 10.5-23.0 | 22.2 | 14.6-30.5 | 2.6 | 0.0-9.4 |
|  | A-T-N+ | 12.1 | 7.0-17.5 | 13.9 | 8.3-20.0 | 18.2 | 10.6-25.9 | 2.6 | 0.0-8.7 |
|  | A-T+N- | 12.1 | 6.7-17.9 | 5.8 | 2.2-9.9 | 8.1 | 2.9-14.5 | 0.0 | 0.0-0.0 |
|  | A-T+N+ | 4.3 | 1.3-8.0 | 5.8 | 2.2-10.3 | 4.0 | 0.9-8.2 | 10.5 | 2.3-21.2 |
|  | A+T-N- | 9.9 | 5.5-15.0 | 5.8 | 2.2-10.2 | 7.1 | 2.2-12.6 | 2.6 | 0.0-9.1 |
|  | A+T-N+ | 6.4 | 2.3-10.5 | 12.4 | 7.5-18.7 | 13.1 | 7.1-21.1 | 10.5 | 2.6-21.1 |
|  | A+T+N- | 7.1 | 3.3-11.9 | 9.5 | 4.6-14.5 | 10.1 | 4.2-16.3 | 7.9 | 0.0-17.9 |
|  | A+T+N+ | 1.4 | 0.0-3.7 | 29.9 | 23.3-38.6 | 17.2 | 10.1-24.7 | 63.2 | 47.4-78.9 |
| A1T3N5 | A-T-N- | 34.8 | 26.9-43.1 | 16.4 | 10.2-23.4 | 21.7 | 13.1-30.3 | 3.0 | 0.0-10.2 |
|  | A-T-N+ | 23.9 | 16.8-31.2 | 14.7 | 8.3-21.4 | 19.3 | 11.0-28.2 | 3.0 | 0.0-10.7 |
|  | A-T+N- | 8.7 | 4.1-14.1 | 3.4 | 0.8-7.2 | 4.8 | 1.1-10.0 | 0.0 | 0.0-0.0 |
|  | A-T+N+ | 7.2 | 3.4-11.5 | 10.3 | 5.1-16.1 | 9.6 | 4.1-16.7 | 12.1 | 2.7-25.0 |
|  | A+T-N- | 6.5 | 2.7-10.8 | 6.0 | 1.9-10.8 | 7.2 | 1.5-13.4 | 3.0 | 0.0-9.7 |
|  | A+T-N+ | 10.1 | 5.5-15.6 | 12.9 | 6.3-19.1 | 13.3 | 6.7-21.5 | 12.1 | 2.7-25.0 |
|  | A+T+N- | 2.2 | 0.0-4.8 | 4.3 | 0.9-8.4 | 3.6 | 0.0-8.2 | 6.1 | 0.0-15.4 |
|  | A+T+N+ | 6.5 | 2.8-11.2 | 31.9 | 23.5-40.8 | 20.5 | 11.5-29.6 | 60.6 | 44.1-76.0 |
| A2T1N1 | A-T-N- | 37.0 | 30.3-44.2 | 11.3 | 6.3-17.0 | 13.9 | 7.2-21.2 | 5.0 | 0.0-12.8 |
|  | A-T-N+ | 10.5 | 6.4-15.3 | 10.6 | 5.7-16.2 | 12.9 | 6.6-19.7 | 5.0 | 0.0-12.5 |
|  | A-T+N- | 17.5 | 12.4-23.3 | 6.4 | 2.6-10.8 | 8.9 | 3.1-14.3 | 0.0 | 0.0-0.0 |
|  | A-T+N+ | 3.5 | 1.1-6.5 | 6.4 | 2.4-10.7 | 8.9 | 3.8-14.7 | 0.0 | 0.0-0.0 |
|  | A+T-N- | 5.5 | 2.6-8.9 | 2.8 | 0.7-6.0 | 4.0 | 0.9-8.2 | 0.0 | 0.0-0.0 |
|  | A+T-N+ | 5.5 | 2.6-8.8 | 7.8 | 3.8-13.0 | 7.9 | 3.0-13.5 | 7.5 | 0.0-17.1 |
|  | A+T+N- | 14.5 | 9.5-19.5 | 12.1 | 6.7-17.8 | 13.9 | 7.8-21.3 | 7.5 | 0.0-16.7 |
|  | A+T+N+ | 6.0 | 2.9-9.4 | 42.6 | 33.8-51.2 | 29.7 | 20.7-39.0 | 75.0 | 61.0-87.8 |
| A2T1N2 | A-T-N- | 38.7 | 32.0-45.2 | 13.5 | 8.3-19.7 | 16.8 | 9.5-24.5 | 5.0 | 0.0-12.8 |
|  | A-T-N+ | 9.0 | 4.9-13.2 | 8.5 | 4.1-13.4 | 9.9 | 4.2-16.2 | 5.0 | 0.0-12.5 |
|  | A-T+N- | 16.6 | 11.3-21.3 | 6.4 | 2.6-10.7 | 8.9 | 3.2-14.6 | 0.0 | 0.0-0.0 |
|  | A-T+N+ | 4.0 | 1.5-7.1 | 6.4 | 2.3-10.6 | 8.9 | 4.0-14.4 | 0.0 | 0.0-0.0 |
|  | A+T-N- | 8.0 | 4.7-11.9 | 2.8 | 0.7-6.1 | 3.0 | 0.0-6.6 | 2.5 | 0.0-8.2 |
|  | A+T-N+ | 3.0 | 1.0-5.6 | 7.8 | 3.5-12.8 | 8.9 | 3.7-14.5 | 5.0 | 0.0-12.5 |
|  | A+T+N- | 16.1 | 11.1-21.4 | 13.5 | 7.8-19.4 | 15.8 | 8.5-23.7 | 7.5 | 0.0-16.0 |
|  | A+T+N+ | 4.5 | 1.9-7.7 | 41.1 | 32.4-49.3 | 27.7 | 19.1-37.5 | 75.0 | 60.9-87.5 |
| A2T1N3 | A-T-N- | 44.5 | 37.4-51.5 | 19.9 | 13.4-27.0 | 24.8 | 16.2-33.3 | 7.5 | 0.0-17.0 |
|  | A-T-N+ | 3.0 | 0.9-5.5 | 2.1 | 0.0-5.0 | 2.0 | 0.0-5.0 | 2.5 | 0.0-8.3 |
|  | A-T+N- | 1.0 | 0.0-2.8 | 0.0 | 0.0-0.0 | 0.0 | 0.0-0.0 | 0.0 | 0.0-0.0 |
|  | A-T+N+ | 20.0 | 14.5-25.7 | 12.8 | 7.2-18.2 | 17.8 | 10.1-25.5 | 0.0 | 0.0-0.0 |
|  | A+T-N- | 9.0 | 5.3-13.3 | 9.9 | 5.3-15.7 | 10.9 | 5.5-17.2 | 7.5 | 0.0-17.1 |
|  | A+T-N+ | 2.0 | 0.5-4.2 | .7 | 0.0-2.3 | 1.0 | 0.0-3.3 | 0.0 | 0.0-0.0 |
|  | A+T+N- | .5 | 0.0-1.6 | 2.8 | 0.0-6.1 | 3.0 | 0.0-7.1 | 2.5 | 0.0-8.1 |
|  | A+T+N+ | 20.0 | 14.2-25.7 | 51.8 | 43.2-60.3 | 40.6 | 31.3-50.6 | 80.0 | 67.6-92.1 |
| A2T1N4 | A-T-N- | 33.3 | 25.3-41.0 | 12.4 | 7.1-18.0 | 16.2 | 8.8-24.3 | 2.6 | 0.0-9.4 |
|  | A-T-N+ | 12.1 | 7.4-18.1 | 10.2 | 5.4-15.9 | 11.1 | 5.1-17.6 | 7.9 | 0.0-17.5 |
|  | A-T+N- | 21.3 | 14.1-28.7 | 6.6 | 2.9-11.2 | 9.1 | 4.1-15.2 | 0.0 | 0.0-0.0 |
|  | A-T+N+ | 1.4 | 0.0-3.7 | 5.8 | 2.2-10.2 | 8.1 | 3.2-13.9 | 0.0 | 0.0-0.0 |
|  | A+T-N- | 7.1 | 3.5-11.8 | 3.6 | 0.8-7.0 | 4.0 | 0.9-8.0 | 2.6 | 0.0-9.1 |
|  | A+T-N+ | 4.3 | 1.3-8.1 | 6.6 | 2.9-11.0 | 8.1 | 3.2-14.1 | 2.6 | 0.0-8.8 |
|  | A+T+N- | 14.2 | 8.3-20.7 | 15.3 | 9.3-21.3 | 18.2 | 11.0-26.4 | 7.9 | 0.0-17.9 |
|  | A+T+N+ | 6.4 | 2.8-10.9 | 39.4 | 32.0-48.2 | 25.3 | 16.2-34.0 | 76.3 | 61.3-89.2 |
| A2T1N5 | A-T-N- | 26.1 | 18.7-33.3 | 10.3 | 5.1-16.2 | 13.3 | 6.3-20.7 | 3.0 | 0.0-10.2 |
|  | A-T-N+ | 18.1 | 11.9-24.6 | 12.9 | 7.2-19.3 | 14.5 | 7.1-22.6 | 9.1 | 0.0-20.7 |
|  | A-T+N- | 14.5 | 8.8-20.6 | 6.9 | 2.7-11.9 | 9.6 | 3.7-16.7 | 0.0 | 0.0-0.0 |
|  | A-T+N+ | 8.7 | 4.2-13.7 | 5.2 | 1.7-9.4 | 7.2 | 2.4-13.3 | 0.0 | 0.0-0.0 |
|  | A+T-N- | 5.8 | 2.1-10.1 | 1.7 | 0.0-4.5 | 2.4 | 0.0-6.2 | 0.0 | 0.0-0.0 |
|  | A+T-N+ | 5.8 | 2.2-10.0 | 8.6 | 3.7-14.0 | 9.6 | 3.9-16.5 | 6.1 | 0.0-16.1 |
|  | A+T+N- | 5.8 | 2.2-9.8 | 11.2 | 5.6-17.8 | 12.0 | 5.4-19.5 | 9.1 | 0.0-19.6 |
|  | A+T+N+ | 15.2 | 9.6-21.7 | 43.1 | 34.2-52.3 | 31.3 | 21.4-41.3 | 72.7 | 56.7-86.5 |
| A2T2N1 | A-T-N- | 49.0 | 42.0-56.0 | 15.6 | 9.5-22.2 | 20.8 | 12.8-29.1 | 2.5 | 0.0-7.7 |
|  | A-T-N+ | 13.0 | 8.4-18.3 | 9.2 | 5.0-14.4 | 12.9 | 6.9-19.3 | 0.0 | 0.0-0.0 |
|  | A-T+N- | 5.5 | 2.6-8.7 | 2.1 | 0.0-4.9 | 2.0 | 0.0-5.2 | 2.5 | 0.0-8.7 |
|  | A-T+N+ | 1.0 | 0.0-2.6 | 7.8 | 3.7-12.5 | 8.9 | 3.9-14.6 | 5.0 | 0.0-12.5 |
|  | A+T-N- | 11.5 | 7.1-16.2 | 6.4 | 2.8-10.6 | 7.9 | 3.0-13.9 | 2.5 | 0.0-8.3 |
|  | A+T-N+ | 6.0 | 2.9-9.4 | 12.1 | 6.9-17.7 | 12.9 | 6.5-19.4 | 10.0 | 2.0-19.6 |
|  | A+T+N- | 8.5 | 4.6-12.8 | 8.5 | 3.9-13.4 | 9.9 | 4.7-16.2 | 5.0 | 0.0-12.8 |
|  | A+T+N+ | 5.5 | 2.5-8.8 | 38.3 | 30.0-46.2 | 24.8 | 16.3-33.3 | 72.5 | 57.8-86.7 |
| A2T2N2 | A-T-N- | 50.8 | 44.1-58.0 | 17.0 | 10.9-23.9 | 22.8 | 14.0-30.9 | 2.5 | 0.0-7.7 |
|  | A-T-N+ | 11.1 | 6.8-15.6 | 7.8 | 3.8-12.7 | 10.9 | 5.3-16.9 | 0.0 | 0.0-0.0 |
|  | A-T+N- | 4.5 | 1.5-7.5 | 2.8 | 0.6-6.1 | 3.0 | 0.0-6.7 | 2.5 | 0.0-8.7 |
|  | A-T+N+ | 2.0 | 0.5-4.3 | 7.1 | 3.1-11.7 | 7.9 | 3.1-13.5 | 5.0 | 0.0-12.5 |
|  | A+T-N- | 14.1 | 9.6-19.0 | 7.8 | 3.7-12.5 | 8.9 | 3.8-15.0 | 5.0 | 0.0-12.2 |
|  | A+T-N+ | 3.5 | 1.4-6.4 | 10.6 | 6.0-15.8 | 11.9 | 5.7-18.2 | 7.5 | 0.0-16.7 |
|  | A+T+N- | 10.1 | 6.2-14.6 | 8.5 | 4.4-12.9 | 9.9 | 4.4-16.3 | 5.0 | 0.0-12.2 |
|  | A+T+N+ | 4.0 | 1.6-6.9 | 38.3 | 29.9-47.0 | 24.8 | 16.3-34.3 | 72.5 | 57.6-86.2 |
| A2T2N3 | A-T-N- | 42.0 | 34.7-48.6 | 13.5 | 8.2-19.4 | 17.8 | 10.5-26.1 | 2.5 | 0.0-7.7 |
|  | A-T-N+ | 20.0 | 14.4-26.1 | 11.3 | 6.3-16.8 | 15.8 | 8.9-22.8 | 0.0 | 0.0-0.0 |
|  | A-T+N- | 3.5 | 1.1-6.3 | 6.4 | 2.6-10.7 | 6.9 | 2.2-12.4 | 5.0 | 0.0-12.8 |
|  | A-T+N+ | 3.0 | 1.0-5.4 | 3.5 | 0.7-6.8 | 4.0 | 0.9-8.2 | 2.5 | 0.0-8.3 |
|  | A+T-N- | 6.0 | 3.0-9.4 | 5.7 | 2.1-10.1 | 6.9 | 2.3-11.8 | 2.5 | 0.0-8.2 |
|  | A+T-N+ | 11.5 | 7.3-16.3 | 12.8 | 7.2-18.1 | 13.9 | 7.5-20.8 | 10.0 | 0.0-20.5 |
|  | A+T+N- | 3.5 | 1.0-6.2 | 7.1 | 3.1-11.8 | 6.9 | 2.2-12.0 | 7.5 | 0.0-16.3 |
|  | A+T+N+ | 10.5 | 6.1-15.1 | 39.7 | 31.8-47.8 | 27.7 | 19.5-37.9 | 70.0 | 55.9-83.3 |
| A2T2N4 | A-T-N- | 46.8 | 38.6-55.2 | 14.6 | 9.2-20.9 | 19.2 | 11.5-27.7 | 2.6 | 0.0-9.4 |
|  | A-T-N+ | 12.8 | 7.6-18.4 | 10.2 | 5.5-15.7 | 14.1 | 7.3-21.4 | 0.0 | 0.0-0.0 |
|  | A-T+N- | 7.8 | 3.5-12.8 | 4.4 | 1.4-8.0 | 6.1 | 2.0-11.7 | 0.0 | 0.0-0.0 |
|  | A-T+N+ | .7 | 0.0-2.4 | 5.8 | 2.2-10.2 | 5.1 | 1.1-9.8 | 7.9 | 0.0-17.5 |
|  | A+T-N- | 9.9 | 5.3-15.0 | 6.6 | 2.4-10.7 | 7.1 | 2.1-12.6 | 5.3 | 0.0-13.6 |
|  | A+T-N+ | 5.7 | 2.1-9.4 | 11.7 | 6.3-17.8 | 14.1 | 7.7-22.0 | 5.3 | 0.0-13.8 |
|  | A+T+N- | 11.3 | 6.3-17.2 | 12.4 | 6.8-18.2 | 15.2 | 8.2-22.3 | 5.3 | 0.0-13.8 |
|  | A+T+N+ | 5.0 | 1.5-8.9 | 34.3 | 27.3-42.9 | 19.2 | 11.8-27.1 | 73.7 | 57.9-87.9 |
| A2T2N5 | A-T-N- | 37.7 | 29.9-46.0 | 14.7 | 8.5-21.4 | 19.3 | 11.1-28.2 | 3.0 | 0.0-10.2 |
|  | A-T-N+ | 21.0 | 14.4-28.3 | 10.3 | 5.2-16.2 | 14.5 | 7.3-22.6 | 0.0 | 0.0-0.0 |
|  | A-T+N- | 2.9 | 0.7-6.1 | 2.6 | 0.0-5.8 | 3.6 | 0.0-8.0 | 0.0 | 0.0-0.0 |
|  | A-T+N+ | 5.8 | 2.3-9.7 | 7.8 | 3.2-12.7 | 7.2 | 1.6-13.5 | 9.1 | 0.0-20.7 |
|  | A+T-N- | 6.5 | 2.3-11.0 | 6.0 | 1.8-10.6 | 7.2 | 2.4-13.2 | 3.0 | 0.0-9.7 |
|  | A+T-N+ | 9.4 | 4.8-14.7 | 13.8 | 8.1-20.8 | 15.7 | 7.6-24.1 | 9.1 | 0.0-21.6 |
|  | A+T+N- | 5.1 | 1.5-9.0 | 6.9 | 2.6-12.1 | 7.2 | 2.1-13.2 | 6.1 | 0.0-15.4 |
|  | A+T+N+ | 11.6 | 6.6-17.3 | 37.9 | 29.0-46.9 | 25.3 | 15.7-35.3 | 69.7 | 53.3-84.6 |
| A2T3N1 | A-T-N- | 46.5 | 39.1-53.3 | 17.0 | 10.7-23.8 | 21.8 | 13.3-30.6 | 5.0 | 0.0-12.8 |
|  | A-T-N+ | 13.0 | 8.4-18.5 | 10.6 | 6.0-15.9 | 14.9 | 8.5-22.0 | 0.0 | 0.0-0.0 |
|  | A-T+N- | 8.0 | 4.4-11.8 | .7 | 0.0-2.3 | 1.0 | 0.0-3.3 | 0.0 | 0.0-0.0 |
|  | A-T+N+ | 1.0 | 0.0-2.5 | 6.4 | 2.8-10.6 | 6.9 | 2.2-12.1 | 5.0 | 0.0-12.5 |
|  | A+T-N- | 12.0 | 7.5-16.8 | 6.4 | 2.8-10.6 | 7.9 | 3.0-13.9 | 2.5 | 0.0-8.3 |
|  | A+T-N+ | 8.5 | 4.7-12.6 | 14.9 | 9.2-20.9 | 15.8 | 9.3-22.9 | 12.5 | 2.6-23.7 |
|  | A+T+N- | 8.0 | 4.3-11.9 | 8.5 | 3.9-13.4 | 9.9 | 4.7-16.2 | 5.0 | 0.0-12.8 |
|  | A+T+N+ | 3.0 | 0.5-5.7 | 35.5 | 27.6-43.4 | 21.8 | 13.6-30.7 | 70.0 | 55.6-84.6 |
| A2T3N2 | A-T-N- | 48.7 | 42.0-56.0 | 17.7 | 11.4-24.6 | 22.8 | 14.3-31.1 | 5.0 | 0.0-12.8 |
|  | A-T-N+ | 10.6 | 6.5-15.2 | 9.9 | 5.3-15.0 | 13.9 | 7.4-21.1 | 0.0 | 0.0-0.0 |
|  | A-T+N- | 6.5 | 3.2-10.0 | 2.1 | 0.0-5.0 | 3.0 | 0.0-6.7 | 0.0 | 0.0-0.0 |
|  | A-T+N+ | 2.5 | 0.5-4.8 | 5.0 | 1.5-9.0 | 5.0 | 1.0-9.6 | 5.0 | 0.0-12.5 |
|  | A+T-N- | 17.1 | 12.1-22.5 | 7.8 | 3.5-12.6 | 9.9 | 4.7-16.3 | 2.5 | 0.0-8.3 |
|  | A+T-N+ | 3.5 | 1.4-6.3 | 13.5 | 8.4-19.0 | 13.9 | 7.6-20.6 | 12.5 | 2.6-23.3 |
|  | A+T+N- | 7.0 | 3.9-10.9 | 8.5 | 4.1-13.4 | 8.9 | 3.6-15.4 | 7.5 | 0.0-15.6 |
|  | A+T+N+ | 4.0 | 1.6-6.8 | 35.5 | 27.1-43.8 | 22.8 | 14.8-31.4 | 67.5 | 52.9-82.3 |
| A2T3N3 | A-T-N- | 41.0 | 33.8-48.0 | 16.3 | 10.4-23.0 | 20.8 | 13.0-29.3 | 5.0 | 0.0-12.8 |
|  | A-T-N+ | 18.5 | 13.1-24.2 | 11.3 | 6.3-16.6 | 15.8 | 9.0-23.5 | 0.0 | 0.0-0.0 |
|  | A-T+N- | 4.5 | 2.0-7.7 | 3.5 | 0.7-6.9 | 4.0 | 0.9-8.3 | 2.5 | 0.0-8.3 |
|  | A-T+N+ | 4.5 | 2.0-7.5 | 3.5 | 0.7-6.7 | 4.0 | 0.9-7.9 | 2.5 | 0.0-8.3 |
|  | A+T-N- | 7.5 | 3.8-11.4 | 6.4 | 2.9-10.9 | 7.9 | 2.8-13.3 | 2.5 | 0.0-8.1 |
|  | A+T-N+ | 13.0 | 8.7-18.1 | 14.9 | 8.9-20.5 | 15.8 | 9.4-23.5 | 12.5 | 2.6-23.3 |
|  | A+T+N- | 2.0 | 0.5-4.3 | 6.4 | 2.8-10.7 | 5.9 | 1.9-11.0 | 7.5 | 0.0-17.1 |
|  | A+T+N+ | 9.0 | 5.1-12.9 | 37.6 | 29.7-46.4 | 25.7 | 17.4-35.1 | 67.5 | 52.8-80.9 |
| A2T3N4 | A-T-N- | 44.7 | 36.1-52.8 | 16.1 | 10.0-22.3 | 21.2 | 13.4-29.7 | 2.6 | 0.0-9.4 |
|  | A-T-N+ | 12.1 | 7.0-17.1 | 11.7 | 6.6-16.9 | 15.2 | 8.2-22.3 | 2.6 | 0.0-8.7 |
|  | A-T+N- | 9.9 | 4.9-15.1 | 2.9 | 0.6-5.8 | 4.0 | 0.9-8.6 | 0.0 | 0.0-0.0 |
|  | A-T+N+ | 1.4 | 0.0-3.8 | 4.4 | 1.4-8.3 | 4.0 | 0.0-8.3 | 5.3 | 0.0-13.9 |
|  | A+T-N- | 12.1 | 7.0-18.0 | 6.6 | 2.9-10.9 | 8.1 | 3.0-13.7 | 2.6 | 0.0-9.1 |
|  | A+T-N+ | 6.4 | 2.7-10.7 | 14.6 | 9.1-21.3 | 16.2 | 9.7-24.7 | 10.5 | 2.6-21.1 |
|  | A+T+N- | 9.2 | 4.9-14.5 | 12.4 | 6.8-18.2 | 14.1 | 7.6-20.9 | 7.9 | 0.0-17.9 |
|  | A+T+N+ | 4.3 | 1.4-7.9 | 31.4 | 24.2-40.0 | 17.2 | 10.2-24.5 | 68.4 | 53.1-83.3 |
| A2T3N5 | A-T-N- | 34.8 | 26.4-43.2 | 15.5 | 9.1-22.0 | 20.5 | 12.2-29.5 | 3.0 | 0.0-10.2 |
|  | A-T-N+ | 21.7 | 15.0-29.0 | 11.2 | 5.6-17.1 | 14.5 | 7.4-22.6 | 3.0 | 0.0-10.7 |
|  | A-T+N- | 5.8 | 2.3-10.4 | 1.7 | 0.0-4.4 | 2.4 | 0.0-6.2 | 0.0 | 0.0-0.0 |
|  | A-T+N+ | 5.1 | 1.7-9.2 | 6.9 | 2.6-11.9 | 7.2 | 2.1-13.6 | 6.1 | 0.0-15.8 |
|  | A+T-N- | 6.5 | 2.8-11.0 | 6.9 | 2.5-11.9 | 8.4 | 2.8-14.9 | 3.0 | 0.0-9.7 |
|  | A+T-N+ | 12.3 | 7.3-18.5 | 16.4 | 9.7-23.3 | 18.1 | 10.5-26.8 | 12.1 | 2.7-25.0 |
|  | A+T+N- | 5.1 | 1.5-8.9 | 6.0 | 1.9-10.9 | 6.0 | 1.3-11.5 | 6.1 | 0.0-15.4 |
|  | A+T+N+ | 8.7 | 4.3-13.8 | 35.3 | 26.5-44.5 | 22.9 | 13.5-32.2 | 66.7 | 50.0-81.8 |

95% CI: 95% confidence interval generated using bootstrap resampling (n=1,000).

## Supplementary Table 6 Linear-mixed effect model for longitudinal cognition using single AT(N) biomarkers

| Group | Scale | AT(N) Biomarkers | Goodness of fit | | p-value | Marginal R2 | Group | Scale | AT(N) Biomarkers | Goodness of fit | | p-value | Marginal R2 |
| --- | --- | --- | --- | --- | --- | --- | --- | --- | --- | --- | --- | --- | --- |
|  |  |  | AIC | BIC | Main effect |  |  |  |  | AIC | BIC | Main effect |  |
| CU | CDRSB | Age  Gender  Education* | 682.798 | 695.594 | 0.027 | NA | CI | CDRSB | Age  Gender  Education* | 1921.046 | 1933.504 | >0.05 | NA |
|  |  |  |  |  | >0.05 |  |  |  |  |  |  | >0.05 |  |
|  |  |  |  |  | 0.048 |  |  |  |  |  |  | >0.05 |  |
|  |  | A1 | 700.724 | 713.514 | 0.872 | 0.059 |  |  | A1 | 1925.851 | 1938.303 | 0.002 | 0.218 |
|  |  | A2 | 684.530 | 697.320 | 0.964 | 0.059 |  |  | A2 | 1910.737 | 1923.189 | 0.003 | 0.212 |
|  |  | T1 | 692.332 | 705.122 | 0.527 | 0.061 |  |  | T1 | 1924.409 | 1936.861 | 0.061 | 0.180 |
|  |  | T2 | 685.108 | 697.898 | 0.640 | 0.060 |  |  | T2 | 1897.068 | 1909.519 | <0.001 | 0.286 |
|  |  | T3 | 683.382 | 696.172 | 0.288 | 0.064 |  |  | T3 | 1902.57 | 1915.022 | <0.001 | 0.257 |
|  |  | N1 | 687.042 | 699.833 | 0.574 | 0.061 |  |  | N1 | 1904.972 | 1917.424 | <0.001 | 0.276 |
|  |  | N2 | 680.015 | 692.806 | 0.047 | 0.075 |  |  | N2 | 1889.918 | 1902.369 | <0.001 | 0.342 |
|  |  | N3 | 696.543 | 709.333 | 0.458 | 0.061 |  |  | N3 | 1928.427 | 1940.879 | 0.051 | 0.182 |
|  |  | N4 | 683.396 | 696.186 | 0.962 | 0.059 |  |  | N4 | 1905.466 | 1917.918 | <0.001 | 0.239 |
|  |  | N5 | 693.167 | 705.957 | 0.797 | 0.059 |  |  | N5 | 1921.978 | 1934.43 | 0.013 | 0.207 |
|  | MMSE | Age  Gender  Education* | 1772.003 | 1784.805 | 0.025 | NA |  | MMSE | Age  Gender  Education* | 1772.003 | 1784.805 | >0.05 | NA |
|  |  |  |  |  | 0.010 |  |  |  |  |  |  | >0.05 |  |
|  |  |  |  |  | <0.001 |  |  |  |  |  |  | >0.05 |  |
|  |  | A1 | 1787.953 | 1800.749 | 0.607 | 0.092 |  |  | A1 | 2183.841 | 2196.228 | <0.001 | 0.206 |
|  |  | A2 | 1769.592 | 1782.388 | 0.119 | 0.099 |  |  | A2 | 2171.181 | 2183.568 | 0.002 | 0.190 |
|  |  | T1 | 1779.522 | 1792.318 | 0.403 | 0.094 |  |  | T1 | 2178.972 | 2191.359 | 0.002 | 0.189 |
|  |  | T2 | 1771.643 | 1784.439 | 0.281 | 0.095 |  |  | T2 | 2148.237 | 2160.624 | <0.001 | 0.288 |
|  |  | T3 | 1771.895 | 1784.691 | 0.753 | 0.092 |  |  | T3 | 2148.643 | 2161.03 | <0.001 | 0.286 |
|  |  | N1 | 1769.781 | 1782.577 | 0.025 | 0.108 |  |  | N1 | 2157.643 | 2170.031 | <0.001 | 0.272 |
|  |  | N2 | 1769.785 | 1782.581 | 0.199 | 0.096 |  |  | N2 | 2148.501 | 2160.889 | <0.001 | 0.295 |
|  |  | N3 | 1783.894 | 1796.69 | 0.410 | 0.094 |  |  | N3 | 2182.307 | 2194.695 | 0.001 | 0.194 |
|  |  | N4 | 1769.495 | 1782.291 | 0.241 | 0.096 |  |  | N4 | 2162.015 | 2174.402 | <0.001 | 0.225 |
|  |  | N5 | 1780.72 | 1793.516 | 0.945 | 0.092 |  |  | N5 | 2179.13 | 2191.517 | 0.001 | 0.198 |

*Zero model is composed of age, gender and education as factors without any AT(N) biomarkers.

AIC: Akaike information criterion; BIC: Bayesian information criterion. In the regression model, both AIC and BIC are as smaller as better.

## Supplementary Table 7 Linear-mixed effect model with interactions for longitudinal cognition in CI using single AT(N) biomarkers

| Scale | AT(N) Biomarkers | Goodness of fit | | p-value | | Marginal R2 |
| --- | --- | --- | --- | --- | --- | --- |
|  |  | AIC | BIC | Main effect | Interaction |  |
| CDRSB | A1 | 1924.652 | 1937.097 | 0.603 | <0.001 | 0.247 |
|  | A2 | 1889.879 | 1902.324 | 0.364 | <0.001 | 0.242 |
|  | T1 | 1927.757 | 1940.202 | 0.893 | 0.017 | 0.185 |
|  | T2 | 1828.001 | 1840.447 | 0.054 | <0.001 | 0.369 |
|  | T3 | 1860.239 | 1872.684 | 0.286 | <0.001 | 0.308 |
|  | N1 | 1862.556 | 1875.001 | 0.233 | <0.001 | 0.360 |
|  | N2 | 1808.830 | 1821.275 | 0.054 | <0.001 | 0.470 |
|  | N3 | 1933.568 | 1946.013 | 0.704 | 0.004 | 0.191 |
|  | N4 | 1872.254 | 1884.699 | 0.305 | <0.001 | 0.302 |
|  | N5 | 1902.694 | 1915.139 | 0.140 | <0.001 | 0.262 |
| MMSE | A1 | 2182.840 | 2195.221 | 0.461 | <0.001 | 0.239 |
|  | A2 | 2150.440 | 2162.820 | 0.148 | <0.001 | 0.229 |
|  | T1 | 2179.991 | 2192.371 | 0.942 | 0.007 | 0.200 |
|  | T2 | 2062.928 | 2075.309 | 0.001 | <0.001 | 0.413 |
|  | T3 | 2087.062 | 2099.443 | 0.036 | <0.001 | 0.383 |
|  | N1 | 2125.473 | 2137.854 | 0.421 | <0.001 | 0.348 |
|  | N2 | 2103.043 | 2115.424 | 0.216 | <0.001 | 0.378 |
|  | N3 | 2187.584 | 2187.637 | 0.866 | 0.006 | 0.206 |
|  | N4 | 2139.712 | 2152.092 | 0.547 | <0.001 | 0.274 |
|  | N5 | 2177.977 | 2190.358 | 0.937 | 0.001 | 0.219 |

## Supplementary Table 8 Linear-mixed effect model with interactions for longitudinal cognition in CI using AT(N) variants

| Scale | AT(N) Variants | Goodness of fit | | p-value | | | | | | Marginal R2 |
| --- | --- | --- | --- | --- | --- | --- | --- | --- | --- | --- |
|  |  |  |  | Main effect | | | Interaction | | |  |
|  |  | AIC | BIC | A | T | N | A×Time | T×Time | N×Time |  |
| CDRSB | A1T1N1 | 1891.308 | 1903.728 | >0.05 | >0.05 | >0.05 | 0.013 | 0.035 | <0.001 | 0.392 |
|  | A1T1N2 | 1832.512 | 1844.931 | >0.05 | >0.05 | >0.05 | 0.003 | 0.026 | <0.001 | 0.513 |
|  | A1T1N3 | 1926.096 | 1938.516 | >0.05 | >0.05 | >0.05 | <0.001 | <0.001 | <0.001 | 0.307 |
|  | A1T1N4 | 1900.243 | 1912.662 | >0.05 | >0.05 | >0.05 | 0.003 | 0.097 | <0.001 | 0.333 |
|  | A1T1N5 | 1926.837 | 1939.257 | >0.05 | >0.05 | >0.05 | 0.002 | 0.085 | <0.001 | 0.320 |
|  | A1T2N1 | 1839.456 | 1851.876 | >0.05 | >0.05 | >0.05 | 0.065 | 0.000 | 0.001 | 0.447 |
|  | A1T2N2 | 1796.92 | 1809.339 | >0.05 | >0.05 | >0.05 | 0.026 | <0.001 | <0.001 | 0.528 |
|  | A1T2N3 | 1874.023 | 1886.443 | >0.05 | >0.05 | >0.05 | 0.010 | <0.001 | 0.785 | 0.397 |
|  | A1T2N4 | 1831.318 | 1843.737 | >0.05 | >0.05 | >0.05 | 0.055 | 0.001 | <0.001 | 0.421 |
|  | A1T2N5 | 1856.301 | 1868.72 | >0.05 | >0.05 | >0.05 | 0.201 | <0.001 | 0.002 | 0.426 |
|  | A1T3N1 | 1857.729 | 1870.149 | >0.05 | >0.05 | >0.05 | 0.022 | <0.001 | <0.001 | 0.422 |
|  | A1T3N2 | 1804.401 | 1816.821 | >0.05 | >0.05 | >0.05 | 0.009 | <0.001 | <0.001 | 0.522 |
|  | A1T3N3 | 1898.947 | 1911.366 | >0.05 | >0.05 | >0.05 | 0.001 | <0.001 | 0.419 | 0.350 |
|  | A1T3N4 | 1854.859 | 1867.278 | >0.05 | >0.05 | >0.05 | 0.011 | <0.001 | <0.001 | 0.387 |
|  | A1T3N5 | 1872.84 | 1885.26 | >0.05 | >0.05 | >0.05 | 0.013 | <0.001 | <0.001 | 0.401 |
|  | A2T1N1 | 1857.008 | 1869.428 | >0.05 | >0.05 | >0.05 | 0.003 | 0.281 | <0.001 | 0.392 |
|  | A2T1N2 | 1801.805 | 1814.225 | >0.05 | >0.05 | >0.05 | 0.002 | 0.237 | <0.001 | 0.504 |
|  | A2T1N3 | 1904.186 | 1916.606 | >0.05 | >0.05 | >0.05 | <0.001 | <0.001 | <0.001 | 0.277 |
|  | A2T1N4 | 1866.229 | 1878.649 | >0.05 | >0.05 | >0.05 | 0.001 | 0.603 | <0.001 | 0.335 |
|  | A2T1N5 | 1892.541 | 1904.961 | >0.05 | >0.05 | >0.05 | <0.001 | 0.618 | <0.001 | 0.324 |
|  | A2T2N1 | 1811.678 | 1824.097 | >0.05 | >0.05 | >0.05 | 0.240 | <0.001 | <0.001 | 0.437 |
|  | A2T2N2 | 1769.922 | 1782.341 | >0.05 | >0.05 | >0.05 | 0.103 | <0.001 | <0.001 | 0.515 |
|  | A2T2N3 | 1850.581 | 1863 | >0.05 | >0.05 | >0.05 | 0.256 | <0.001 | 0.829 | 0.375 |
|  | A2T2N4 | 1804.506 | 1816.926 | >0.05 | >0.05 | >0.05 | 0.345 | <0.001 | <0.001 | 0.408 |
|  | A2T2N5 | 1830.089 | 1842.508 | >0.05 | >0.05 | >0.05 | 0.269 | <0.001 | 0.001 | 0.411 |
|  | A2T3N1 | 1828.322 | 1840.741 | >0.05 | >0.05 | >0.05 | 0.030 | 0.001 | <0.001 | 0.414 |
|  | A2T3N2 | 1776.534 | 1788.954 | >0.05 | >0.05 | >0.05 | 0.020 | 0.003 | <0.001 | 0.511 |
|  | A2T3N3 | 1877.035 | 1889.455 | >0.05 | >0.05 | >0.05 | 0.021 | <0.001 | 0.899 | 0.326 |
|  | A2T3N4 | 1827.625 | 1840.045 | >0.05 | >0.05 | >0.05 | 0.037 | <0.001 | <0.001 | 0.374 |
|  | A2T3N5 | 1845.981 | 1858.4 | >0.05 | >0.05 | >0.05 | 0.048 | <0.001 | <0.001 | 0.387 |
| MMSE | A1T1N1 | 2140.338 | 2152.692 | >0.05 | >0.05 | >0.05 | 0.003 | 0.006 | <0.001 | 0.392 |
|  | A1T1N2 | 2111.238 | 2123.592 | >0.05 | >0.05 | >0.05 | <0.001 | 0.005 | <0.001 | 0.513 |
|  | A1T1N3 | 2113.816 | 2126.17 | >0.05 | 0.038 | >0.05 | <0.001 | 0.946 | <0.001 | 0.307 |
|  | A1T1N4 | 2154.507 | 2166.862 | >0.05 | >0.05 | >0.05 | 0.001 | 0.020 | <0.001 | 0.333 |
|  | A1T1N5 | 2188.38 | 2200.734 | >0.05 | >0.05 | >0.05 | <0.001 | 0.024 | 0.050 | 0.320 |
|  | A1T2N1 | 2074.676 | 2087.03 | >0.05 | 0.008 | >0.05 | 0.024 | <0.001 | 0.009 | 0.447 |
|  | A1T2N2 | 2056.358 | 2068.712 | >0.05 | 0.012 | >0.05 | 0.010 | <0.001 | <0.001 | 0.528 |
|  | A1T2N3 | 2103.473 | 2115.827 | >0.05 | 0.001 | >0.05 | 0.006 | <0.001 | 0.952 | 0.397 |
|  | A1T2N4 | 2067.429 | 2079.783 | >0.05 | 0.003 | >0.05 | 0.017 | <0.001 | 0.016 | 0.421 |
|  | A1T2N5 | 2097.699 | 2110.054 | >0.05 | 0.002 | >0.05 | 0.008 | <0.001 | 0.824 | 0.426 |
|  | A1T3N1 | 2087.69 | 2100.044 | >0.05 | >0.05 | >0.05 | 0.005 | <0.001 | 0.001 | 0.422 |
|  | A1T3N2 | 2059.178 | 2071.533 | >0.05 | >0.05 | >0.05 | 0.002 | <0.001 | <0.001 | 0.522 |
|  | A1T3N3 | 2121.141 | 2133.496 | >0.05 | 0.037 | >0.05 | <0.001 | <0.001 | 0.656 | 0.350 |
|  | A1T3N4 | 2085.427 | 2097.782 | >0.05 | >0.05 | >0.05 | 0.002 | <0.001 | 0.014 | 0.387 |
|  | A1T3N5 | 2110.259 | 2122.613 | >0.05 | >0.05 | >0.05 | 0.002 | <0.001 | 0.104 | 0.401 |
|  | A2T1N1 | 2109.013 | 2121.367 | >0.05 | >0.05 | >0.05 | 0.001 | 0.105 | <0.001 | 0.392 |
|  | A2T1N2 | 2084.056 | 2096.41 | >0.05 | >0.05 | >0.05 | <0.001 | 0.111 | <0.001 | 0.504 |
|  | A2T1N3 | 2169.977 | 2182.332 | >0.05 | >0.05 | >0.05 | <0.001 | 0.185 | 0.112 | 0.277 |
|  | A2T1N4 | 2125.429 | 2137.783 | >0.05 | >0.05 | >0.05 | <0.001 | 0.261 | <0.001 | 0.335 |
|  | A2T1N5 | 2160.238 | 2172.593 | >0.05 | >0.05 | >0.05 | <0.001 | 0.307 | 0.018 | 0.324 |
|  | A2T2N1 | 2049.498 | 2061.852 | >0.05 | 0.026 | >0.05 | 0.314 | <0.001 | 0.002 | 0.437 |
|  | A2T2N2 | 2033.136 | 2045.49 | >0.05 | 0.037 | >0.05 | 0.200 | <0.001 | <0.001 | 0.515 |
|  | A2T2N3 | 2082.614 | 2094.968 | >0.05 | 0.003 | >0.05 | 0.334 | <0.001 | 0.647 | 0.375 |
|  | A2T2N4 | 2043.464 | 2055.818 | >0.05 | 0.008 | >0.05 | 0.425 | <0.001 | 0.006 | 0.408 |
|  | A2T2N5 | 2075.719 | 2088.074 | >0.05 | 0.009 | >0.05 | 0.396 | <0.001 | 0.482 | 0.411 |
|  | A2T3N1 | 2062.855 | 2075.209 | >0.05 | >0.05 | >0.05 | 0.045 | <0.001 | <0.001 | 0.414 |
|  | A2T3N2 | 2036.75 | 2049.104 | >0.05 | >0.05 | >0.05 | 0.036 | <0.001 | <0.001 | 0.511 |
|  | A2T3N3 | 2103.418 | 2115.772 | >0.05 | >0.05 | >0.05 | 0.036 | <0.001 | 0.811 | 0.326 |
|  | A2T3N4 | 2063.281 | 2075.636 | >0.05 | >0.05 | >0.05 | 0.052 | <0.001 | 0.004 | 0.374 |
|  | A2T3N5 | 2088.816 | 2101.171 | >0.05 | >0.05 | >0.05 | 0.080 | <0.001 | 0.036 | 0.387 |

## Supplementary Table 9 Linear-mixed effect model for longitudinal cognition using time as a covariant

| Scale | CDRSB | | | | MMSE | | | |
| --- | --- | --- | --- | --- | --- | --- | --- | --- |
| Group | AT(N) variants | Marginal R2 | AT(N) variants | Marginal R2 | AT(N) variants | Marginal R2 | AT(N) variants | Marginal R2 |
| Linear-mixed effect model for longitudinal cognition using single AT(N) biomarkers (time as a covariant) | | | | | | | | |
| CU | A1 | 0.040 | N1 | 0.042 | A1 | 0.079 | **N1** | 0.094 |
|  | A2 | 0.041 | **N2** | 0.054 | A2 | 0.080 | N2 | 0.085 |
|  | T1 | 0.043 | N3 | 0.043 | T1 | 0.084 | N3 | 0.083 |
|  | T2 | 0.041 | N4 | 0.040 | T2 | 0.080 | N4 | 0.088 |
|  | T3 | 0.043 | N5 | 0.041 | T3 | 0.081 | N5 | 0.080 |
| CI | A1 | 0.153 | N1 | 0.161 | A1 | 0.164 | N1 | 0.177 |
|  | A2 | 0.150 | N2 | 0.163 | A2 | 0.161 | N2 | 0.174 |
|  | T1 | 0.149 | N3 | 0.148 | T1 | 0.169 | N3 | 0.170 |
|  | T2 | 0.165 | N4 | 0.157 | T2 | 0.175 | N4 | 0.172 |
|  | T3 | 0.162 | N5 | 0.147 | T3 | 0.180 | N5 | 0.170 |
| Linear-mixed effect model for longitudinal cognition using AT(N) combinations (time as a covariant) | | | | | | | | |
| CU | A1T1N1 | 0.045 | A2T1N1 | 0.045 | A1T1N1 | 0.100 | **A2T1N1** | 0.103 |
|  | A1T1N2 | 0.056 | A2T1N2 | 0.056 | A1T1N2 | 0.090 | A2T1N2 | 0.093 |
|  | A1T1N3 | 0.043 | A2T1N3 | 0.043 | A1T1N3 | 0.085 | A2T1N3 | 0.091 |
|  | A1T1N4 | 0.043 | A2T1N4 | 0.043 | A1T1N4 | 0.092 | A2T1N4 | 0.095 |
|  | A1T1N5 | 0.044 | A2T1N5 | 0.044 | A1T1N5 | 0.084 | A2T1N5 | 0.088 |
|  | A1T2N1 | 0.042 | A2T2N1 | 0.043 | A1T2N1 | 0.094 | A2T2N1 | 0.094 |
|  | A1T2N2 | 0.054 | A2T2N2 | 0.055 | A1T2N2 | 0.086 | A2T2N2 | 0.086 |
|  | A1T2N3 | 0.045 | A2T2N3 | 0.045 | A1T2N3 | 0.086 | A2T2N3 | 0.087 |
|  | A1T2N4 | 0.041 | A2T2N4 | 0.042 | A1T2N4 | 0.090 | A2T2N4 | 0.089 |
|  | A1T2N5 | 0.041 | A2T2N5 | 0.042 | A1T2N5 | 0.081 | A2T2N5 | 0.081 |
|  | A1T3N1 | 0.045 | A2T3N1 | 0.047 | A1T3N1 | 0.096 | A2T3N1 | 0.096 |
|  | **A1T3N2** | 0.056 | A2T3N2 | 0.057 | A1T3N2 | 0.087 | A2T3N2 | 0.087 |
|  | A1T3N3 | 0.048 | A2T3N3 | 0.048 | A1T3N3 | 0.086 | A2T3N3 | 0.087 |
|  | A1T3N4 | 0.043 | A2T3N4 | 0.045 | A1T3N4 | 0.091 | A2T3N4 | 0.091 |
|  | A1T3N5 | 0.043 | A2T3N5 | 0.045 | A1T3N5 | 0.082 | A2T3N5 | 0.082 |
| CI | A1T1N1 | 0.163 | A2T1N1 | 0.162 | A1T1N1 | 0.186 | A2T1N1 | 0.185 |
|  | A1T1N2 | 0.166 | A2T1N2 | 0.165 | A1T1N2 | 0.185 | A2T1N2 | 0.183 |
|  | A1T1N3 | 0.155 | A2T1N3 | 0.154 | A1T1N3 | 0.175 | A2T1N3 | 0.170 |
|  | A1T1N4 | 0.158 | A2T1N4 | 0.157 | A1T1N4 | 0.177 | A2T1N4 | 0.176 |
|  | A1T1N5 | 0.154 | A2T1N5 | 0.151 | A1T1N5 | 0.180 | A2T1N5 | 0.178 |
|  | A1T2N1 | 0.170 | A2T2N1 | 0.168 | A1T2N1 | 0.184 | A2T2N1 | 0.183 |
|  | **A1T2N2** | 0.171 | A2T2N2 | 0.169 | A1T2N2 | 0.184 | A2T2N2 | 0.182 |
|  | A1T2N3 | 0.166 | A2T2N3 | 0.164 | A1T2N3 | 0.181 | A2T2N3 | 0.179 |
|  | A1T2N4 | 0.168 | A2T2N4 | 0.166 | A1T2N4 | 0.181 | A2T2N4 | 0.180 |
|  | A1T2N5 | 0.167 | A2T2N5 | 0.165 | A1T2N5 | 0.182 | A2T2N5 | 0.180 |
|  | A1T3N1 | 0.168 | A2T3N1 | 0.166 | A1T3N1 | 0.189 | A2T3N1 | 0.187 |
|  | A1T3N2 | 0.170 | A2T3N2 | 0.168 | **A1T3N2** | 0.191 | A2T3N2 | 0.188 |
|  | A1T3N3 | 0.164 | A2T3N3 | 0.162 | A1T3N3 | 0.184 | A2T3N3 | 0.182 |
|  | A1T3N4 | 0.166 | A2T3N4 | 0.164 | A1T3N4 | 0.186 | A2T3N4 | 0.185 |
|  | A1T3N5 | 0.165 | A2T3N5 | 0.162 | A1T3N5 | 0.189 | A2T3N5 | 0.187 |

## Supplementary Table 10 Sensitivity analysis for AT(N) prevalence using alternative cutoffs

| AT(N) variants | category | 90% sensitivity for AD | | | | mean ± 2 SD from Aβ-negative CU controls | | | |
| --- | --- | --- | --- | --- | --- | --- | --- | --- | --- |
|  |  | CU | CI | MCI | AD | CU | CI | MCI | AD |
| A1T1 | A-T- | 33.5% | 14.9% | 18.8% | 5.0% | 90.5% | 71.6% | 78.2% | 55.0% |
|  | A-T+ | 33.0% | 22.7% | 29.7% | 5.0% | 5.0% | 14.2% | 10.9% | 22.5% |
|  | A+T- | 12.0% | 12.8% | 15.8% | 5.0% | 4.5% | 12.8% | 9.9% | 20.0% |
|  | A+T+ | 21.5% | 49.6% | 35.6% | 85.0% | 0.0% | 1.4% | 1.0% | 2.5% |
| A1T2 | A-T- | 49.0% | 24.8% | 33.7% | 2.5% | 91.5% | 51.8% | 65.3% | 17.5% |
|  | A-T+ | 17.5% | 12.8% | 14.9% | 7.5% | 4.0% | 34.0% | 23.8% | 60.0% |
|  | A+T- | 19.0% | 14.2% | 16.8% | 7.5% | 3.5% | 6.4% | 6.9% | 5.0% |
|  | A+T+ | 14.5% | 48.2% | 34.7% | 82.5% | 1.0% | 7.8% | 4.0% | 17.5% |
| A1T3 | A-T- | 36.0% | 15.6% | 20.8% | 2.5% | 89.0% | 61.0% | 74.3% | 27.5% |
|  | A-T+ | 30.5% | 22.0% | 27.7% | 7.5% | 6.5% | 24.8% | 14.9% | 50.0% |
|  | A+T- | 17.5% | 9.2% | 9.9% | 7.5% | 3.5% | 7.8% | 8.9% | 5.0% |
|  | A+T+ | 16.0% | 53.2% | 41.6% | 82.5% | 1.0% | 6.4% | 2.0% | 17.5% |
| A2T1 | A-T- | 42.5% | 20.6% | 26.7% | 5.0% | 84.5% | 50.4% | 62.4% | 20.0% |
|  | A-T+ | 36.0% | 17.7% | 22.8% | 5.0% | 1.5% | 6.4% | 5.9% | 7.5% |
|  | A+T- | 3.0% | 7.1% | 7.9% | 5.0% | 10.5% | 34.0% | 25.7% | 55.0% |
|  | A+T+ | 18.5% | 54.6% | 42.6% | 85.0% | 3.5% | 9.2% | 5.9% | 17.5% |
| A2T2 | A-T- | 59.5% | 25.5% | 34.7% | 2.5% | 84.0% | 44.7% | 57.4% | 12.5% |
|  | A-T+ | 19.0% | 12.8% | 14.9% | 7.5% | 2.0% | 12.1% | 10.9% | 15.0% |
|  | A+T- | 8.5% | 13.5% | 15.8% | 7.5% | 11.0% | 13.5% | 14.9% | 10.0% |
|  | A+T+ | 13.0% | 48.2% | 34.7% | 82.5% | 3.0% | 29.8% | 16.8% | 62.5% |
| A2T3 | A-T- | 46.0% | 17.7% | 23.8% | 2.5% | 81.5% | 47.5% | 61.4% | 12.5% |
|  | A-T+ | 32.5% | 20.6% | 25.7% | 7.5% | 4.5% | 9.2% | 6.9% | 15.0% |
|  | A+T- | 7.5% | 7.1% | 6.9% | 7.5% | 11.0% | 21.3% | 21.8% | 20.0% |
|  | A+T+ | 14.0% | 54.6% | 43.6% | 82.5% | 3.0% | 22.0% | 9.9% | 52.5% |
| A1T1N1 | A-T-N- | 22.0% | 7.1% | 8.9% | 2.5% | 87.5% | 53.2% | 63.4% | 27.5% |
|  | A-T-N+ | 11.5% | 7.8% | 9.9% | 2.5% | 3.0% | 18.4% | 14.9% | 27.5% |
|  | A-T+N- | 20.5% | 9.2% | 11.9% | 2.5% | 5.0% | 7.8% | 8.9% | 5.0% |
|  | A-T+N+ | 12.5% | 13.5% | 17.8% | 2.5% | 0.0% | 6.4% | 2.0% | 17.5% |
|  | A+T-N- | 7.5% | 3.5% | 5.0% | 0.0% | 3.5% | 7.1% | 6.9% | 7.5% |
|  | A+T-N+ | 4.5% | 9.2% | 10.9% | 5.0% | 1.0% | 5.7% | 3.0% | 12.5% |
|  | A+T+N- | 15.0% | 8.5% | 8.9% | 7.5% | 0.0% | 1.4% | 1.0% | 2.5% |
|  | A+T+N+ | 6.5% | 41.1% | 26.7% | 77.5% | 0.0% | 0.0% | 0.0% | 0.0% |
| A1T1N2 | A-T-N- | 22.6% | 9.2% | 11.9% | 2.5% | 90.5% | 71.6% | 78.2% | 55.0% |
|  | A-T-N+ | 11.1% | 5.7% | 6.9% | 2.5% | 0.0% | 0.0% | 0.0% | 0.0% |
|  | A-T+N- | 22.1% | 9.2% | 11.9% | 2.5% | 5.0% | 14.2% | 10.9% | 22.5% |
|  | A-T+N+ | 11.1% | 13.5% | 17.8% | 2.5% | 0.0% | 0.0% | 0.0% | 0.0% |
|  | A+T-N- | 8.5% | 4.3% | 5.9% | 0.0% | 4.5% | 12.8% | 9.9% | 20.0% |
|  | A+T-N+ | 3.5% | 8.5% | 9.9% | 5.0% | 0.0% | 0.0% | 0.0% | 0.0% |
|  | A+T+N- | 15.1% | 9.2% | 9.9% | 7.5% | 0.0% | 1.4% | 1.0% | 2.5% |
|  | A+T+N+ | 6.0% | 40.4% | 25.7% | 77.5% | 0.0% | 0.0% | 0.0% | 0.0% |
| A1T1N3 | A-T-N- | 29.0% | 10.6% | 12.9% | 5.0% | 90.0% | 70.2% | 77.2% | 52.5% |
|  | A-T-N+ | 4.5% | 4.3% | 0.0% | 0.0% | .5% | 1.4% | 1.0% | 2.5% |
|  | A-T+N- | 1.0% | 1.4% | 5.9% | 0.0% | 1.0% | 2.8% | 3.0% | 2.5% |
|  | A-T+N+ | 32.0% | 21.3% | 1.0% | 2.5% | 4.0% | 11.3% | 7.9% | 20.0% |
|  | A+T-N- | 11.5% | 12.8% | 28.7% | 2.5% | 4.5% | 12.8% | 9.9% | 20.0% |
|  | A+T-N+ | .5% | 0.0% | 15.8% | 5.0% | 0.0% | 0.0% | 0.0% | 0.0% |
|  | A+T+N- | 1.5% | .7% | 1.0% | 0.0% | 0.0% | 0.0% | 0.0% | 0.0% |
|  | A+T+N+ | 20.0% | 48.9% | 34.7% | 85.0% | 0.0% | 1.4% | 1.0% | 2.5% |
| A1T1N4 | A-T-N- | 24.8% | 8.0% | 11.1% | 0.0% | 88.7% | 56.9% | 66.7% | 31.6% |
|  | A-T-N+ | 8.5% | 7.3% | 8.1% | 5.3% | 1.4% | 15.3% | 11.1% | 26.3% |
|  | A-T+N- | 29.8% | 10.9% | 15.2% | 0.0% | 5.7% | 9.5% | 10.1% | 7.9% |
|  | A-T+N+ | 9.2% | 11.7% | 14.1% | 5.3% | 0.0% | 5.1% | 1.0% | 15.8% |
|  | A+T-N- | 5.7% | 5.1% | 7.1% | 0.0% | 4.3% | 7.3% | 7.1% | 7.9% |
|  | A+T-N+ | 4.3% | 7.3% | 9.1% | 2.6% | 0.0% | 4.4% | 3.0% | 7.9% |
|  | A+T+N- | 12.8% | 12.4% | 13.1% | 10.5% | 0.0% | .7% | 1.0% | 0.0% |
|  | A+T+N+ | 5.0% | 37.2% | 22.2% | 76.3% | 0.0% | .7% | 0.0% | 2.6% |
| A1T1N5 | A-T-N- | 14.5% | 6.0% | 7.2% | 3.0% | 82.6% | 65.5% | 73.5% | 45.5% |
|  | A-T-N+ | 18.1% | 9.5% | 12.0% | 3.0% | 7.2% | 9.5% | 8.4% | 12.1% |
|  | A-T+N- | 20.3% | 9.5% | 13.3% | 0.0% | 4.3% | 9.5% | 6.0% | 18.2% |
|  | A-T+N+ | 18.8% | 14.7% | 18.1% | 6.1% | 1.4% | 4.3% | 3.6% | 6.1% |
|  | A+T-N- | 4.3% | 2.6% | 3.6% | 0.0% | 4.3% | 9.5% | 8.4% | 12.1% |
|  | A+T-N+ | 5.8% | 9.5% | 12.0% | 3.0% | 0.0% | .9% | 0.0% | 3.0% |
|  | A+T+N- | 5.8% | 10.3% | 9.6% | 12.1% | 0.0% | .9% | 0.0% | 3.0% |
|  | A+T+N+ | 12.3% | 37.9% | 24.1% | 72.7% | 0.0% | 0.0% | 0.0% | 0.0% |
| A1T2N1 | A-T-N- | 33.5% | 14.2% | 18.8% | 2.5% | 88.5% | 46.8% | 59.4% | 15.0% |
|  | A-T-N+ | 15.5% | 10.6% | 14.9% | 0.0% | 3.0% | 5.0% | 5.9% | 2.5% |
|  | A-T+N- | 9.0% | 2.1% | 2.0% | 2.5% | 4.0% | 14.2% | 12.9% | 17.5% |
|  | A-T+N+ | 8.5% | 10.6% | 12.9% | 5.0% | 0.0% | 19.9% | 10.9% | 42.5% |
|  | A+T-N- | 14.0% | 5.0% | 6.9% | 0.0% | 2.5% | 3.5% | 4.0% | 2.5% |
|  | A+T-N+ | 5.0% | 9.2% | 9.9% | 7.5% | 1.0% | 2.8% | 3.0% | 2.5% |
|  | A+T+N- | 8.5% | 7.1% | 6.9% | 7.5% | 1.0% | 5.0% | 4.0% | 7.5% |
|  | A+T+N+ | 6.0% | 41.1% | 27.7% | 75.0% | 0.0% | 2.8% | 0.0% | 10.0% |
| A1T2N2 | A-T-N- | 34.2% | 15.6% | 20.8% | 2.5% | 91.5% | 51.8% | 65.3% | 17.5% |
|  | A-T-N+ | 15.1% | 9.2% | 12.9% | 0.0% | 0.0% | 0.0% | 0.0% | 0.0% |
|  | A-T+N- | 10.6% | 2.8% | 3.0% | 2.5% | 4.0% | 34.0% | 23.8% | 60.0% |
|  | A-T+N+ | 7.0% | 9.9% | 11.9% | 5.0% | 0.0% | 0.0% | 0.0% | 0.0% |
|  | A+T-N- | 14.6% | 5.7% | 7.9% | 0.0% | 3.5% | 6.4% | 6.9% | 5.0% |
|  | A+T-N+ | 4.0% | 8.5% | 8.9% | 7.5% | 0.0% | 0.0% | 0.0% | 0.0% |
|  | A+T+N- | 9.0% | 7.8% | 7.9% | 7.5% | 1.0% | 7.8% | 4.0% | 17.5% |
|  | A+T+N+ | 5.5% | 40.4% | 26.7% | 75.0% | 0.0% | 0.0% | 0.0% | 0.0% |
| A1T2N3 | A-T-N- | 23.5% | 8.5% | 10.9% | 2.5% | 88.5% | 48.2% | 61.4% | 15.0% |
|  | A-T-N+ | 25.5% | 16.3% | 22.8% | 0.0% | 3.0% | 3.5% | 4.0% | 2.5% |
|  | A-T+N- | 6.5% | 3.5% | 3.0% | 5.0% | 2.5% | 24.8% | 18.8% | 40.0% |
|  | A-T+N+ | 11.0% | 9.2% | 11.9% | 2.5% | 1.5% | 9.2% | 5.0% | 20.0% |
|  | A+T-N- | 10.0% | 5.7% | 7.9% | 0.0% | 3.5% | 6.4% | 6.9% | 5.0% |
|  | A+T-N+ | 9.0% | 8.5% | 8.9% | 7.5% | 0.0% | 0.0% | 0.0% | 0.0% |
|  | A+T+N- | 3.0% | 7.8% | 8.9% | 5.0% | 1.0% | 6.4% | 3.0% | 15.0% |
|  | A+T+N+ | 11.5% | 40.4% | 25.7% | 77.5% | 0.0% | 1.4% | 1.0% | 2.5% |
| A1T2N4 | A-T-N- | 39.0% | 13.9% | 19.2% | 0.0% | 88.7% | 46.0% | 57.6% | 15.8% |
|  | A-T-N+ | 11.3% | 10.9% | 14.1% | 2.6% | 1.4% | 6.6% | 8.1% | 2.6% |
|  | A-T+N- | 15.6% | 5.1% | 7.1% | 0.0% | 5.7% | 20.4% | 19.2% | 23.7% |
|  | A-T+N+ | 6.4% | 8.0% | 8.1% | 7.9% | 0.0% | 13.9% | 4.0% | 39.5% |
|  | A+T-N- | 8.5% | 4.4% | 6.1% | 0.0% | 2.8% | 3.6% | 4.0% | 2.6% |
|  | A+T-N+ | 5.0% | 9.5% | 11.1% | 5.3% | 0.0% | 2.2% | 3.0% | 0.0% |
|  | A+T+N- | 9.9% | 13.1% | 14.1% | 10.5% | 1.4% | 4.4% | 4.0% | 5.3% |
|  | A+T+N+ | 4.3% | 35.0% | 20.2% | 73.7% | 0.0% | 2.9% | 0.0% | 10.5% |
| A1T2N5 | A-T-N- | 24.6% | 11.2% | 14.5% | 3.0% | 81.2% | 48.3% | 60.2% | 18.2% |
|  | A-T-N+ | 25.4% | 13.8% | 19.3% | 0.0% | 8.7% | 5.2% | 6.0% | 3.0% |
|  | A-T+N- | 10.1% | 4.3% | 6.0% | 0.0% | 5.8% | 26.7% | 19.3% | 45.5% |
|  | A-T+N+ | 11.6% | 10.3% | 10.8% | 9.1% | 0.0% | 8.6% | 6.0% | 15.2% |
|  | A+T-N- | 4.3% | 3.4% | 4.8% | 0.0% | 2.9% | 6.0% | 7.2% | 3.0% |
|  | A+T-N+ | 9.4% | 11.2% | 13.3% | 6.1% | 0.0% | 0.0% | 0.0% | 0.0% |
|  | A+T+N- | 5.8% | 9.5% | 8.4% | 12.1% | 1.4% | 4.3% | 1.2% | 12.1% |
|  | A+T+N+ | 8.7% | 36.2% | 22.9% | 69.7% | 0.0% | .9% | 0.0% | 3.0% |
| A1T3N1 | A-T-N- | 24.5% | 8.5% | 10.9% | 2.5% | 86.0% | 52.5% | 64.4% | 22.5% |
|  | A-T-N+ | 11.5% | 7.1% | 9.9% | 0.0% | 3.0% | 8.5% | 9.9% | 5.0% |
|  | A-T+N- | 18.0% | 7.8% | 9.9% | 2.5% | 6.5% | 8.5% | 7.9% | 10.0% |
|  | A-T+N+ | 12.5% | 14.2% | 17.8% | 5.0% | 0.0% | 16.3% | 6.9% | 40.0% |
|  | A+T-N- | 13.0% | 5.0% | 5.9% | 2.5% | 2.5% | 5.0% | 5.9% | 2.5% |
|  | A+T-N+ | 4.5% | 4.3% | 4.0% | 5.0% | 1.0% | 2.8% | 3.0% | 2.5% |
|  | A+T+N- | 9.5% | 7.1% | 7.9% | 5.0% | 1.0% | 3.5% | 2.0% | 7.5% |
|  | A+T+N+ | 6.5% | 46.1% | 33.7% | 77.5% | 0.0% | 2.8% | 0.0% | 10.0% |
| A1T3N2 | A-T-N- | 26.6% | 9.2% | 11.9% | 2.5% | 88.9% | 61.0% | 74.3% | 27.5% |
|  | A-T-N+ | 9.5% | 6.4% | 8.9% | 0.0% | 0.0% | 0.0% | 0.0% | 0.0% |
|  | A-T+N- | 18.1% | 9.2% | 11.9% | 2.5% | 6.5% | 24.8% | 14.9% | 50.0% |
|  | A-T+N+ | 12.6% | 12.8% | 15.8% | 5.0% | 0.0% | 0.0% | 0.0% | 0.0% |
|  | A+T-N- | 13.1% | 4.3% | 5.9% | 0.0% | 3.5% | 7.8% | 8.9% | 5.0% |
|  | A+T-N+ | 4.0% | 5.0% | 4.0% | 7.5% | 0.0% | 0.0% | 0.0% | 0.0% |
|  | A+T+N- | 10.6% | 9.2% | 9.9% | 7.5% | 1.0% | 6.4% | 2.0% | 17.5% |
|  | A+T+N+ | 5.5% | 44.0% | 31.7% | 75.0% | 0.0% | 0.0% | 0.0% | 0.0% |
| A1T3N3 | A-T-N- | 19.0% | 5.7% | 6.9% | 2.5% | 85.5% | 56.0% | 68.3% | 25.0% |
|  | A-T-N+ | 17.0% | 9.9% | 13.9% | 0.0% | 3.5% | 5.0% | 5.9% | 2.5% |
|  | A-T+N- | 11.0% | 6.4% | 6.9% | 5.0% | 5.5% | 17.0% | 11.9% | 30.0% |
|  | A-T+N+ | 19.5% | 15.6% | 20.8% | 2.5% | 1.0% | 7.8% | 3.0% | 20.0% |
|  | A+T-N- | 9.0% | 2.8% | 4.0% | 0.0% | 3.5% | 7.8% | 8.9% | 5.0% |
|  | A+T-N+ | 8.5% | 6.4% | 5.9% | 7.5% | 0.0% | 0.0% | 0.0% | 0.0% |
|  | A+T+N- | 4.0% | 10.6% | 12.9% | 5.0% | 1.0% | 5.0% | 1.0% | 15.0% |
|  | A+T+N+ | 12.0% | 42.6% | 28.7% | 77.5% | 0.0% | 1.4% | 1.0% | 2.5% |
| A1T3N4 | A-T-N- | 28.4% | 7.3% | 10.1% | 0.0% | 85.8% | 52.6% | 63.6% | 23.7% |
|  | A-T-N+ | 7.8% | 8.0% | 10.1% | 2.6% | 1.4% | 8.8% | 10.1% | 5.3% |
|  | A-T+N- | 26.2% | 11.7% | 16.2% | 0.0% | 8.5% | 13.9% | 13.1% | 15.8% |
|  | A-T+N+ | 9.9% | 10.9% | 12.1% | 7.9% | 0.0% | 11.7% | 2.0% | 36.8% |
|  | A+T-N- | 7.1% | 3.6% | 4.0% | 2.6% | 3.5% | 5.1% | 6.1% | 2.6% |
|  | A+T-N+ | 3.5% | 5.1% | 6.1% | 2.6% | 0.0% | 2.2% | 3.0% | 0.0% |
|  | A+T+N- | 11.3% | 13.9% | 16.2% | 7.9% | .7% | 2.9% | 2.0% | 5.3% |
|  | A+T+N+ | 5.7% | 39.4% | 25.3% | 76.3% | 0.0% | 2.9% | 0.0% | 10.5% |
| A1T3N5 | A-T-N- | 15.2% | 6.9% | 8.4% | 3.0% | 79.0% | 56.9% | 67.5% | 30.3% |
|  | A-T-N+ | 20.3% | 6.0% | 8.4% | 0.0% | 8.0% | 6.0% | 7.2% | 3.0% |
|  | A-T+N- | 19.6% | 8.6% | 12.0% | 0.0% | 8.0% | 18.1% | 12.0% | 33.3% |
|  | A-T+N+ | 16.7% | 18.1% | 21.7% | 9.1% | .7% | 7.8% | 4.8% | 15.2% |
|  | A+T-N- | 4.3% | 2.6% | 3.6% | 0.0% | 3.6% | 6.9% | 8.4% | 3.0% |
|  | A+T-N+ | 6.5% | 6.9% | 7.2% | 6.1% | 0.0% | 0.0% | 0.0% | 0.0% |
|  | A+T+N- | 5.8% | 10.3% | 9.6% | 12.1% | .7% | 3.4% | 0.0% | 12.1% |
|  | A+T+N+ | 11.6% | 40.5% | 28.9% | 69.7% | 0.0% | .9% | 0.0% | 3.0% |
| A2T1N1 | A-T-N- | 27.5% | 9.2% | 11.9% | 2.5% | 81.5% | 38.3% | 49.5% | 10.0% |
|  | A-T-N+ | 15.0% | 11.3% | 14.9% | 2.5% | 3.0% | 12.1% | 12.9% | 10.0% |
|  | A-T+N- | 23.0% | 6.4% | 7.9% | 2.5% | 1.5% | 4.3% | 5.9% | 0.0% |
|  | A-T+N+ | 13.0% | 11.3% | 14.9% | 2.5% | 0.0% | 2.1% | 0.0% | 7.5% |
|  | A+T-N- | 2.0% | 1.4% | 2.0% | 0.0% | 9.5% | 22.0% | 20.8% | 25.0% |
|  | A+T-N+ | 1.0% | 5.7% | 5.9% | 5.0% | 1.0% | 12.1% | 5.0% | 30.0% |
|  | A+T+N- | 12.5% | 11.3% | 12.9% | 7.5% | 3.5% | 5.0% | 4.0% | 7.5% |
|  | A+T+N+ | 6.0% | 43.3% | 29.7% | 77.5% | 0.0% | 4.3% | 2.0% | 10.0% |
| A2T1N2 | A-T-N- | 29.1% | 12.8% | 16.8% | 2.5% | 84.4% | 50.4% | 62.4% | 20.0% |
|  | A-T-N+ | 13.6% | 7.8% | 9.9% | 2.5% | 0.0% | 0.0% | 0.0% | 0.0% |
|  | A-T+N- | 24.6% | 7.1% | 8.9% | 2.5% | 1.5% | 6.4% | 5.9% | 7.5% |
|  | A-T+N+ | 11.1% | 10.6% | 13.9% | 2.5% | 0.0% | 0.0% | 0.0% | 0.0% |
|  | A+T-N- | 2.0% | .7% | 1.0% | 0.0% | 10.6% | 34.0% | 25.7% | 55.0% |
|  | A+T-N+ | 1.0% | 6.4% | 6.9% | 5.0% | 0.0% | 0.0% | 0.0% | 0.0% |
|  | A+T+N- | 12.6% | 11.3% | 12.9% | 7.5% | 3.5% | 9.2% | 5.9% | 17.5% |
|  | A+T+N+ | 6.0% | 43.3% | 29.7% | 77.5% | 0.0% | 0.0% | 0.0% | 0.0% |
| A2T1N3 | A-T-N- | 37.5% | 16.3% | 20.8% | 5.0% | 84.0% | 49.6% | 61.4% | 20.0% |
|  | A-T-N+ | 5.0% | 4.3% | 5.9% | 0.0% | .5% | .7% | 1.0% | 0.0% |
|  | A-T+N- | 2.0% | 1.4% | 1.0% | 2.5% | 0.0% | .7% | 1.0% | 0.0% |
|  | A-T+N+ | 34.0% | 16.3% | 21.8% | 2.5% | 1.5% | 5.7% | 5.0% | 7.5% |
|  | A+T-N- | 3.0% | 7.1% | 7.9% | 5.0% | 10.5% | 33.3% | 25.7% | 52.5% |
|  | A+T-N+ | 0.0% | 0.0% | 0.0% | 0.0% | 0.0% | .7% | 0.0% | 2.5% |
|  | A+T+N- | .5% | .7% | 1.0% | 0.0% | 1.0% | 2.1% | 2.0% | 2.5% |
|  | A+T+N+ | 18.0% | 53.9% | 41.6% | 85.0% | 2.5% | 7.1% | 4.0% | 15.0% |
| A2T1N4 | A-T-N- | 29.1% | 10.9% | 15.2% | 0.0% | 81.6% | 41.6% | 53.5% | 10.5% |
|  | A-T-N+ | 10.6% | 10.2% | 12.1% | 5.3% | 1.4% | 8.8% | 9.1% | 7.9% |
|  | A-T+N- | 30.5% | 7.3% | 10.1% | 0.0% | 2.1% | 5.1% | 6.1% | 2.6% |
|  | A-T+N+ | 7.8% | 10.2% | 12.1% | 5.3% | 0.0% | 1.5% | 0.0% | 5.3% |
|  | A+T-N- | 1.4% | 2.2% | 3.0% | 0.0% | 11.3% | 22.6% | 20.2% | 28.9% |
|  | A+T-N+ | 2.1% | 4.4% | 5.1% | 2.6% | 0.0% | 10.9% | 5.1% | 26.3% |
|  | A+T+N- | 12.1% | 16.1% | 18.2% | 10.5% | 3.5% | 5.1% | 5.1% | 5.3% |
|  | A+T+N+ | 6.4% | 38.7% | 24.2% | 76.3% | 0.0% | 4.4% | 1.0% | 13.2% |
| A2T1N5 | A-T-N- | 17.4% | 8.6% | 10.8% | 3.0% | 77.5% | 47.4% | 59.0% | 18.2% |
|  | A-T-N+ | 21.7% | 12.9% | 16.9% | 3.0% | 5.1% | 5.2% | 6.0% | 3.0% |
|  | A-T+N- | 18.8% | 6.9% | 9.6% | 0.0% | 1.4% | 4.3% | 3.6% | 6.1% |
|  | A-T+N+ | 19.6% | 11.2% | 13.3% | 6.1% | .7% | 2.6% | 2.4% | 3.0% |
|  | A+T-N- | 1.4% | 0.0% | 0.0% | 0.0% | 9.4% | 27.6% | 22.9% | 39.4% |
|  | A+T-N+ | 2.2% | 6.0% | 7.2% | 3.0% | 2.2% | 5.2% | 2.4% | 12.1% |
|  | A+T+N- | 7.2% | 12.9% | 13.3% | 12.1% | 2.9% | 6.0% | 2.4% | 15.2% |
|  | A+T+N+ | 11.6% | 41.4% | 28.9% | 72.7% | .7% | 1.7% | 1.2% | 3.0% |
| A2T2N1 | A-T-N- | 41.5% | 13.5% | 17.8% | 2.5% | 81.0% | 39.0% | 50.5% | 10.0% |
|  | A-T-N+ | 18.0% | 12.1% | 16.8% | 0.0% | 3.0% | 5.7% | 6.9% | 2.5% |
|  | A-T+N- | 9.0% | 2.1% | 2.0% | 2.5% | 2.0% | 3.5% | 5.0% | 0.0% |
|  | A-T+N+ | 10.0% | 10.6% | 12.9% | 5.0% | 0.0% | 8.5% | 5.9% | 15.0% |
|  | A+T-N- | 6.0% | 5.7% | 7.9% | 0.0% | 10.0% | 11.3% | 12.9% | 7.5% |
|  | A+T-N+ | 2.5% | 7.8% | 7.9% | 7.5% | 1.0% | 2.1% | 2.0% | 2.5% |
|  | A+T+N- | 8.5% | 7.1% | 6.9% | 7.5% | 3.0% | 15.6% | 11.9% | 25.0% |
|  | A+T+N+ | 4.5% | 41.1% | 27.7% | 75.0% | 0.0% | 14.2% | 5.0% | 37.5% |
| A2T2N2 | A-T-N- | 42.7% | 17.0% | 22.8% | 2.5% | 83.9% | 44.7% | 57.4% | 12.5% |
|  | A-T-N+ | 16.6% | 8.5% | 11.9% | 0.0% | 0.0% | 0.0% | 0.0% | 0.0% |
|  | A-T+N- | 11.1% | 2.8% | 3.0% | 2.5% | 2.0% | 12.1% | 10.9% | 15.0% |
|  | A-T+N+ | 8.0% | 9.9% | 11.9% | 5.0% | 0.0% | 0.0% | 0.0% | 0.0% |
|  | A+T-N- | 6.0% | 4.3% | 5.9% | 0.0% | 11.1% | 13.5% | 14.9% | 10.0% |
|  | A+T-N+ | 2.5% | 9.2% | 9.9% | 7.5% | 0.0% | 0.0% | 0.0% | 0.0% |
|  | A+T+N- | 8.5% | 7.8% | 7.9% | 7.5% | 3.0% | 29.8% | 16.8% | 62.5% |
|  | A+T+N+ | 4.5% | 40.4% | 26.7% | 75.0% | 0.0% | 0.0% | 0.0% | 0.0% |
| A2T2N3 | A-T-N- | 32.0% | 11.3% | 14.9% | 2.5% | 82.5% | 42.6% | 54.5% | 12.5% |
|  | A-T-N+ | 27.5% | 14.2% | 19.8% | 0.0% | 1.5% | 2.1% | 3.0% | 0.0% |
|  | A-T+N- | 7.5% | 6.4% | 6.9% | 5.0% | 1.5% | 7.8% | 7.9% | 7.5% |
|  | A-T+N+ | 11.5% | 6.4% | 7.9% | 2.5% | .5% | 4.3% | 3.0% | 7.5% |
|  | A+T-N- | 1.5% | 2.8% | 4.0% | 0.0% | 9.5% | 12.1% | 13.9% | 7.5% |
|  | A+T-N+ | 7.0% | 10.6% | 11.9% | 7.5% | 1.5% | 1.4% | 1.0% | 2.5% |
|  | A+T+N- | 2.0% | 5.0% | 5.0% | 5.0% | 2.0% | 23.4% | 13.9% | 47.5% |
|  | A+T+N+ | 11.0% | 43.3% | 29.7% | 77.5% | 1.0% | 6.4% | 3.0% | 15.0% |
| A2T2N4 | A-T-N- | 43.3% | 13.1% | 18.2% | 0.0% | 80.9% | 39.4% | 50.5% | 10.5% |
|  | A-T-N+ | 13.5% | 12.4% | 16.2% | 2.6% | 1.4% | 5.1% | 7.1% | 0.0% |
|  | A-T+N- | 16.3% | 5.1% | 7.1% | 0.0% | 2.8% | 7.3% | 9.1% | 2.6% |
|  | A-T+N+ | 5.0% | 8.0% | 8.1% | 7.9% | 0.0% | 5.1% | 2.0% | 13.2% |
|  | A+T-N- | 4.3% | 5.1% | 7.1% | 0.0% | 10.6% | 10.2% | 11.1% | 7.9% |
|  | A+T-N+ | 2.8% | 8.0% | 9.1% | 5.3% | 0.0% | 3.6% | 4.0% | 2.6% |
|  | A+T+N- | 9.2% | 13.1% | 14.1% | 10.5% | 4.3% | 17.5% | 14.1% | 26.3% |
|  | A+T+N+ | 5.7% | 35.0% | 20.2% | 73.7% | 0.0% | 11.7% | 2.0% | 36.8% |
| A2T2N5 | A-T-N- | 26.8% | 12.1% | 15.7% | 3.0% | 76.1% | 40.5% | 53.0% | 9.1% |
|  | A-T-N+ | 29.7% | 13.8% | 19.3% | 0.0% | 5.8% | 5.2% | 6.0% | 3.0% |
|  | A-T+N- | 9.4% | 3.4% | 4.8% | 0.0% | 2.9% | 11.2% | 9.6% | 15.2% |
|  | A-T+N+ | 11.6% | 10.3% | 10.8% | 9.1% | 0.0% | 2.6% | 2.4% | 3.0% |
|  | A+T-N- | 2.2% | 2.6% | 3.6% | 0.0% | 8.0% | 13.8% | 14.5% | 12.1% |
|  | A+T-N+ | 5.1% | 11.2% | 13.3% | 6.1% | 2.9% | 0.0% | 0.0% | 0.0% |
|  | A+T+N- | 6.5% | 10.3% | 9.6% | 12.1% | 4.3% | 19.8% | 10.8% | 42.4% |
|  | A+T+N+ | 8.7% | 36.2% | 22.9% | 69.7% | 0.0% | 6.9% | 3.6% | 15.2% |
| A2T3N1 | A-T-N- | 32.0% | 9.2% | 11.9% | 2.5% | 78.5% | 40.4% | 52.5% | 10.0% |
|  | A-T-N+ | 14.0% | 8.5% | 11.9% | 0.0% | 3.0% | 7.1% | 8.9% | 2.5% |
|  | A-T+N- | 18.5% | 6.4% | 7.9% | 2.5% | 4.5% | 2.1% | 3.0% | 0.0% |
|  | A-T+N+ | 14.0% | 14.2% | 17.8% | 5.0% | 0.0% | 7.1% | 4.0% | 15.0% |
|  | A+T-N- | 5.5% | 4.3% | 5.0% | 2.5% | 10.0% | 17.0% | 17.8% | 15.0% |
|  | A+T-N+ | 2.0% | 2.8% | 2.0% | 5.0% | 1.0% | 4.3% | 4.0% | 5.0% |
|  | A+T+N- | 9.0% | 8.5% | 9.9% | 5.0% | 3.0% | 9.9% | 6.9% | 17.5% |
|  | A+T+N+ | 5.0% | 46.1% | 33.7% | 77.5% | 0.0% | 12.1% | 3.0% | 35.0% |
| A2T3N2 | A-T-N- | 34.2% | 10.6% | 13.9% | 2.5% | 81.4% | 47.5% | 61.4% | 12.5% |
|  | A-T-N+ | 11.6% | 7.1% | 9.9% | 0.0% | 0.0% | 0.0% | 0.0% | 0.0% |
|  | A-T+N- | 19.6% | 9.2% | 11.9% | 2.5% | 4.5% | 9.2% | 6.9% | 15.0% |
|  | A-T+N+ | 13.1% | 11.3% | 13.9% | 5.0% | 0.0% | 0.0% | 0.0% | 0.0% |
|  | A+T-N- | 5.5% | 2.8% | 4.0% | 0.0% | 11.1% | 21.3% | 21.8% | 20.0% |
|  | A+T-N+ | 2.0% | 4.3% | 3.0% | 7.5% | 0.0% | 0.0% | 0.0% | 0.0% |
|  | A+T+N- | 9.0% | 9.2% | 9.9% | 7.5% | 3.0% | 22.0% | 9.9% | 52.5% |
|  | A+T+N+ | 5.0% | 45.4% | 33.7% | 75.0% | 0.0% | 0.0% | 0.0% | 0.0% |
| A2T3N3 | A-T-N- | 26.5% | 7.8% | 9.9% | 2.5% | 80.0% | 44.7% | 57.4% | 12.5% |
|  | A-T-N+ | 19.5% | 9.9% | 13.9% | 0.0% | 1.5% | 2.8% | 4.0% | 0.0% |
|  | A-T+N- | 13.0% | 9.9% | 11.9% | 5.0% | 4.0% | 5.7% | 5.0% | 7.5% |
|  | A-T+N+ | 19.5% | 10.6% | 13.9% | 2.5% | .5% | 3.5% | 2.0% | 7.5% |
|  | A+T-N- | 1.5% | .7% | 1.0% | 0.0% | 9.0% | 19.1% | 19.8% | 17.5% |
|  | A+T-N+ | 6.0% | 6.4% | 5.9% | 7.5% | 2.0% | 2.1% | 2.0% | 2.5% |
|  | A+T+N- | 2.0% | 7.1% | 7.9% | 5.0% | 2.5% | 16.3% | 7.9% | 37.5% |
|  | A+T+N+ | 12.0% | 47.5% | 35.6% | 77.5% | .5% | 5.7% | 2.0% | 15.0% |
| A2T3N4 | A-T-N- | 31.9% | 7.3% | 10.1% | 0.0% | 78.7% | 41.6% | 53.5% | 10.5% |
|  | A-T-N+ | 9.2% | 10.2% | 13.1% | 2.6% | 1.4% | 5.8% | 8.1% | 0.0% |
|  | A-T+N- | 27.7% | 10.9% | 15.2% | 0.0% | 5.0% | 5.1% | 6.1% | 2.6% |
|  | A-T+N+ | 9.2% | 10.2% | 11.1% | 7.9% | 0.0% | 4.4% | 1.0% | 13.2% |
|  | A+T-N- | 3.5% | 3.6% | 4.0% | 2.6% | 10.6% | 16.1% | 16.2% | 15.8% |
|  | A+T-N+ | 2.1% | 2.9% | 3.0% | 2.6% | 0.0% | 5.1% | 5.1% | 5.3% |
|  | A+T+N- | 9.9% | 14.6% | 17.2% | 7.9% | 4.3% | 11.7% | 9.1% | 18.4% |
|  | A+T+N+ | 6.4% | 40.1% | 26.3% | 76.3% | 0.0% | 10.2% | 1.0% | 34.2% |
| A2T3N5 | A-T-N- | 16.7% | 7.8% | 9.6% | 3.0% | 73.9% | 43.1% | 56.6% | 9.1% |
|  | A-T-N+ | 23.9% | 7.8% | 10.8% | 0.0% | 5.8% | 6.0% | 7.2% | 3.0% |
|  | A-T+N- | 19.6% | 7.8% | 10.8% | 0.0% | 5.1% | 8.6% | 6.0% | 15.2% |
|  | A-T+N+ | 17.4% | 16.4% | 19.3% | 9.1% | 0.0% | 1.7% | 1.2% | 3.0% |
|  | A+T-N- | 2.9% | 1.7% | 2.4% | 0.0% | 8.7% | 20.7% | 19.3% | 24.2% |
|  | A+T-N+ | 2.9% | 5.2% | 4.8% | 6.1% | 2.2% | 0.0% | 0.0% | 0.0% |
|  | A+T+N- | 5.8% | 11.2% | 10.8% | 12.1% | 3.6% | 12.9% | 6.0% | 30.3% |
|  | A+T+N+ | 10.9% | 42.2% | 31.3% | 69.7% | .7% | 6.9% | 3.6% | 15.2% |

# Supplementary Figures

## Supplementary Figure 1


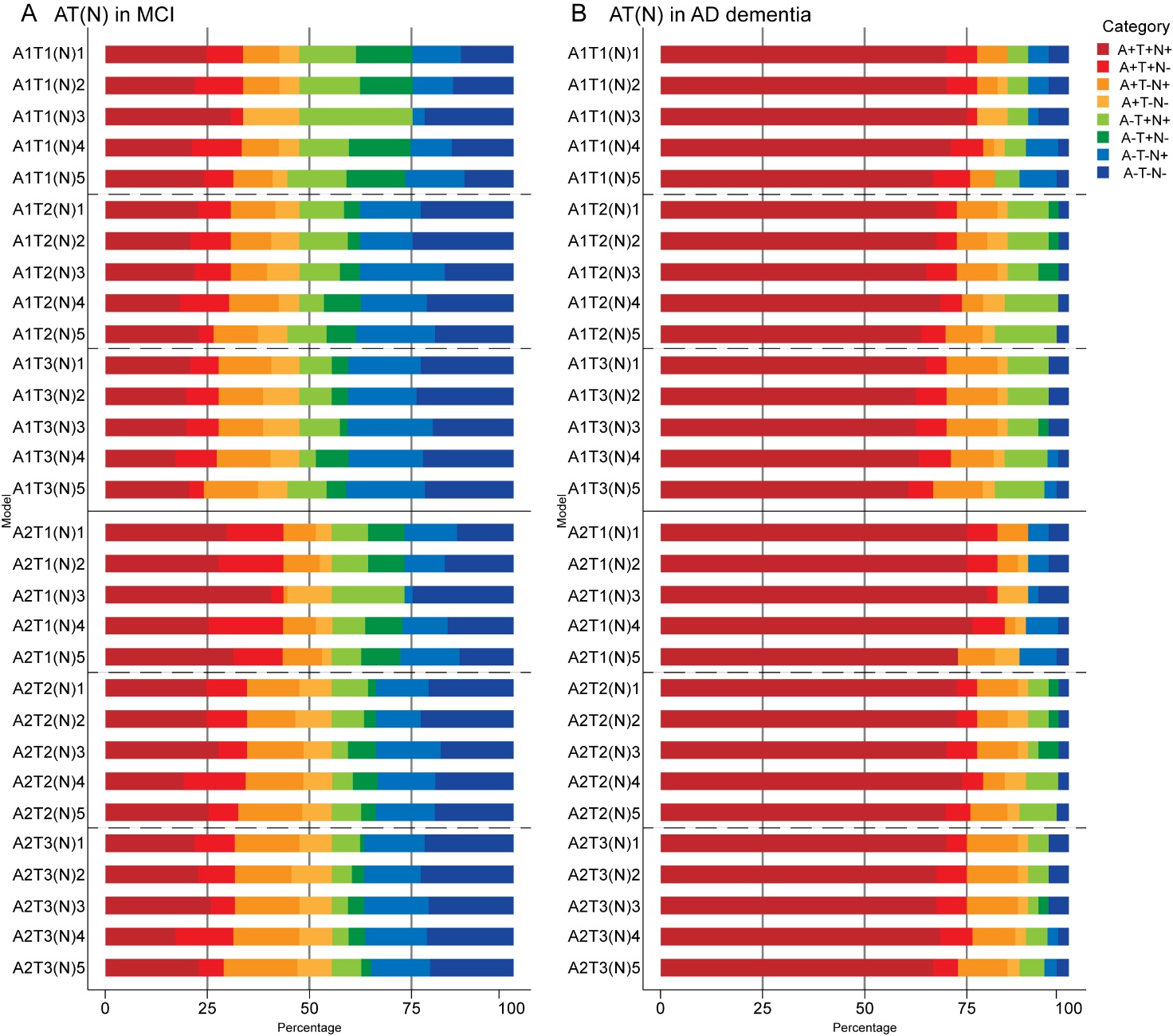


**Supplementary Figure 1.** Prevalence of different AT(N) categories in different AT(N) variants among mild cognitively impaired (MCI) (A) and Alzheimer’s disease (AD) (B) participants in the ADNI. CSF Aβ42 (A1); amyloid PET whole cerebellum standardized uptake value ratio (SUVR) (A2); CSF tau phosphorylated at Thr181 (T1); tau PET inferior temporal cortex SUVR (T2); tau PET Braak Ⅴ/Ⅵ SUVR (T3); hippocampal volume [(N)1]; temporal meta-ROI thickness [(N)2]; CSF total tau [(N)3]; FDG PET meta-ROI SUVR [(N)4]; plasma neurofilament light [(N)5]. AT(N) = β-amyloid, tau and neurodegeneration classification system.
